# Data-Driven Workflow for the Development and Discovery of *N*-Oxyl Hydrogen Atom Transfer Catalysts

**DOI:** 10.1021/acscentsci.4c01919

**Published:** 2025-03-24

**Authors:** Cheng Yang, Thérèse Wild, Yulia Rakova, Stephen Maldonado, Matthew S. Sigman, Corey R. J. Stephenson

**Affiliations:** ^†^Department of Chemistry, ^#^Department of Biochemistry and Molecular Biology, University of British Columbia, Vancouver, BC V6T 1Z1, Canada; ‡ Department of Chemistry, University of Michigan, Ann Arbor, Michigan 48109, United States; § Program in Applied Physics, University of Michigan, Ann Arbor, Michigan 48109, United States; ∥ Department of Chemistry, University of Utah, Salt Lake City, Utah 84112, United States

## Abstract

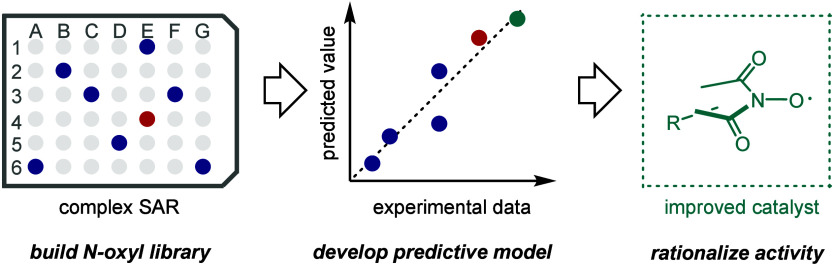

*N*-oxyl species are promising hydrogen atom transfer (HAT) catalysts to advance C–H bond activation reactions. However, because of the complex structure–activity relationship within the *N*-oxyl structure, catalyst optimization is a key challenge, particularly for simultaneous improvement across multiple parameters. This paper describes a data-driven approach to optimize *N*-oxyl hydrogen atom transfer catalysts. A focused library of 50 *N*-hydroxy compounds was synthesized and characterized by three parameters—oxidation peak potential, HAT reactivity, and stability—to generate a database. Statistical modeling of these activities described by their intrinsic physical organic parameters was used to build predictive models for catalyst discovery and to understand their structure–activity relationships. Virtual screening of 102 synthesizable candidates allowed for rapid identification of several ideal catalyst candidates. These statistical models clearly suggest that *N*-oxyl substructures bearing an adjacent heteroatom are more optimal HAT catalysts compared to the historical focus, phthalimide-*N*-oxyl, by striking the best balance among all three target experimental properties.

## Abstract 2

Machine learning models revealed that *N*-oxyl compounds bearing adjacent heteroatoms to carbonyls are more promising hydrogen atom transfer catalysts by striking a balance between multiple parameters.

## Introduction

**Figure 1:**
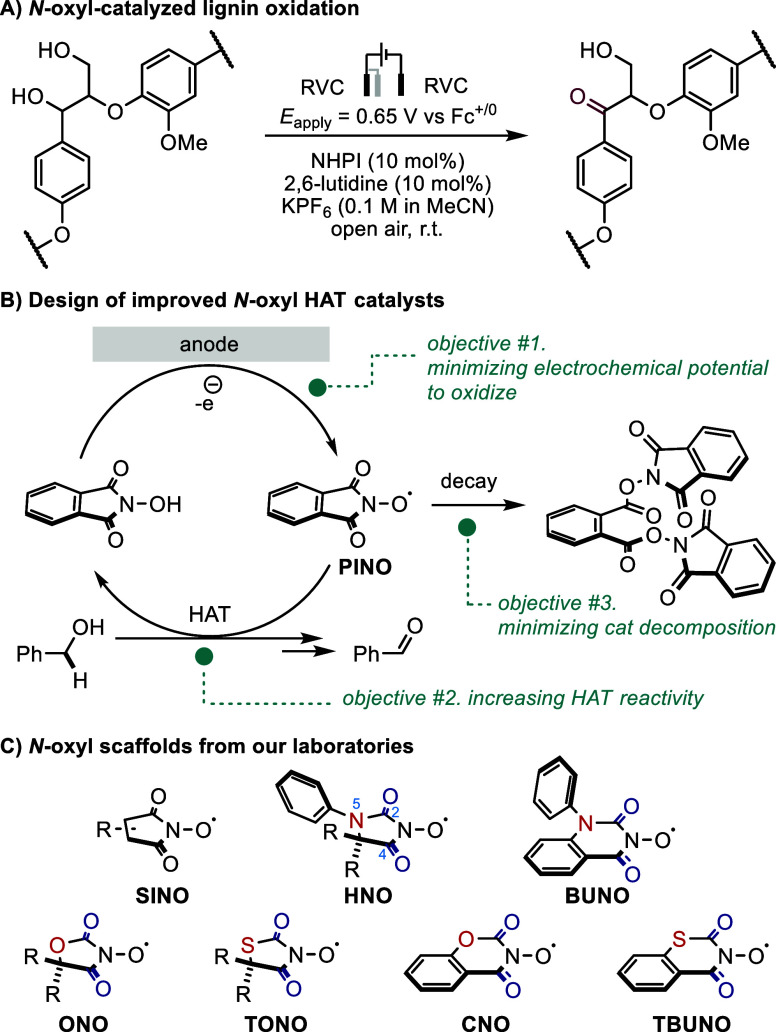
*N*-oxyl type of HAT catalysts.

*N*-oxyl species are hydrogen atom transfer (HAT) electrocatalysts valuable for selective oxidation of C–H bonds,^1,2^ including those present in biomass such as lignin (Figure 1A).^3^ Although promising overall, several catalytic aspects of *N*-oxyl HAT species require improvement before practical applications of these catalysts. Specifically, a catalyst for selective oxidation should meet at least three criteria (Figure 1B): 1) Catalyst generation must occur at modest positive potentials, both to avoid parasitic side oxidation reactions and to minimize energy usage; 2) the desired HAT reactivity must be efficient to ensure useful conversion rates; 3) the catalyst must be stable to maintain its overall performance, especially on a biomass processing scale.

In the last two decades, several advances have been made to improve *N*-oxyl HAT catalysts.^4,5^ A bottleneck is how to improve performance across all three criteria simultaneously. The first challenge comes from the lack of tunable catalyst candidates. To date, the design of *N*-oxyl catalysts has focused on the functionalization of the phthalimide-*N*-oxyl (PINO) scaffold.^4−6^ The number of sites on the aromatic ring that can be modified is limited. The second challenge arises from the observation that gains in one performance criterion usually occur at the expense of the others. For example, previous studies have demonstrated that adding electron-withdrawing functionalities to the phthalimide core^7^ often improves HAT reactivity but to the detriment of its stability.^8^

In this context, our laboratories have made meaningful progress by designing new scaffolds and standardizing evaluation methods (Figure 1C).^9−12^ By investigating the mechanism of PINO-catalyzed benzylic alcohol oxidation and the catalyst decomposition pathways, our results suggest that carbonyls C2=O and C4=O are important for maintaining its HAT reactivity, meanwhile their electrophilicity is also responsible for catalyst decomposition.^9^ This insight inspired several synthetic campaigns to reveal various new scaffolds bearing a heteroatom adjacent to the reactive site. We found that a neighboring nitrogen N5 to the carbonyl C2=O significantly stabilizes the catalysts without significantly shifting the potential,^10^ while a nearby oxygen or sulfur dramatically improves the reactivity through tuning of the bond dissociation enthalpy.^11^ Furthermore, we have applied a potential-controlled electrochemical approach to evaluate *N*-oxyl catalysts and found that succinimide-*N*-oxyl (SINO) derivatives are in fact active HAT catalysts in contrast to previous reports.^12^ Leveraging the synthetic accessibility to these new scaffolds and the complex structure–activity relationships within the *N*-oxyl moiety, we posit that data-driven approaches can 1) provide mechanistic insights; 2) accelerate the discovery of potential *N*-oxyl catalysts; and 3) enhance related studies on data science enabled investigation on HAT.^13−15^

We hypothesized that further development of *N*-oxyl HAT catalysts could be realized through statistical modeling, similar to those that have recently been employed for catalyst and battery electrolyte optimization.^16,17^ These data science-powered methods can accelerate the optimization of new catalyst designs by revealing the relationship between structural properties and the objectives of interest (e.g., redox potential, catalytic activity, and stability). These models can inform strategies such as scaffold hopping from one core structure to alternatives offering new opportunities for identifying active candidates. However, the generation of robust and predictive models requires a diverse data set for training and validation. Thus, the limited chemotypes reported outside of the parent PINO scaffold would not provide sufficient diversity to effectively apply a data science-based approach.

Herein, we present the synthesis and catalytic evaluation of a library of *N*-oxyl compounds to generate predictive and interpretable statistical models for catalytic activity, redox potential, and stability. These models were leveraged to predict and understand the important features to balance our three objectives in identifying a new set of HAT catalysts with desirable properties.

## Results and Discussions

### Building of Training Set: Synthesis and Evaluation

First, we synthesized a set of known NHPI-derivatives according to published reports. These compounds include 1) NHPI itself; 2) a more reactive species tetrachloro-substituted NHPI; 3) a more stable species tetraphenyl-substituted NHPI; 4) a heterocyclic species pyrido-NHPI; and 5) two *N*-hydroxyl-naphthalimides. Although several catalyst candidates bearing other electron-withdrawing groups instead of carbonyls have been previously reported,^18−21^ these scaffolds were not considered in this study to maintain consistent catalyst substructures to simplify parametrization (vide infra). The generation of meaningful and predictive statistical models requires diverse data inputs, however functionalization of NHPI only provides a few *N*-hydroxy compounds. This motivated us to develop synthetic methods to rapidly access various *N*-hydroxyl compounds with two neighboring carbonyls.

**Figure 2:**
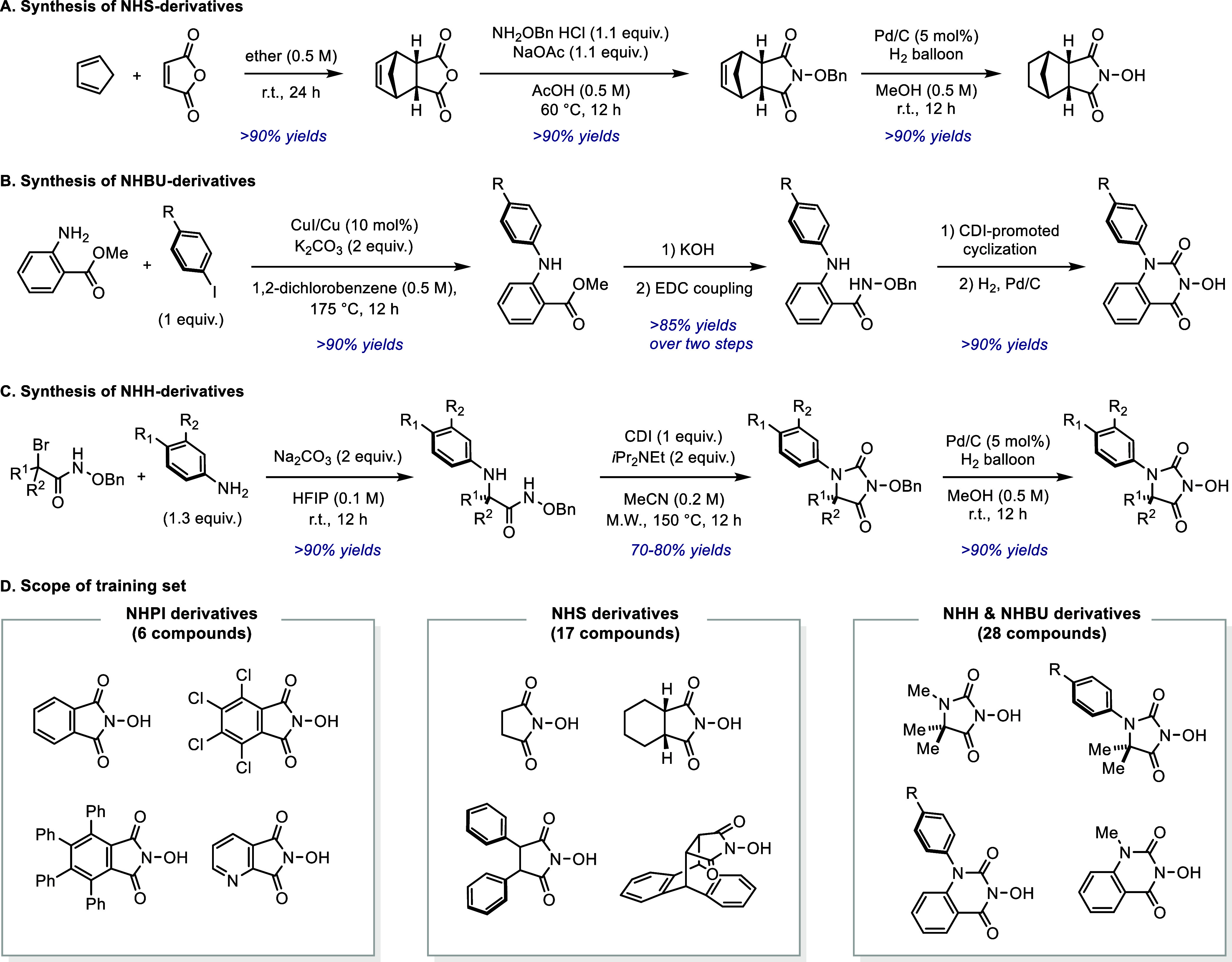
Building a *N*-hydroxyl compound library. Synthetic routes A and C were designed according to refs (12) and (10), respectively.

It is important to note that the direct oxidation of amines using oxiranes, oxadiazines, peroxides, as well as peracids cannot generally be operated on preparative scale to form *N*-hydroxy compounds. Instead, the *N*-hydroxy motif is often prepared through condensation with *O*-protected hydroxylamine. Thus, we targeted Diels–Alder reactions between maleic anhydride and various dienes to prepare new catalysts,^12^ leveraging the ready availability of the requisite starting materials. Upon optimization, we found that the condensation between anhydrides and *O*-benzylhydroxylamine hydrogen chloride was effective with mild heating (60 °C) in acetic acid (generally >90% yield) (Figure 2A).^12^ When the reaction was complete, the product was precipitated by adding 10 volume times of water, and the filtration cake was pure enough to be directly used in the subsequent step without the need of purification. The operational simplicity of this process streamlined the synthesis of our catalyst candidate precursors. Because PINO is known to react with olefins,^23^ we subjected the condensation intermediate to Pd/C-catalyzed hydrogenation to simultaneously reduce the alkene and remove the benzyl protecting group in one pot. This synthetic sequence was routinely operated on gram scale.

On the basis of our previous efforts to design *N*-oxyl HAT catalysts, the training set also includes a range of *N*-hydroxy compounds bearing a heteroatom (N, O, or S) adjacent to one carbonyl. The addition of these new molecules provides more structural diversity, therefore broadening the scope of our structure–function studies. Figure 2B demonstrates a general synthetic pathway to access substituted *N*-hydroxybenzouracils (NHBU). The synthesis was initiated through a CuI/Cu-catalyzed amination between methyl anthranilate and substituted iodobenzenes, which, upon saponification of the ester, were readily subjected to an amide-coupling to introduce the hydroxylamine motif. Cyclization promoted by carbonyldiimidazole afforded the benzouracil motif. Finally, the benzyl group was removed under reductive conditions. Similar to our previous report,^10^*N*-hydroxyhydantoin (NHH) were synthesized using the amination/cyclization/deprotection sequence shown in Figure 2C. In total, these new compounds together with a few known NHPI derivatives yielded a collection of 50 distinct *N*-hydroxyl molecules (Figure 2D).

**Figure 3:**
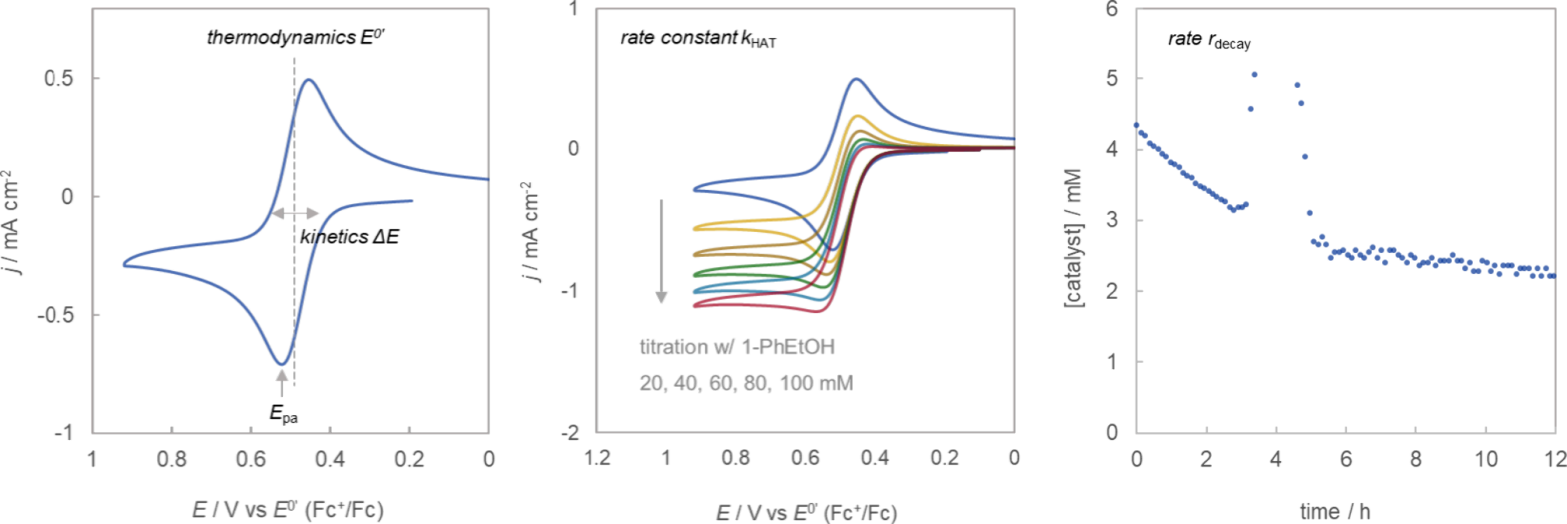
Evaluation of *N*-oxyl HAT catalysts. Tabulated data for all catalysts are given in the SI.

With the training set in hand, three separate experimental protocols were executed to ascertain their performance metrics (Figure 3). First, two related parameters were determined to assess how the generation of each *N*-oxyl catalyst occurs electrochemically: formal potential (*E*^0^′) and anodic peak potential (*E*_pa_). Although the operating base can impact the potential by ∼59 mV/p*K*_a_ due to a proton-coupled electron transfer mechanism,^9,24^ pyridine was used as the base for cyclic voltammetry measurement, no matter the acidity of the protons. Second, the HAT reactivity (*k*_HAT_) was evaluated by cyclic voltammetric titration. During these studies, we found that the shapes of the steady-state catalytic current response were slightly different, but all could be fit to a second-order rate equation, thereby providing reasonable comparisons of HAT kinetics. We determined that exogenous bases did not impact the measured *k*_HAT_ as they are likely dissociated from *N*-oxyls after catalyst generation. For simplicity and consistency, pyridine was used throughout the kinetic experiments. Third, the initial rate of catalyst decay, *r*_decay_, was determined by (Flow)NMR as previously described.^10^ All tabulated data are presented in the Supporting Information (SI). Both *k*_HAT_ and *r*_decay_ were normalized by the respective values for NHPI.

### Generation of Statistical Models

To evaluate the impact of computed structural properties on our target experimental objectives, we utilized density functional theory (DFT)-based catalyst descriptors to develop interpretable statistical models. Properties were collected for each catalyst structure both as the catalyst precursor (ground state) and catalyst (radical state) to facilitate more complete analysis of reactivity.

**Figure 4:**
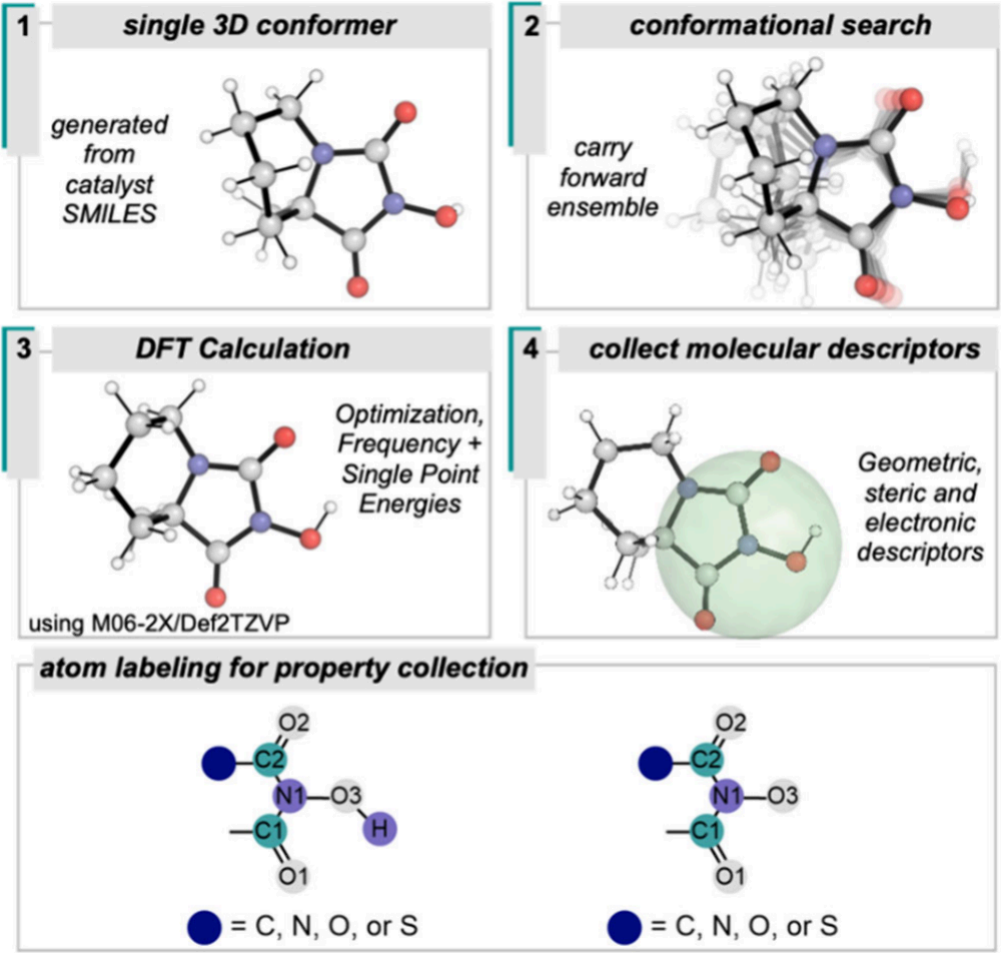
Workflow for calculation of catalysts and molecular descriptor collection.

As a first step, MacroModel^22^ was utilized to perform a conformational search yielding a conformational ensemble within a 5.0 kcal/mol range for each (Figure 2B). DFT gas phase geometry optimization and frequency calculations were performed on each conformer utilizing the Gaussian program^23^ with the M06-2X functional and Def2TZVP basis set. Additional single point energy calculations were performed on the optimized structures using the same level of theory to obtain further electronic descriptors. For each catalyst, descriptors for both the precursor and catalyst were considered in statistical modeling. Descriptors were collected for atoms directly involved in catalysis or adjacent to the active site (Figure 4). Several types of point charges as well as atom dipoles were utilized in addition to bond energy, occupancy, and strength measures to describe atoms of interest. Furthermore, several molecular steric descriptors describing the volume and size of the catalyst were used. Given the mode of catalyst activation, we also calculated the bond dissociation free energy (BDFE) for the lowest energy conformer of each structure.

**Figure 5:**
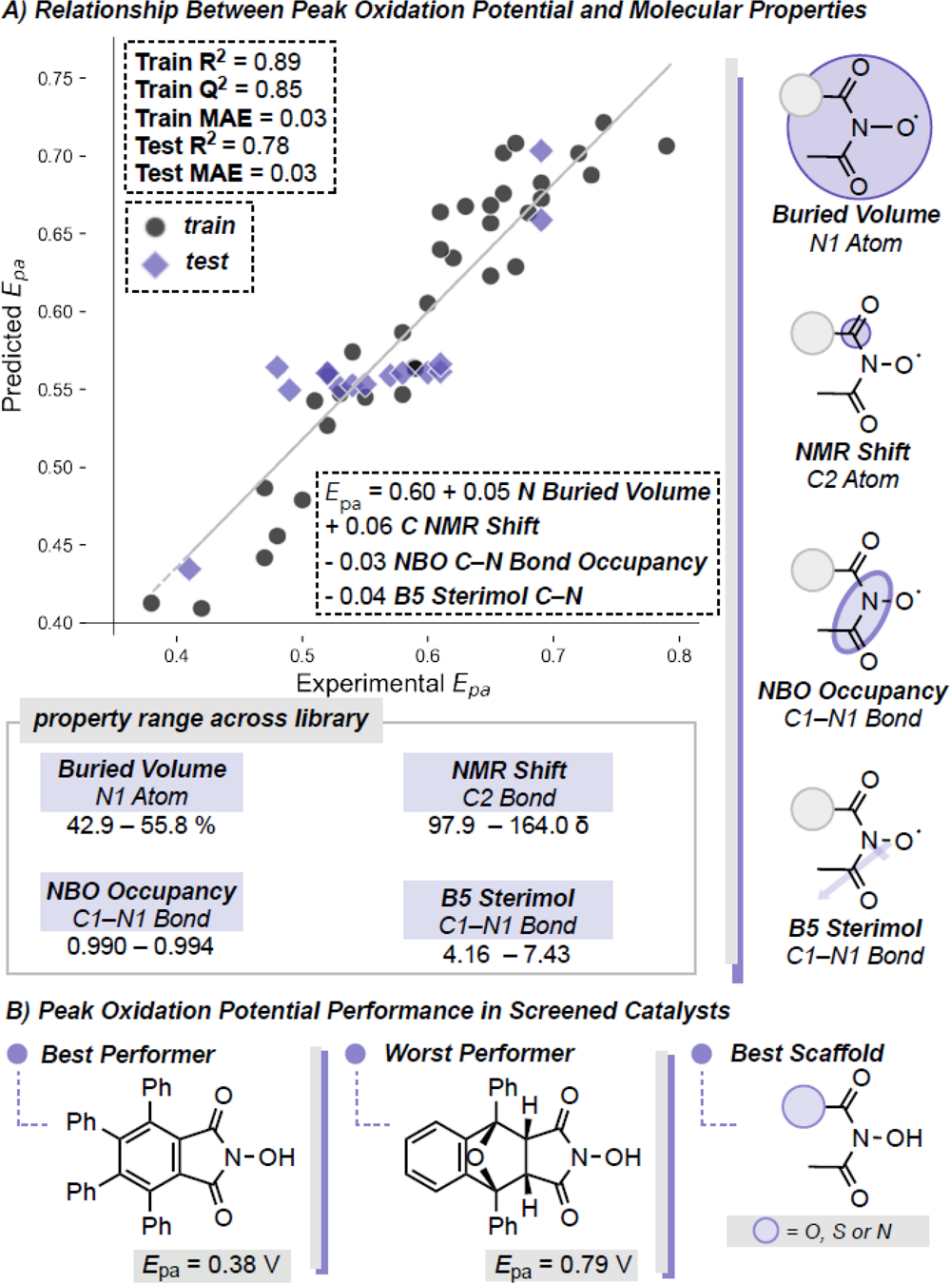
Regression model explaining the relationship between catalyst properties and peak potential.

For each target objective (*E*_pa_, *k*_HAT_, and *r*_decay_), we evaluated several types of algorithms to build correlations including regression and classification. We first investigated how the structural features extracted from the DFT calculations correlated to the potential for oxidation of the catalyst. This was accomplished by regressing (multivariable linear regression) catalyst descriptors against *E*_pa_ as this experimental measure was more consistently accessible than *E*^0^′. Statistical models were produced by first splitting the data into a training set (70% of data) and a test set (30% of data). If multiple models were produced for each objective, these models were downselected first to include only those that met our standards for statistical performance (Training and Test *R*^2^ > ∼0.80). From these models, the most mechanistically relevant and interpretable model was selected. Further details on model construction and selection may be found in the SI. A representative model is depicted in Figure 5 that was considered statistically robust based upon the similarities between the *R*^2^ (0.89) and *Q*^2^ (0.85) for the training data as well as the agreement of *R*^2^ (0.78) and mean absolute error (MAE) (0.03 V) for the test set. The model contained four parameters all derived from the catalyst and include: the buried volume from the N1 atom, the anisotropic NMR shift at the C2 carbon atom, the natural bonding orbital (NBO) occupancy for the C1–N1 bond and the B5 Sterimol value for the C1–N1 bond (Figure 5A). All terms contribute to the model relatively equally. The model suggests that lower percent buried volumes, greater bond occupancy and higher Sterimol values minimize *E*_pa_ and, thus, leads to the optimal catalyst structures for this objective. While these trends are generally observed, relationships are complex. This is highlighted by the difference between the top performing catalyst (Figure 5A) in this category, which has a percent buried volume (N1 atom) of 45.5%, a B5 Sterimol value (C1–N1 bond) of 7.39 and an NBO occupancy (C1–N1 bond) of 0.993. The lowest-performing catalyst (Figure 5B), in contrast, has a percent buried volume of 55%, a B5 Sterimol value 6.39 and an NBO occupancy of 0.994. Although it is somewhat surprising to find that steric terms, such as buried volume, are impactful in predicting *E*_pa_, this term is likely describing catalyst size. We propose this could be reading out the ability of the catalyst to stabilize the oxyl radical. While these differing catalysts represent the full range of buried volume, the other values are not the extrema for the descriptor. The final parameter, the computed anisotropic shift on the C2 atom, is somewhat more difficult to interpret. However, we find that this parameter is highly correlated with the Hirshfeld charge on the N1 atom (alternative model shown in the SI) (*R*^2^ = 0.81), which suggests that an increase in charge on this atom is associated with more positive *E*_pa_ values. Generally, we can assume that with greater deshielding of this carbon, *E*_pa_ becomes more positive; however, anisotropic shift rather than isotropic shift being the correlated descriptor here could emphasize the importance of conformation in impacting the value of *E*_pa_.

**Figure 6:**
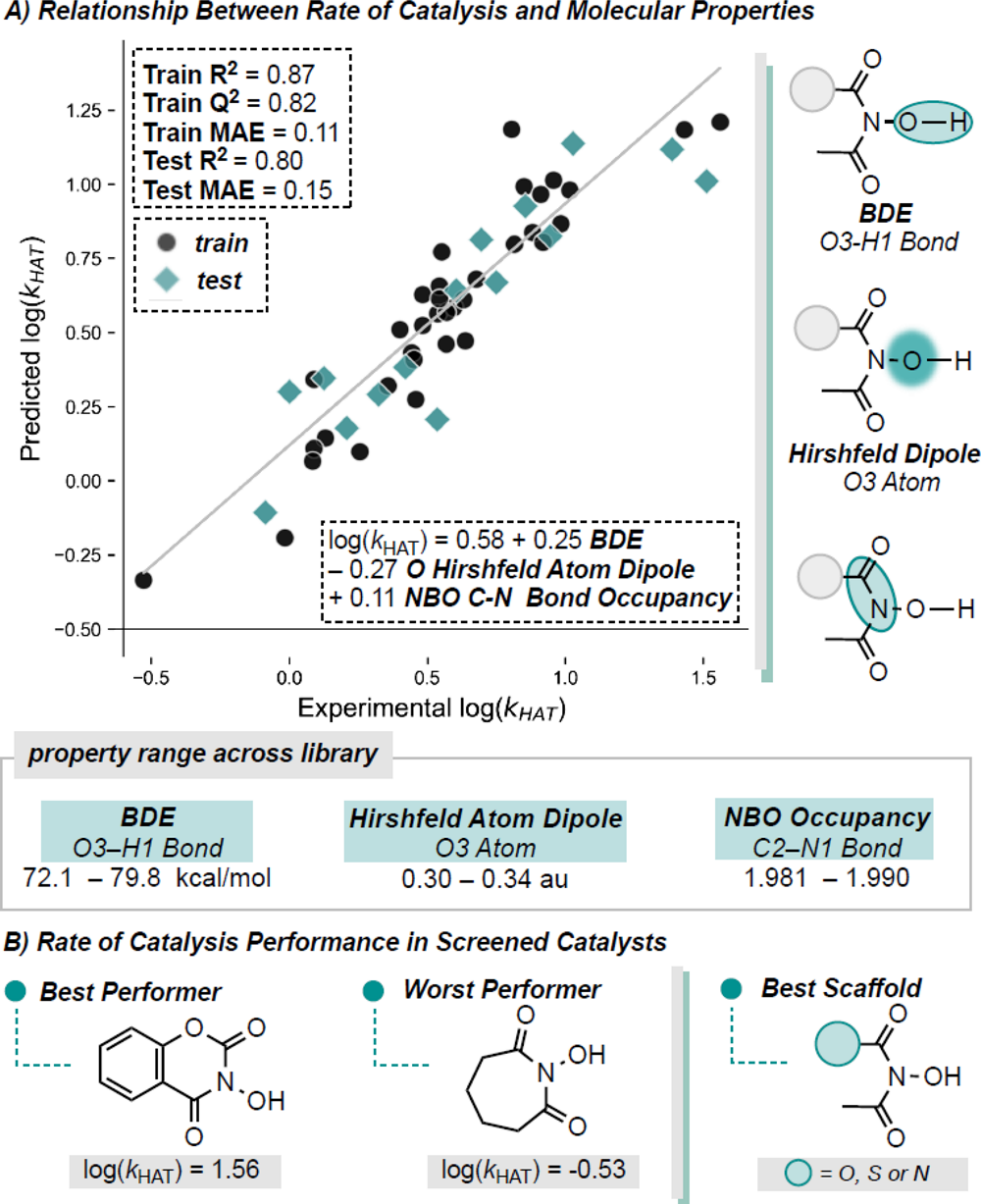
Statistical model explaining relationship between catalyst descriptors and rate of HAT catalysis.

Our next experimental target for optimization was the rate of catalysis, *k*_HAT._ As above, the data was divided into training and test sets using the y-equidistant algorithm and a 70/30 training/test split. For model building, log(*k*_HAT_), rather than *k*_HAT_, was regressed against catalyst descriptors using multivariable linear regression (MLR). All relevant models are detailed in the SI, with one representative model depicted in Figure 6A.

For our selected model, we observed a training *R*^2^ value of 0.87 and a test *R*^2^ value of 0.80. Our training MAE for log(*k*_HAT_) is 0.11 while the test set has an MAE of 0.15. We found that the calculated BDFE of the O–H bond is directly proportional to the rate of catalysis such that an increase in BDFE is correlated to an increase in *k*_HAT,_ which is consistent with previous studies.^23^ Specifically, HAT catalysis between PINO type radicals and hydrocarbons has been reported to follow the Bell-Evans-Polanyi principle and are mildly exothermic reactions.^24^ Although polarity matching in the HAT transition state also plays an important role,^12^ BDFE seems to be a good descriptor to establish a reliable and predictive model. Additionally, the model indicates that an increasing Hirshfeld dipole on the O3 atom correlates to a decline in *k*_HAT_ meaning that catalysts with less polarization on this atom enhance *k*_HAT_. The final parameter in this MLR model was the NBO bond occupancy of the C2–N1 bond such that an increase in occupancy is associated with an increase in the rate of catalysis. In this model, the coefficients for BDFE and Hirshfeld atom dipole are nearly equal indicating that these two descriptors are the most important and have a similar impact on the rate of catalysis. In the best-performing catalyst (Figure 6B), the calculated BDFE for the bond of interest is 79.1 kcal/mol while the poorest-performing catalyst (Figure 6B) has a BDFE of 75.2 kcal for this same bond. For the Hirshfeld atom dipole, the highest-performing catalyst has a dipole of 0.32 while the lowest-performing catalyst has a dipole of 0.34.

**Figure 7:**
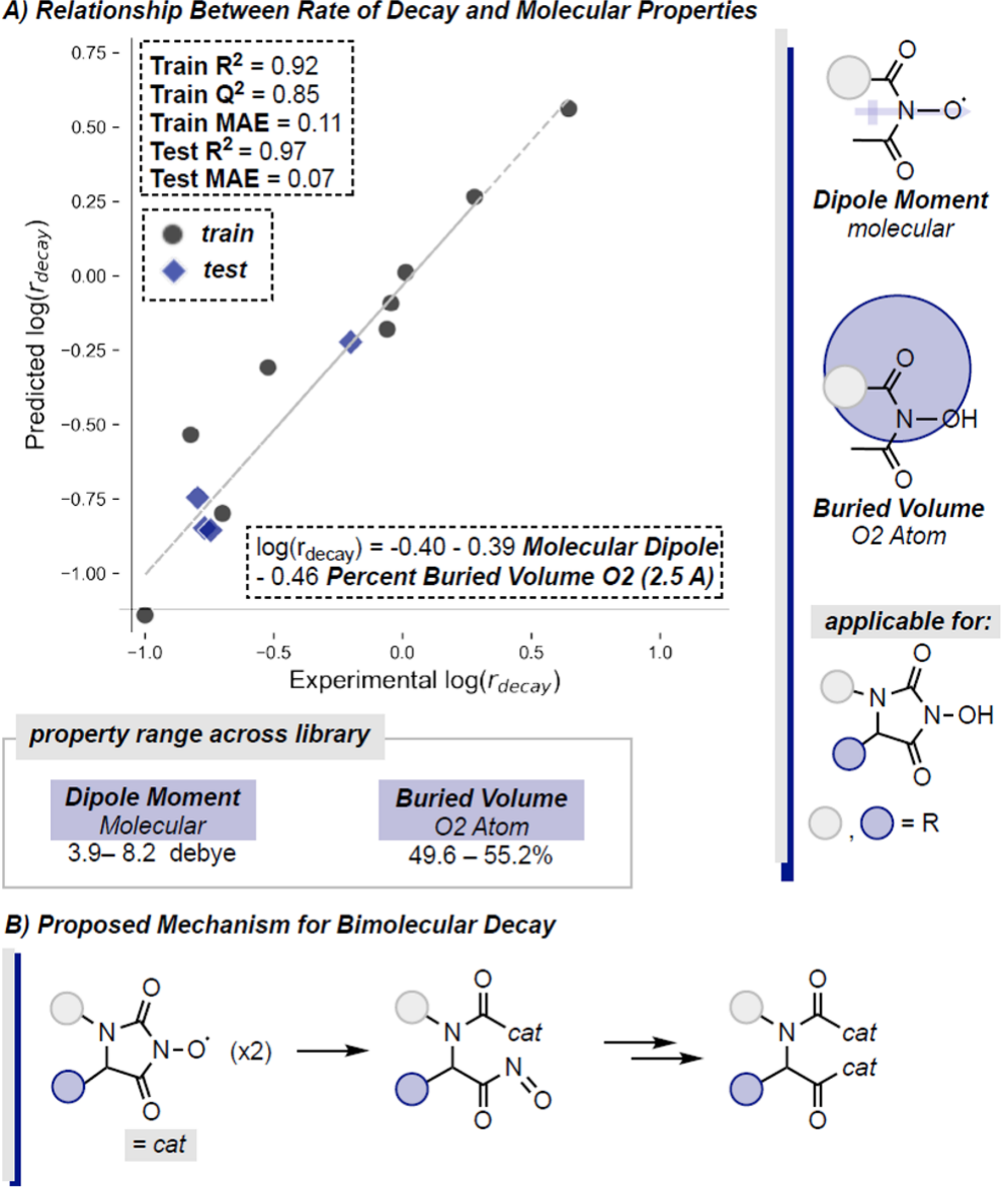
Regression model for the rate of decay; applicable to structures where bimolecular decay is the proposed major decay pathway.

**Figure 8:**
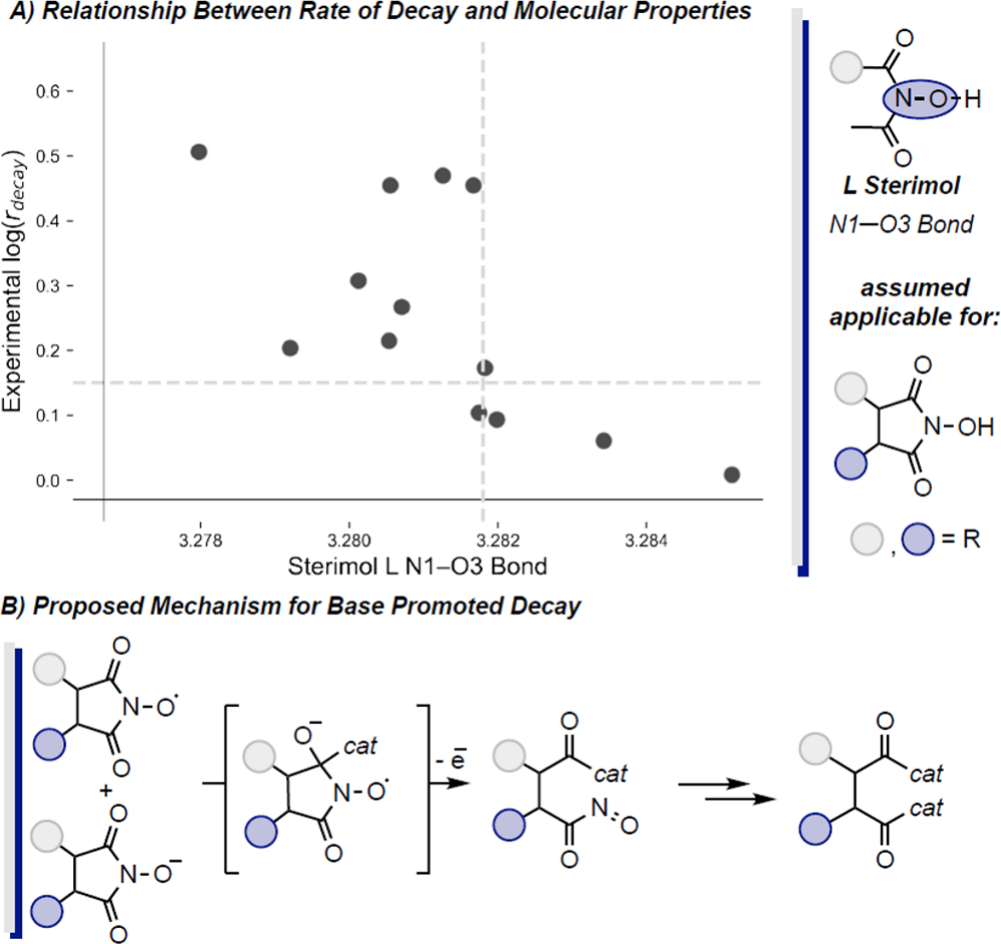
Classification for rate of decay; applicable to structures where base-promoted decay is the proposed major decay pathway.

Our final target, *r*_decay_, proved more challenging to build statistical models using the entire data set. At times, linear modeling efforts can fail due to competing mechanisms which cannot be explained by an overlapping set of parameters. We hypothesized that the challenge modeling this data was due to the fact there are likely multiple accessible decay mechanisms, making determination of a single linear model difficult. Thus, we focused on two major decomposition mechanisms unveiled in our previous mechanistic study,^9^ which result from different catalyst substructures: 1) decomposition through a bimolecular radical addition process (Figure 7B) and 2) base-promoted decomposition via nucleophilic attack (Figure 8B). It is proposed that the major decomposition pathway is a function of catalyst substructure; therefore, we partitioned the decomposition data set into two subsets by structure and proposed major mechanism of decay. For those proposed as decaying primarily via the bimolecular process, we found an MLR model demonstrating that a reduced percent buried volume for the O2 atom in the precatalyst as well as smaller dipole moment in the active catalyst resulted in an enhanced rate of decay (Figure 7A). This observation suggests that steric bulk, or more precisely substituent size, slows a bimolecular reaction thereby increasing catalyst stability. The appearance of descriptors for both the catalyst precursor and catalyst are indicative of the complex factors controlling catalyst decay. For the proposed base-promoted decay mechanism (Figure 8B), we could not locate an adequate linear model due to poor data distribution. However, a classification model was found demonstrating increased Sterimol L values for the N1–O3 bond atom of the precatalyst resulted in lower decay rates (Figure 8A).

**Figure 9:**
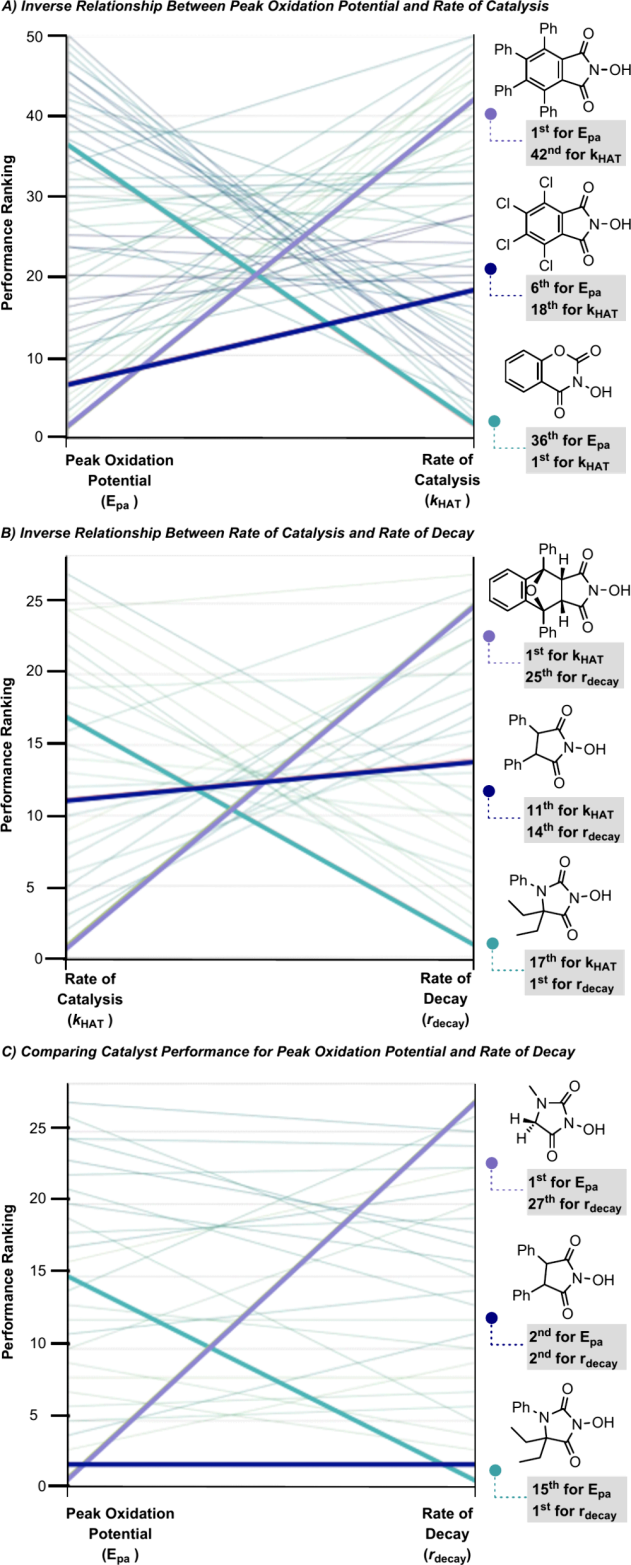
Demonstration of the relationships between each objective based on ranked catalyst performance in each objective.

Upon identifying models for all objectives, further analysis of these targets confirmed that simultaneous optimization of the rate of catalysis and oxidation peak potential is challenging as they are inversely correlated. In other words, catalysts that have the highest observed catalysis rates also require the most positive potential to generate. This relationship is further demonstrated in Figure 9A wherein structures were ranked in ascending order, such that the most optimal value in each objective is ranked first. As demonstrated, many of the catalysts that have the highest rates of catalysis are the most difficult to generate (have the highest *E*_pa_). This can also be intuitively concluded from the relationships discovered in the MLR models. The rate of catalysis increases when BDFE increases; however, an increase in BDFE indicates greater difficulty in generating the active catalyst. This relationship informs how we should pursue a virtual screening campaign as we need to balance all three objectives, not focus on a singular metric. It is also demonstrated that as rate of catalysis increases (better performer), rate of decay also increases (worse performer) (Figure 9B). Importantly, the relationship between *E*_pa_ and *r*_decay_ was more congruent (Figure 9C). While an inverse relationship is at times observed between *E*_pa_ and *r*_decay_, this is not always the case. As shown, it is possible to balance the two terms to achieve near ideal performance for both metrics with a single catalyst.

### Discovery of Improved *N*-Oxyl Catalysts: Virtual Screening and Experimental Validation

Taking the synthetic accessibility of *N*-hydroxy compounds into consideration and the statistical models, 102 proposed catalysts were virtually screened. This set of catalysts included substituted NHPIs, NHHs, NHBUs, and NHSs, which were parametrized using identical procedures to those described for experimental catalysts. Utilizing the statistical models we developed, we predicted *E*_pa_, *k*_HAT,_ and *r*_decay_ for this virtual catalyst descriptor library.

**Figure 10:**
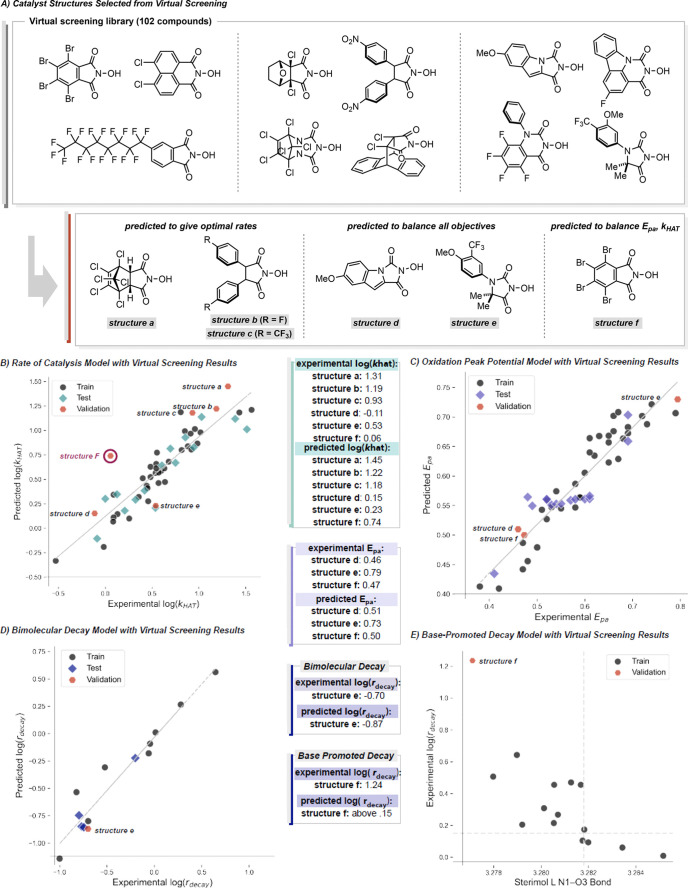
Results of virtual screening.

The goal of this virtual screen was 1) to identify catalyst structures that optimize and balance all three experimental objectives and 2) to support the relationships identified in the statistical models. Structures **a**, **b**, and **c** (Figure 10A) were selected as potentially optimal catalysts for reactivity. Structures **d** and **e** were selected as options that balance all three catalyst criteria. Structure **f** was selected as a means of balancing *E*_pa_, and *k*_HAT_.

All structures therefore were tested for reactivity, with four of the five confirming the predictions (Figure 10B). Structure **f** was more poorly predicted; presumably, this is due to difficulties in measuring rate of catalysis for species with very high rates of decay (*r*_decay_ = 17.2). Structures **d**, **e** and **f** were evaluated for *E*_pa_ with reasonable accuracy (Figure 10C). Structure **e**, the most poorly predicted, is at the limit of *E*_pa_ values in our data set and thus likely at the edge of the domain of applicability.

With respect to the rate of decay, structure **e** was used to validate the bimolecular decay model and structure **f** was used to validate the base promoted decay model. Structure **e** was predicted by the model with reasonable accuracy (Figure 10D) and indeed achieved a desirable balance of all three objectives. The model for base-promoted decay correctly predicts the rate of decay for structure **f**. Among all the structures included in this work, we found that hydantoin-*N*-oxyls offered the best balance across all three experimental objectives. While this subtype of catalyst is not the best performing in a single category it does not compromise any aspect.

## Conclusions

**Figure 11:**
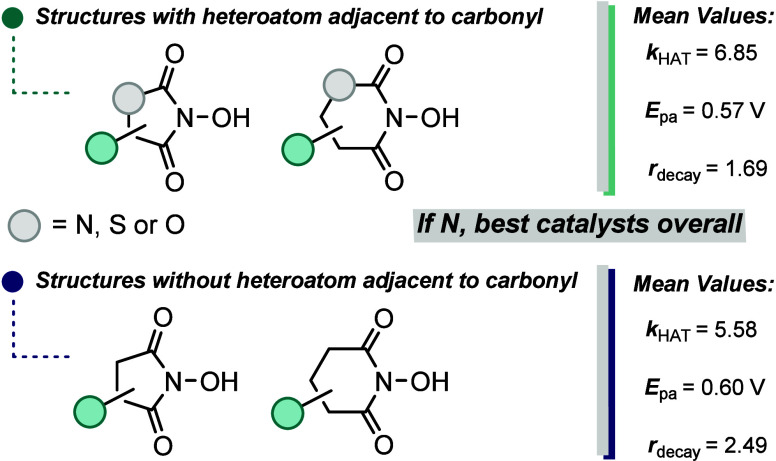
Average catalyst performance by scaffold.

In summary, we developed a series of predictive models to evaluate various parameters for *N*-oxyl HAT catalysts virtually. Although the physical organic parameters of several *N*-oxyl species have been investigated independently, systematic analysis remains elusive. Using a standardized method to evaluate *N*-oxyls and building predictive models, for the first time, this work describes the correlations among redox potential, catalysis, and stability. The use of statistical modeling allowed us to identify which catalyst descriptors most impacted our target properties. Furthermore, an inverse relationship between the optimization of the rate of catalysis and the potential for electrochemical oxidation was experimentally confirmed. Taking together a virtual screening of 102 catalyst candidates, the data presented herein clearly suggest that the substructure bearing a heteroatom neighboring to a carbonyl is a superior candidate (Figure 11), as opposed to the historical focus, phthalimide-*N*-oxyl derivatives. Structures like hydantoin-*N*-oxyls and benzouracil-*N*-oxyls deserve more attention in future studies on the design of *N*-oxyl catalysts and reaction development using similar species.

## Supporting Information Available

Experimental details, synthetic procedures, compound characterization, and spectra of all unknown compounds (PDF)
Virtual library of calculated properties (XLSX)
Calculated properties and experimental data (XLSX)
Optimized .xyz files (ZIP)
Analysis of all models (XLSX)
Modeling properties with reaction outcomes (XLSX)
