# Supplementary material for: Data-Driven Workflow for the Development and Discovery of N-Oxyl Hydrogen Atom Transfer Catalysts
Source: ACS Cent Sci. 2025 Mar 24;11(4):592–600. doi: 10.1021/acscentsci.4c01919 (PMC12022910; doi:10.1021/acscentsci.4c01919)
Supplement: Supplementary file 1 — oc4c01919_si_001.pdf [file oc4c01919_si_001.pdf]

Supporting information for

Data-driven Workflow for the Development and Discovery of *N*-oxyl Hydrogen Atom Transfer Catalysts

Cheng Yang,[b,△] Thérèse Wild,[d,△] Yulia Rakova,[b] Stephen Maldonado,*[b,c] Matthew S. Sigman,*[d] and Corey R. J. Stephenson*[a]

[a] Department of Chemistry, University of British Columbia, Vancouver, BC, V6T 1Z1, Canada.

[b] Department of Chemistry, University of Michigan, Ann Arbor, Michigan 48109, United States.

[c] Program in Applied Physics, University of Michigan, Ann Arbor, Michigan 48109, United States.

[d] Department of Chemistry, University of Utah, Salt Lake City, Utah 84112, United States.

* Email: stephenson@chem.ubc.ca; sigman@chem.utah.edu, and smald@umich.edu.

△ These authors contributed equally to this work.

Contents

I. General information

II. Molecular structures of the training set

III. Procedures for evaluating electrocatalysts

IV. Statistical modeling

V. Virtual screening

VI. Molecular structures of the virtual screening library

VII. Synthesis of *N*-hydroxy compounds

VIII. References

IX. NMR spectra

I. General information:

Unless specifically noted otherwise, all glassware was flame-dried under vacuum (~0.5 Torr) and cooled under an inert atmosphere (N_2 or Ar) before use. Flash column chromatography was performed using 43-60 μm (230-400 mesh) silica gel.

Materials:
Organic solvents and amine bases were purified before use using a Phoenix Solvent Drying System (for organic solvents, available from JC-Meyer Solvent Systems) or PureSolv Micro amine drying columns (for amine bases, available from Innovative Technology/Inert), respectively, and maintained under positive argon pressure. All measurements were performed in anhydrous acetonitrile (MeCN) due to its moderate dielectric constant, inertness towards *N*-hydroxy and *N*-oxyl compounds, and comparatively high solubility of both species relative to other solvents.

Characterization:
Nuclear magnetic resonance (NMR) spectra were obtained using an internal deuterium lock on a Bruker 500 MHz NMR spectrometer. For ^1H spectra, chemical shifts were referenced to the center line of the residual solvent signal ($CDCl_3$: δ 7.26) and are reported in parts per million (ppm). Signal multiplicity is reported as follows: (br = broad, s = singlet, d = doublet, t = triplet, dd = doublet of doublets, m = multiplet), and the associated coupling constants are given in Hertz. For ^{13}C spectra, chemical shifts are reported as ppm using the center line of the solvent signal as reference ($CDCl_3$: δ 77.16).

II. Molecular structures of the training set

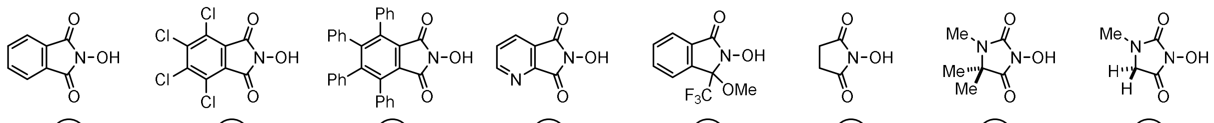

1 2 3 4 5 6 7 8

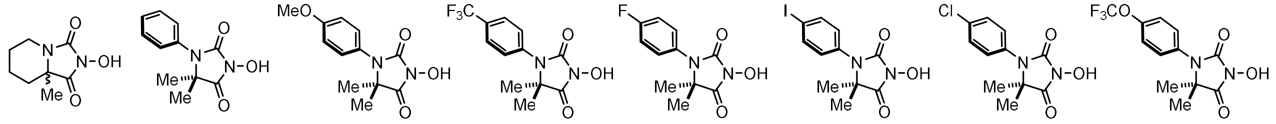

9 10 11 12 13 14 15 16

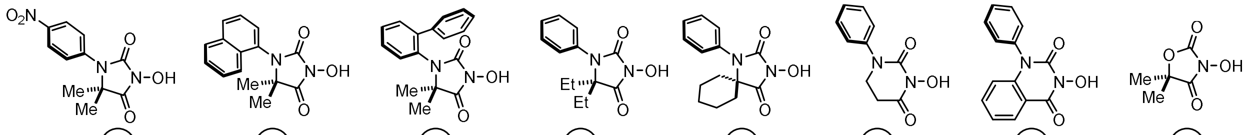

17 18 19 20 21 22 23 24

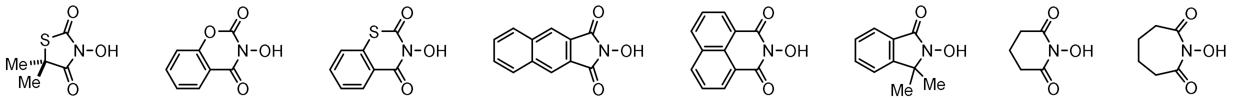

25 26 27 28 29 30 31 32

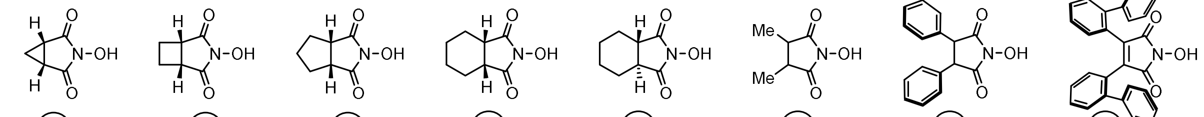

33 34 35 36 37 38 39 40

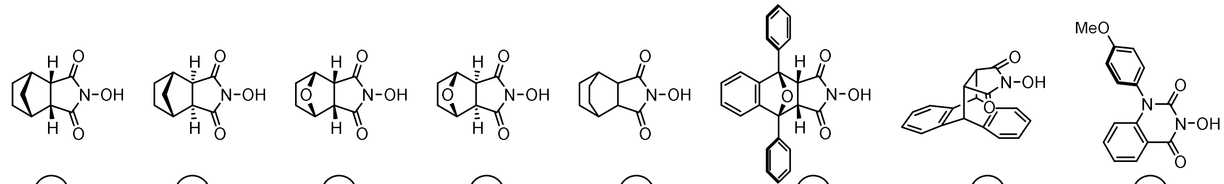

41 42 43 44 45 46 47 48

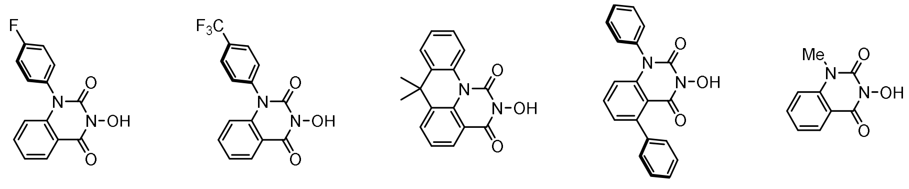

49 50 51 52 53

III. Procedures for evaluating electrocatalysts

1) Cyclic voltammetry of *N*-hydroxyl/*N*-oxyl couple (peak potential E_{pa})

The measurements of the potential for oxidation of *N*-hydroxyl were performed according to a previous publication. [1] Pyridine was used as a base throughout the experiment. Cyclic voltammetry was performed in a three-electrode undivided cell (SVC-3, from BioLogic Science Instruments), using a glassy carbon working disc electrode (S = 0.0707 cm^2 from CH Instruments), a graphite rod counter electrode (d = 0.5 cm from McMaster Carr) and a hand-made silver nitrate/silver ($AgNO_3$/Ag) reference electrode. The solution consists of 2 mM *N*-hydroxyl compounds, 0.2 M pyridine, and 0.1 M tetrabutylammonium hexafluorophosphate (NBu_4PF_6) in acetonitrile. CH Instruments models 620E potentiostat was used throughout. Prior to each set of experiments, a glassy carbon working electrode was polished with 5, 1, and 0.05 μm alumina slurries and dried by blowing nitrogen for each experiment. All reported potentials were calibrated by ferrocenium/ferrocene (Fc^+/Fc) after each experiment. NBu_4PF_6 was purchased from Oakwood Chemical and was recrystallized from ethanol (190 proof). [2]

2) Cyclic voltammetric titration experiments (catalysis kinetics k_{HAT})

The titration experiments were performed according to a previous publication. [1] A solution of 0.1 M NBu_4PF_6 in MeCN was used as a reaction media solution. Catalyst precursor *N*-hydroxy compounds (2 mM) and pyridine (0.2 M) were added to 5 mL of electrolyte solution. Cyclic voltammetry for oxidation of *N*-hydroxy compounds was performed at 0.1 Vs^{-1}. Substrate model 1-phenylethanol (20 mM, 40 mM, 60 mM, 80 mM, 100 mM) was successively titrated to the solution followed by the collection of cyclic voltammetry. All reported potentials were calibrated by ferrocenium/ferrocene (Fc+/Fc) after each experiment.

3) Electrolyses of *N*-hydroxyl compounds (catalyst decomposition kinetics r_{decay})

Electrochemical reactions were acquired using μStat4000 Multi-Channel Potentiostat/Galvanostat (from Metrohm USA). A self-assembled glassy carbon plate electrode (from Goodfellow) was used as an anode and a graphite rod (from McMaster Carr) was used as a cathode. All the reactions were referenced by a hand-made silver nitrate/silver ($AgNO_3$/Ag) reference electrode. For the divided cell experiments, a piece of glass tube with an ultra-fine frit (from ROBU #15105 10mm Diameter, #5 Porosity Fritted Disc) on the bottom was used to separate two electrodes. Electrolyte potassium hexafluorophosphate (KPF_6) was purchased from Acros Organics and recrystallized from water/ethanol (v/v 95:5). [2]

The decomposition of electrochemically-generated *N*-oxyl compounds was monitored using a FlowNMR method according to a previous publication. [3] Alternatively, the spectroscopy data can be collected by taking aliquots every 15 minutes.All the reactions were performed in a divided cell equipped with a glassy carbon plate anode (1 cm x 2 cm), a graphite cathode rod (d = 0.5 cm^2), and an $AgNO_3$/Ag reference electrode. A solution of 0.1 M KPF_6 in MeCN was used as a reaction media solution. In the anodic chamber, *N*-hydroxy compound (0.016 mmol, 10 mmol%) and pyridine (0.32 mmol, 2 equiv.) were added to 8 mL of reaction solution. In the cathodic chamber, a solution of 0.1 M KPF_6 in MeCN was added. Cyclic voltammetry was performed at 0.1 Vs^{-1} to measure anodic peak potential (E_{pa}) of the redox couple prior to electrolysis. Electrolysis was performed at ambient temperature with a constant potential (E_{pa} + 0.2 V). The ^{1}H NMR spectroscopy was recorded throughout the experiments.

IV. Statistical Modeling

Computational Methods

Conformational Searches

Each catalyst was subjected to a molecular mechanics conformational search using MacroModel [4] using the OPLS4 [5] force field. Searches were run in the gas phase with a convergence threshold of .001 and with a maximum of 10,000 iterations. Only conformers within 5.02 kcal/mol of the minimum conformer were collected. If the ensemble had greater than 20 conformers, the conformer ensembles were reduced by atomic root mean square deviation (RMSD)-clustering to the minimum Kelley penalty value [6]. This truncated ensemble was used in place of the full conformer ensemble in these instances. Conformational searches were performed on the ground state, catalyst precursor structures. Conformers for the radical state, active catalyst structures were obtained by deletion of the hydrogen atom of the O–H bond. This technique was chosen to maintain consistency for subsequent bond dissociation energy (BDE) calculations.

DFT Computations

DFT calculations were performed on both the catalyst precursor and catalyst using Gaussian 16 (revision C.01) [7]. Geometry optimization was performed in the gas phase at the M062X/Def2TZVP level of theory with ultrafine grid integration [8,9]. Calculations were performed in the gas phase to simplify and expedite collection of properties. As the property values are being used only as relative comparisons of one catalyst to another rather than as absolute property values, we concluded the approach to be sufficient for our purposes. A frequency calculation was subsequently performed to verify the optimized structures as energetic minima (zero imaginary frequencies). Single point calculations were also performed at the M062X/Def2TZVP level of theory, to obtain properties for NMR shifts and Hirshfeld charges, and natural bond orbital (NBO) analysis was performed using version 7.0 of the NBO program [10]. NMR shielding tensors were calculated using the Gauge-Independent Atomic Orbital (GIAO) method.

Molecular Descriptor Details

General Descriptor Collection

Molecular descriptors were collected from the Gaussian output files (.log) using an in-house Jupyter notebook and python script [11]. Sterimol values were calculated using the Morfeus program [12] and GoodVibes was utilized to obtain energies [13]. Descriptors were collected for the catalyst precursor and catalyst structures. Additional descriptors were computed and considered at early stages of the project. The labeling for all included descriptors is described below.

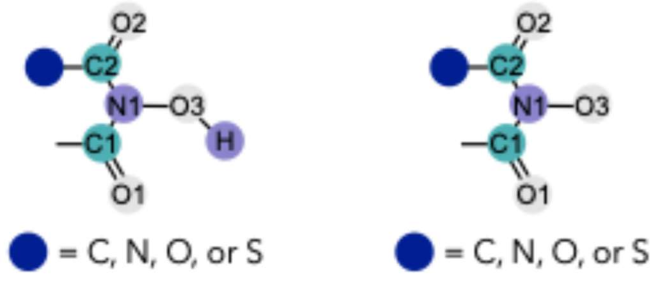

catalyst precursor labeling **catalyst labeling**

Collected Descriptors:

- Frontier Orbitals: E(HOMO), E(LUMO)
- NBO Charge (C1, C2, N1, O1, O2, O3)
- NMR Shift (C1, C2, H (catalyst precursor only))
- Percent Buried Volume (N1, C1, C2) 4.0 Angstroms
- Sterimol B1, L, B5 (C1–N1, C2–N1)
- Hirshfeld Charge and atom dipole moment (C1, C2, O1, O2, N1, O3)
- NBO Bond energy and occupancy (C1–N1, C2–N1)
- BDE O–H bond (catalyst precursor only)

Bond Dissociation Free Energy Calculation

Bond dissociation free energy (BDFE) was calculated for the catalysts using a separate python script. BDFE was calculated only for the lowest energy conformer of the ground state conformer ensemble. The radical catalyst used as the produce for these calculations was the exact conformer used as the lowest energy conformer for the ground state. The hydrogen was deleted, and the conformer was DFT-optimized. The other product, the hydrogen atom radical, was calculated at the same level of theory as described above. The BDFE was calculated as: $BDFE = (G_{catalyst} + G_{H\ radical}) - G_{catalyst\ precursor}$

Modeling Details

Model Search and Selection for k_{HAT}, E_{pa}

Experimental data for 50 catalyst structures were utilized for construction of rate of catalysis and oxidation peak potential models. Structure 5, 30, 54 and 55 were not included in modeling as they did not include the substructure used to parameterize the catalysts. Structure 37 was excluded from modeling because exact rate of catalysis and oxidation peak potential data could not be obtained. Descriptors for both the catalyst precursor and catalyst were included for each of the 50 structures. The data was divided into a training (70%) and test set (30%) using either the Kennard-Stone algorithm or to achieve an equidistance coverage of experimental output [14]. All parameters were then scaled using the Standard Scaler from the publicly available python package scikit learn. Linear models were identified using previously reported forward stepwise linear regression algorithms [15]. In this search, parameters with a collinearity greater than $R^2 = 0.5$ were prohibited from inclusion in a single model. If two features were excluded from a single model due to high collinearity, models using each respective feature were evaluated. Linear models were evaluated based on training set R^2, test set R^2, Q^2 and mean absolute error (MAE) for the training and test set. The selection of the final model was primarily based on the interpretability of parameters and high statistical performance. Models were first downselected to those with desirable performance (typically training and test $R^2 > 0.80$). From these models, the expert chemist selected those which were most interpretable. Although there may be multiple models that meet all these requirements, many of these models are found to redundant or non-unique. As an example, redundant is defined as a 3 term model that contains descriptors that are collinear ($R^2 > 0.7$) with the 3 terms in another model. This largely occurs with various condensed descriptor values. In selecting the most interpretable model, these redundant models are typically removed as they offer the same chemical insight as one another. Furthermore, models which contained only a single form of condensed descriptor (ex. only lowest energy conformer descriptors) were favored over those with multiple forms of condensed descriptors. An example of this filtering process may be found in an attached document "analysis all epa models.xlsx". The first sheet "all_possible_epa_models" shows an example of what the initial output may be. The next sheet "epa_models_colin_cut" shows the removing of redundant and low performing models. Before selecting a final model, we also check that parameters in the model always have the same direction of impact on prediction outcome

to ensure they are not just statistically but chemically meaningful (sheet_name = “feat_appearance_not_condensed”). The model presented in the paper and an additional sampling of acceptable linear models are shown below. Both split methods and the resulting changes to model statistics are shown in Table S1 and S2. Methods beyond MLR were also investigated; however, if an appropriate MLR model was found to sufficiently explain the data this model was prioritized due to the interpretability of these simple models. Statistics and notes on other models are shown below in Table S5.

Table SI. Rate Models

<table>
<tr><td>Y-equidistant Split</td><td>Kennard Stone Split</td></tr>
<tr><td>
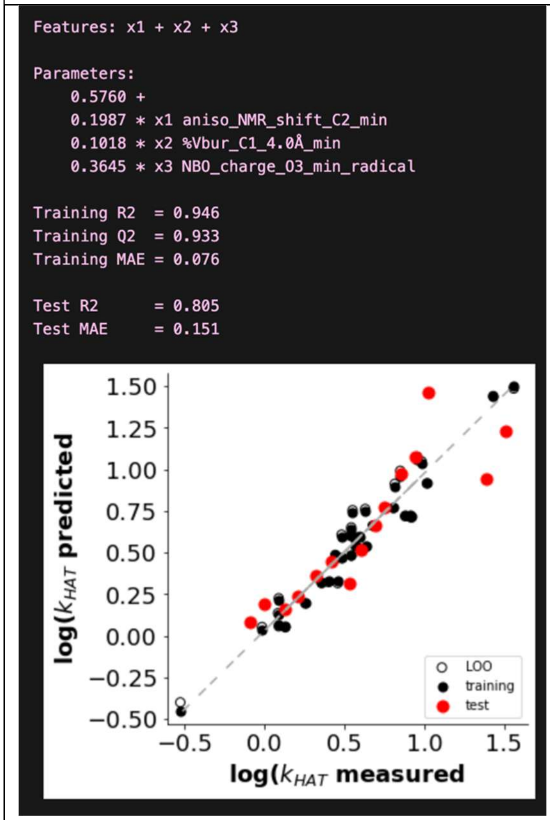

</td><td>
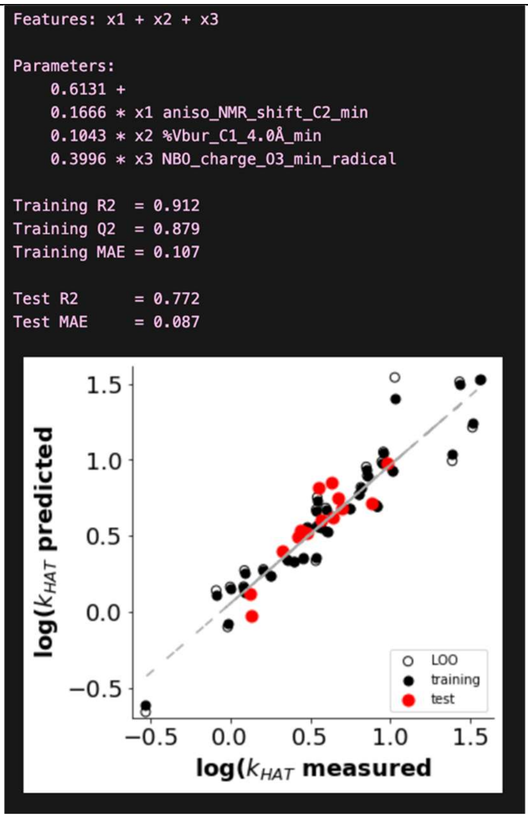

</td></tr>
</table>

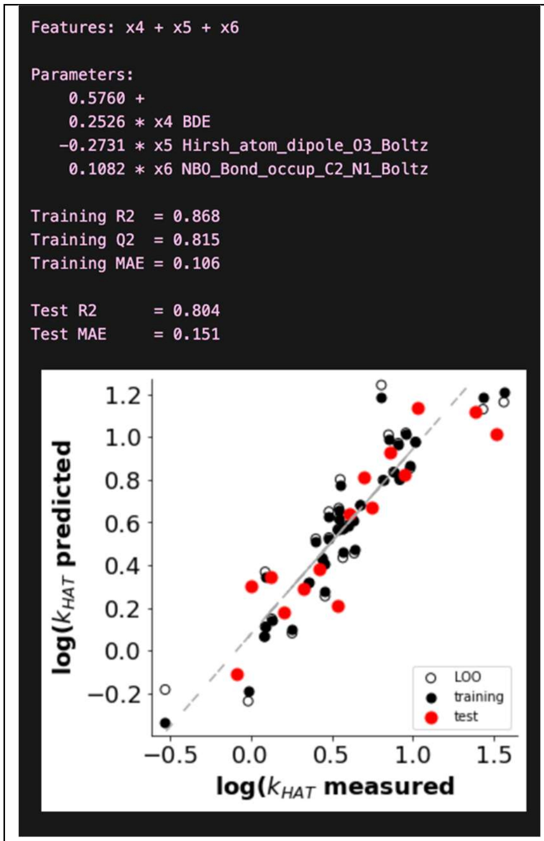

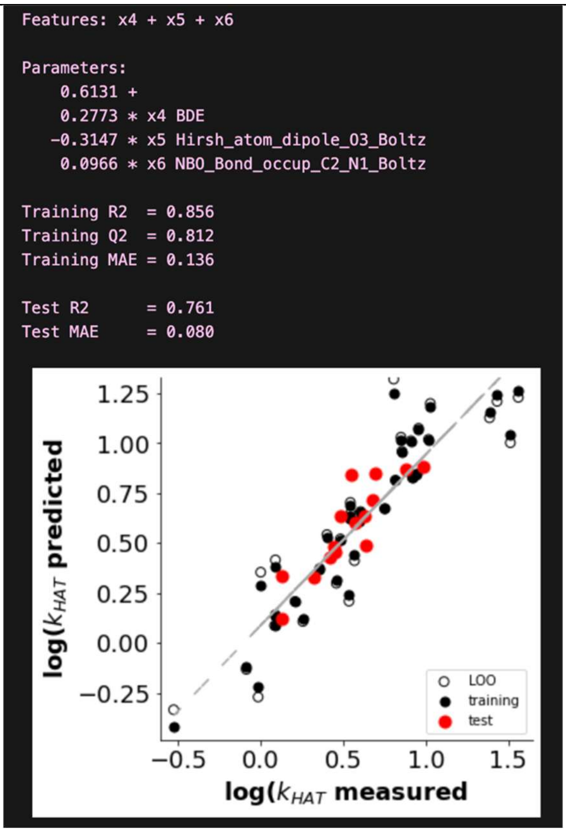

Table S2. Peak Oxidation Potential Models

Y-equidistant Split	Kennard Stone Split

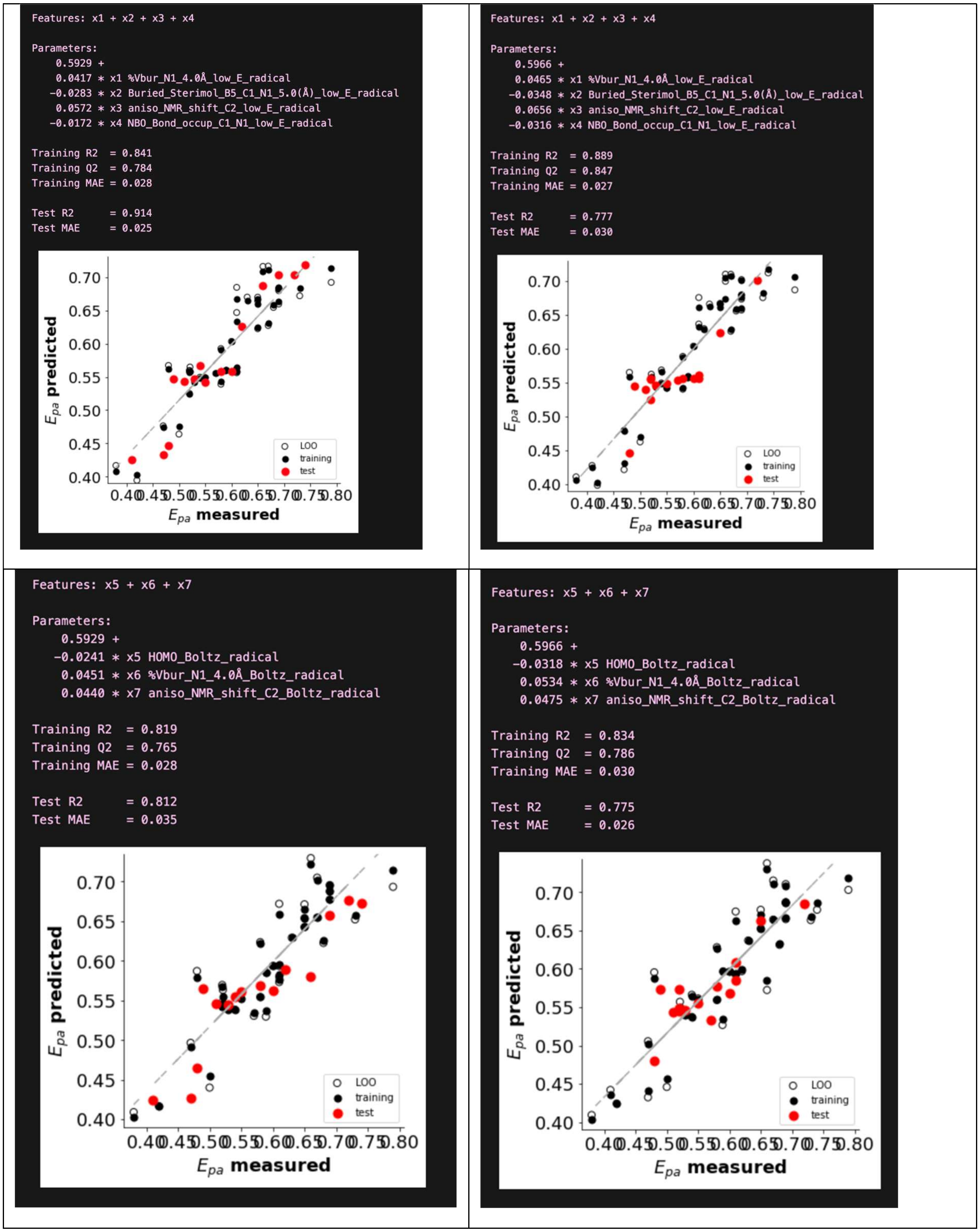

Model Search and Selection for r_{decay}

A search for multivariable linear regression (MLR) and single node decision tree models for the full 50 catalyst data set was performed; however, this did not yield statistically desirable models. Upon recognition of the multiple possible mechanisms of decay, two subsets of the data were determined based on substructure and studied

decomposition of these structure types. The first category (structures 7-21) contained NHS-derived catalysts while the second category (structure 6, 33-47) contained NHPI type catalysts. Modeling of the first category excluded structures 15 and 16 as no data was available for rate of decay. An MLR model was located following the procedure described above for rate of catalysis and peak oxidation potential models. Alternative models and alternative splits of the selected model are shown below in **Table S4**. In modeling the second category, structure 45 was excluded because no data was available for rate of decay. A single parameter classification was identified, alternative classification are shown below in **Table S3**. All classifications shown are based off a user-defined threshold describing $r_{decay} < 1.5$ as desirable and $r_{decay} > 1.5$ as undesirable. Due to the size of these split datasets, the type of modeling which could be applied was severely limited; therefore, primarily MLR and single node decision trees were investigated for these datasets.

Table S3. Alternative Models for Rate of Decay (Bimolecular Decay)

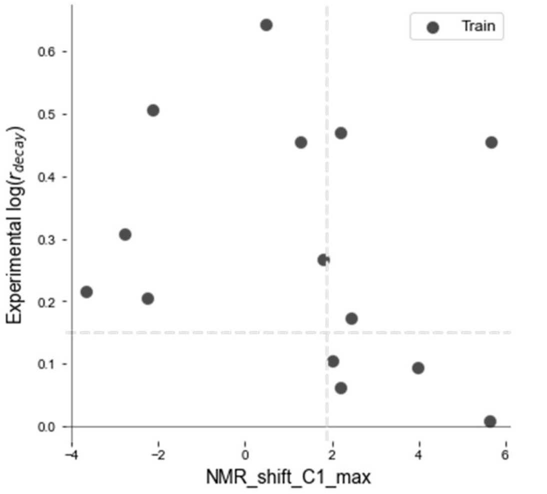

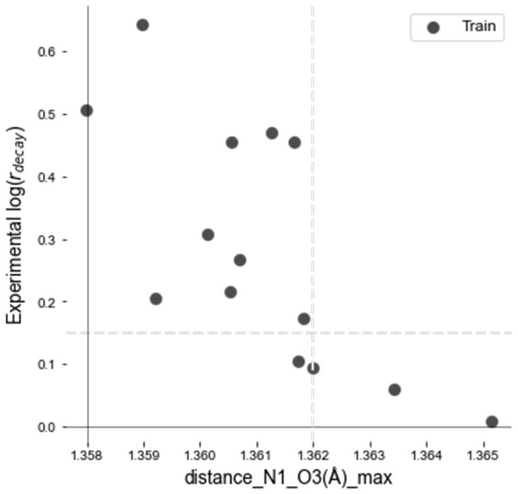

Table S4. Alternative Models for Rate of Decay (Base-Promoted)

<table>
<tr><th>Y-equidistant Split</th><th>Kennard Stone Split</th></tr>
<tr><td>
Parameters:
-0.3171 +
-0.4436 * x209 %Vbur_O2_2.5Å_Boltz
-0.4135 * x529 dipole(Debye)_Boltz_radical

Training R2 = 0.933
Training Q2 = 0.874
Training MAE = 0.101
Training K-fold R2 = 0.867 (+/- 0.001)

Test R2 = 0.935
Test MAE = 0.093
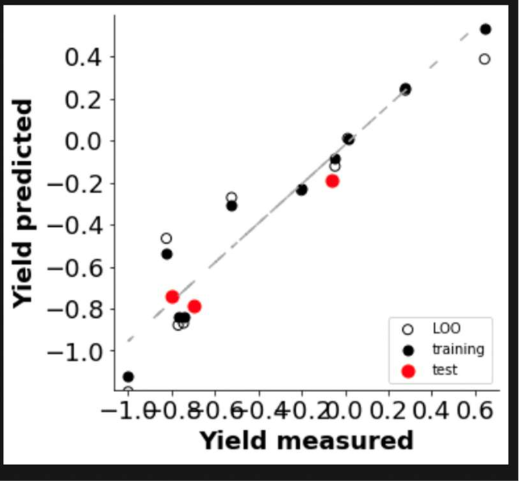

</td><td>
Parameters:
-0.2523 +
-0.4796 * x209 %Vbur_O2_2.5Å_Boltz
-0.3856 * x529 dipole(Debye)_Boltz_radical

Training R2 = 0.928
Training Q2 = 0.846
Training MAE = 0.099
Training K-fold R2 = 0.834 (+/- 0.001)

Test R2 = 0.902
Test MAE = 0.140
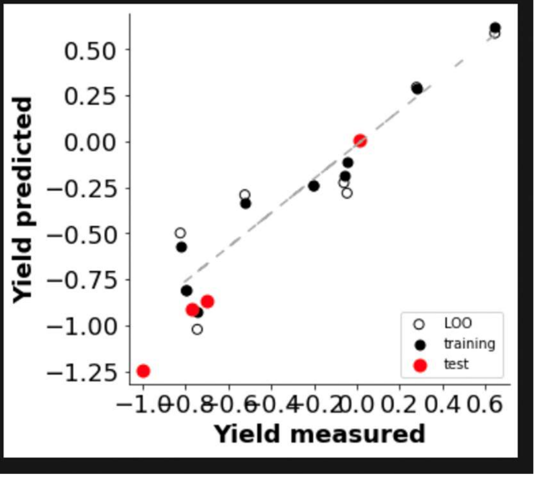

</td></tr>
</table>

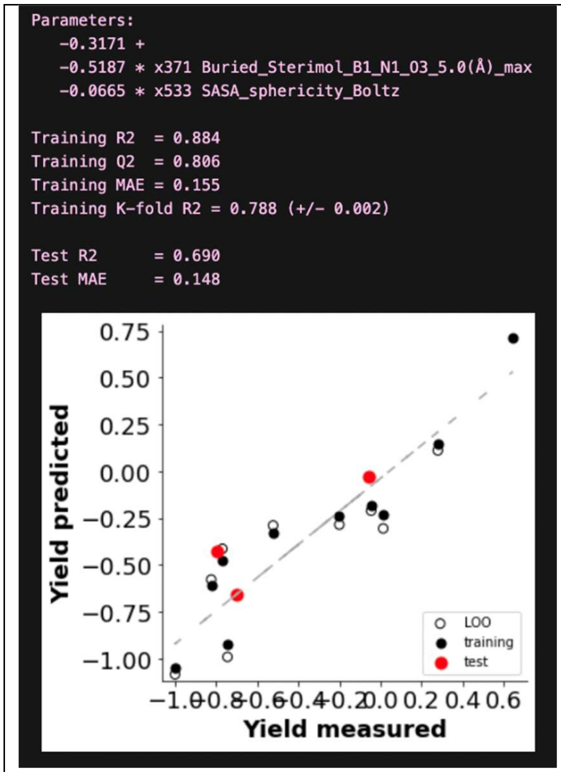

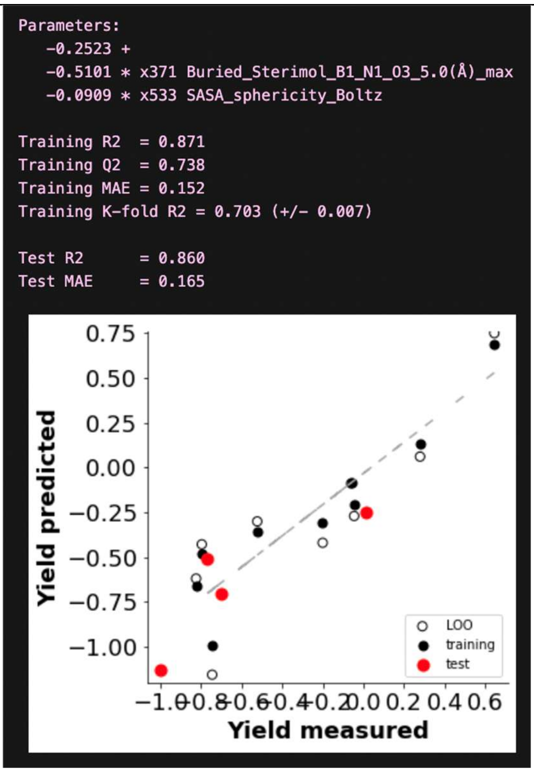

Table S5. Results for Non-Linear Modeling Efforts

model_type	reaction_outcome	features	r2	MAE	hyperparameters	scaler	notes
svm regression	khat	na	0.05	0.28	kernel= rbf, C =0.1, epsilon=0.01	standard	poor performance
svm regression	khat	na	-5.89	0.59	kernel= poly, C =0.1, epsilon=0.01	standard	poor performance
svm regression	khat	na	-0.34	0.3	kernel= linear, C =0.1, epsilon=0.01	standard	poor performance
svm regression	epa	na	0.27	0.05	kernel= poly, C =0.1, epsilon=0.01	standard	poor performance
svm regression	epa	na	0.13	0.06	kernel= rbf, C =0.1, epsilon=0.01	standard	poor performance
svm regression	epa	na	-0.55	0.06	kernel= linear, C =0.1, epsilon=0.01	standard	poor performance

symbolic regression	khat	abs(abs(cos(%Vbur_N1_3.5Å_Boltz_radical)))	0.38	-0.67	na	standard	poor performance - all other variations extremely overfit

model_type	reaction_outcome	y_cut	features	accuracy	recall	precision	f1	hyperparameters	notes
decision tree classifier	khat	khat >/< 4.0	NBO Bond occupancy C2_B1 Boltzmann < 1.986, Hirsh CM5 charge O3 max radical < -0.10	0.87	0.9	0.88	0.87	max_depth = 5, criterion= entropy, min_samples_split=3	performance fine, regression equivalent and preferred
decision tree classifier	epa	epa >/< 0.5	Vbur N1 (2.5 A) max radical < 84.19	0.87	0.89	0.8	0.83	max_depth = 5, criterion= entropy, min_samples_split=3	performance fine, regression equivalent and preferred
decision tree classifier	rate - structures 7-21	kdecay >/< 1.0	Sterimol_B1_N1_O3 low E < 2.801	0.5	0.33	0.5	0.33	max_depth = 5, criterion= entropy, min_samples_split=3	poor performing model, not used
decision tree classifier	rate - structures 6,33-47	kdecay >/< 1.5	Sterimol L, N1_O3 max < 3.282	1	1	1	1	max_depth = 5, criterion= entropy, min_samples_split=3	model shown in paper

V. Virtual Screening Catalysts

Computational Methods and Descriptor Collection

General Procedure

Catalysts in the virtual library involve further modifications of existing catalyst structures which represent a further diversified and, ideally, synthetically accessible collection of structures. Virtual screening catalysts underwent the same computational workflow described above for the original catalyst library. Atom numbering is consistent with that described above.

Selection of Catalysts for Synthesis

Selection of Diverse Catalysts for Synthesis

Catalysts were selected based upon the predicted experimental outcome for the virtual catalyst. For each regression model, the parameters and coefficients used in the experimental model were applied to get a prediction for the experimental outcome of the virtual catalyst. The virtual catalyst descriptors were then tested in the model to get a predicted outcome for the experimental objective. For the classification model, the catalysts were predicted as "on" or "off" based on the threshold value for the relevant descriptor. A (No.33) , B (No.44), and C (No.47) were selected solely because they were predicted to yield desirable values for the rate of catalysis. Structures D (No.47) and E (No.101) were selected to balance all three experimental objectives. Finally, structure F (No.2) was selected to give both a desirable peak oxidation potential and rate of decay. All final structure selections were also dependent on the synthetic accessibility of the catalyst.

VII. Molecular structures of the virtual screening library

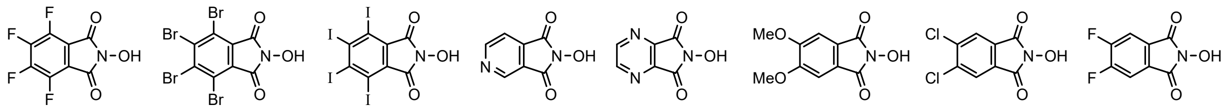

1 2 3 4 5 6 7 8

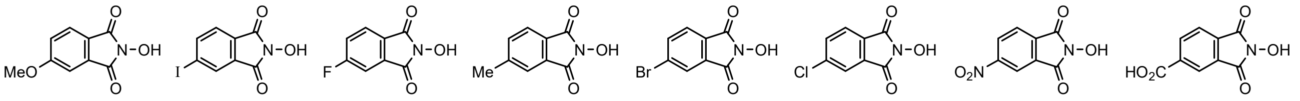

9 10 11 12 13 14 15 16

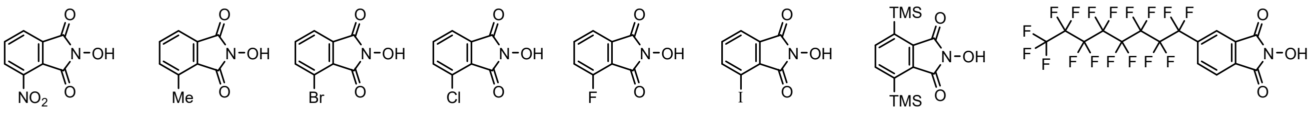

17 18 19 20 21 22 23 24

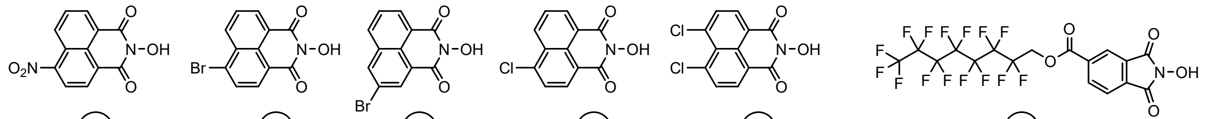

25 26 27 28 29 30

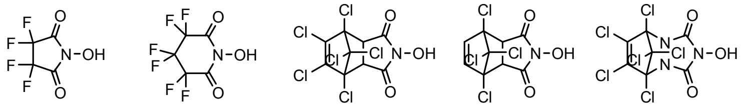

31 32 33 34 35

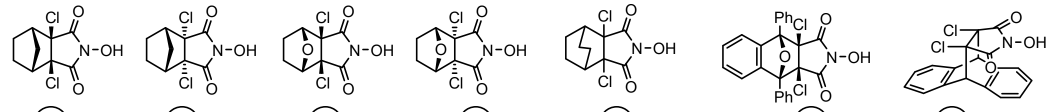

36 37 38 39 40 41 42

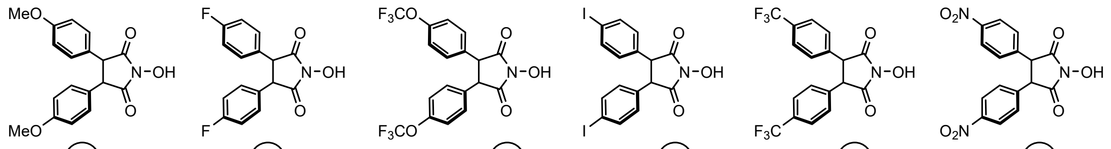

43 44 45 46 47 48

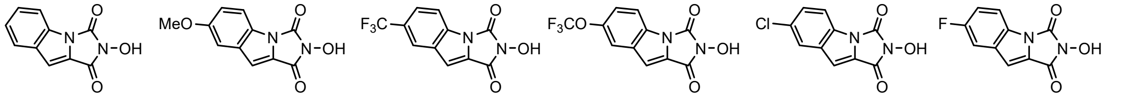

49 50 51 52 53 54

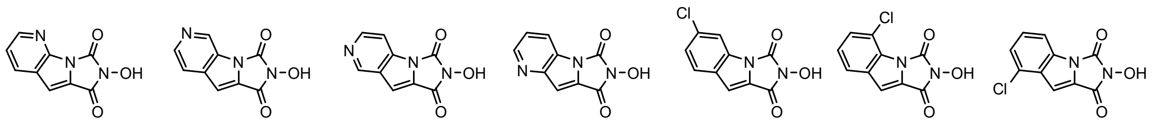

55 56 57 58 59 60 61

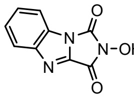

62

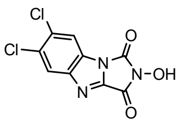

63

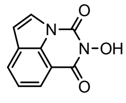

64

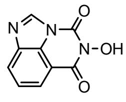

65

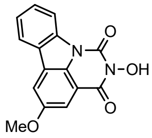

66

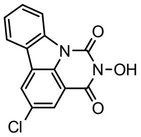

67

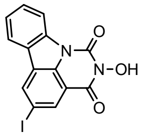

68

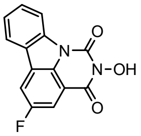

69

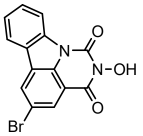

70

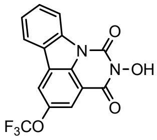

71

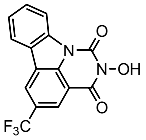

72

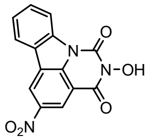

73

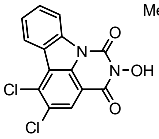

74

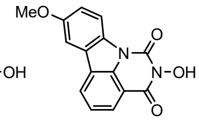

75

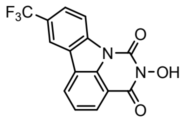

76

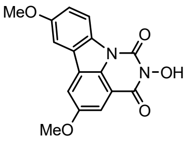

77

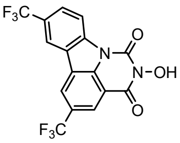

78

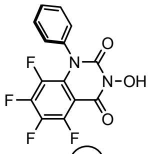

79

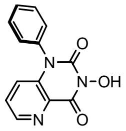

80

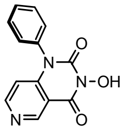

81

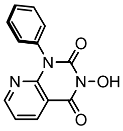

82

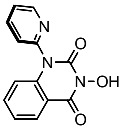

83

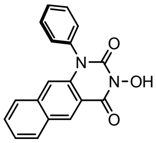

84

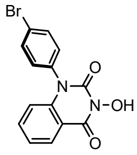

85

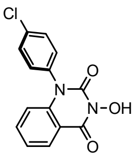

86

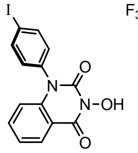

87

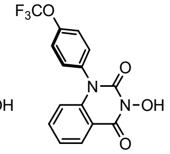

88

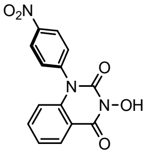

89

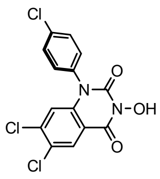

90

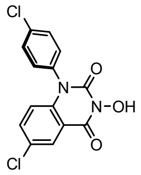

91

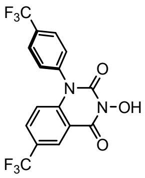

92

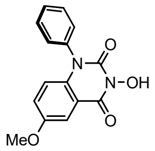

93

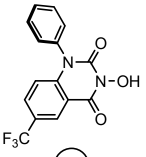

94

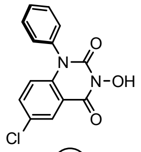

95

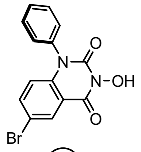

96

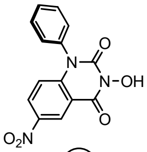

97

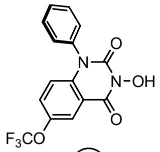

98

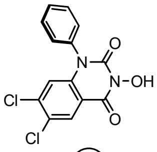

99

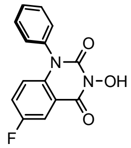

100

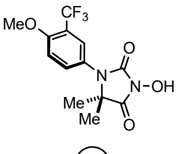

101

VII. Synthesis of *N*-hydroxy compounds

In the training set, compounds 1, 2, 4, 6, 28, 29, and 31 were purchased and used without further purification. Compound 3 was synthesized according to ref. [16]. Compound 5 was synthesized according to ref. [17]. Compounds 7-21 were synthesized according to our previous publication ref [3]. Compounds 22-27, and 51-53 were synthesized according to our previous publication ref [18]. Compound 30 was synthesized according to ref [19]. Compound 32 was synthesized according to ref [20]. Compound 35 was synthesized according to ref [21]. Compound 37 was synthesized according to ref [22]. Compound 39 was synthesized according to ref [23]. Compound 40 was synthesized according to ref [24]. Compounds 43 and 44 were synthesized according to ref [25]. Compound A was synthesized according to ref [1]. Compounds B and C were synthesized according to ref [24]. Compound D was synthesized according to ref [26]. Compound F was synthesized according to ref [16].

The synthesis of all other compounds is listed below.

General procedure A.

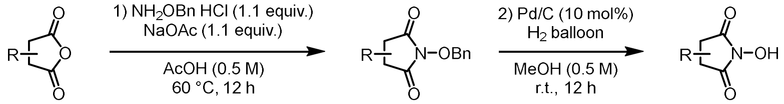

1) To a pressure tube was added anhydride (10 mmol, 1 equiv.), *O*-benzylhydroxylamine hydrogen chloride (1.1 equiv.), sodium acetate (1.1 equiv.). and acetic acid (0.2 M). The solution was stirred at 65 ℃ for 12 hours. After cooling to room temperature, water was added to allow precipitation. The resulting solid was collected by vacuum filtration and subjected to the next synthetic step without purification.

2) To a flask, benzyl-protected hydantoin (1 equiv.) was dissolved in MeOH (0.5 M). The solution was sparged with argon for 15 min followed by the addition of Pd/C (10 mol%). The resulting mixture was stirred at ambient temperature under hydrogen for 12 h. After the consumption of the starting material, Pd/C was removed by vacuum filtration. Evaporation of organic solvent gave crude which was purified over flash column chromatography (EtOAc/Hexanes) to afford the desired product.

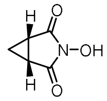

(1R,5S)-3-hydroxy-3-azabicyclo[3.1.0]hexane-2,4-dione (90% yield)

^{1}H NMR (500 MHz, $CDCl_3$) δ 2.47 (dd, J = 7.9, 3.8 Hz, 2H), 1.70 – 1.66 (m, 2H), 1.66 – 1.62 (m, 2H), 1.62 – 1.59 (m, 1H).

^{13}C NMR (126 MHz, $CDCl_3$) δ 170.4, 77.3, 76.8, 21.6, 16.9.

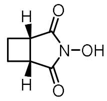

(1R,5S)-3-hydroxy-3-azabicyclo[3.2.0]heptane-2,4-dione (92% yield)

^{1}H NMR (500 MHz, $CDCl_3$) δ 7.84 (br s, 1H), 3.39 – 3.31 (m, 2H), 2.76 – 2.63 (m, 2H), 2.30 – 2.17 (m, 2H).

^{13}C NMR (126 MHz, $CDCl_3$) δ 174.2, 133.4, 130.4, 129.7, 128.7, 78.4, 35.6, 22.9.

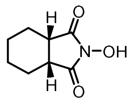

(3aR,7aS)-2-hydroxyhexahydro-1H-isoindole-1,3(2H)-dione (97% yield)

^{1}H NMR (500 MHz, $CDCl_3$) δ 8.35 (br s, 1H), 2.91 (td, *J* = 4.5, 2.2 Hz, 2H), 1.94 – 1.86 (m, 2H), 1.82 – 1.77 (m, 2H), 1.54 – 1.40 (m, 4H).

^{13}C NMR (126 MHz, $CDCl_3$) δ 175.9, 37.3, 23.5, 21.2.

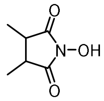

1-hydroxy-3,4-dimethylpyrrolidine-2,5-dione (90% yield)

^{1}H NMR (500 MHz, $CDCl_3$) δ 8.59 (br s, 1H), 3.03 – 2.93 (m, 2H), 1.28 – 1.20 (m, 6H).

^{13}C NMR (126 MHz, $CDCl_3$) δ 176.5, 35.7, 11.3.

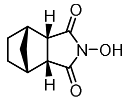

(3aR,4R,7S,7aS)-2-hydroxyhexahydro-1H-4,7-methanoisoindole-1,3(2H)-dione (98% yield)

^{1}H NMR (500 MHz, $CDCl_3$) δ 8.14 (br s, 1H), 3.06 (dt, *J* = 2.9, 1.3 Hz, 2H), 2.79 (tp, *J* = 3.6, 1.8 Hz, 2H), 1.71 – 1.59 (m, 4H), 1.36 – 1.24 (m, 2H).

^{13}C NMR (126 MHz, $CDCl_3$) δ 174.4, 45.8, 41.6, 39.0, 24.7.

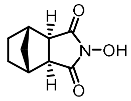

(3aR,4S,7R,7aS)-2-hydroxyhexahydro-1H-4,7-methanoisoindole-1,3(2H)-dione (98% yield)

^{1}H NMR (500 MHz, $CDCl_3$) δ 8.01(br s, 8.01), 2.72 (dq, *J* = 3.2, 1.7 Hz, 2H), 2.64 (d, *J* = 1.3 Hz, 2H), 1.74 – 1.69 (m, 2H), 1.37 (td, *J* = 7.9, 7.2, 2.3 Hz, 2H), 1.28 (dt, *J* = 11.3, 1.5 Hz, 1H), 1.21 (dt, *J* = 11.3, 2.0 Hz, 1H).

^{13}C NMR (126 MHz, $CDCl_3$) δ 174.5, 46.1, 39.5, 3.3, 28.2.

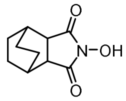

2-hydroxyhexahydro-1H-4,7-ethanoisoindole-1,3(2H)-dione (96% yield)

^{1}H NMR (500 MHz, $CDCl_3$) δ 8.10 (s, 1H), 2.85 (t, *J* = 1.7 Hz, 2H), 2.20 (dt, *J* = 3.6, 1.8 Hz, 2H), 1.77 – 1.69 (m, 2H), 1.48 (s, 4H).

^{13}C NMR (126 MHz, $CDCl_3$) δ 175.3, 41.3, 26.2, 24.9, 21.3.

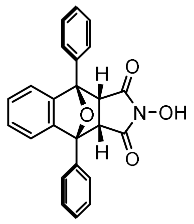

(3aR,4R,9S,9aS)-2-hydroxy-4,9-diphenyl-3a,4,9,9a-tetrahydro-1H-4,9-epoxybenzo[f]isoindole-1,3(2H)-dione (95% yield)

^{1}H NMR (500 MHz, $CDCl_3$) δ 7.78 – 7.72 (m, 4H), 7.54 (dd, *J* = 8.4, 6.9 Hz, 4H), 7.49 – 7.42 (m, 2H), 7.24 – 7.18 (m, 4H), 6.69 (br s, 1H), 3.53 (s, 2H).

^{13}C NMR (126 MHz, $CDCl_3$) δ 167.3, 146.1, 133.6, 128.7, 128.6, 128.2, 126.3, 120.0, 90.5, 51.2.

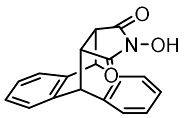

(9R,10S,11R,15S)-13-hydroxy-9,10-dihydro-9,10-[3,4]epipyrroloanthracene-12,14-dione (97% yield)

^{1}H NMR (500 MHz, $CDCl_3$) δ 7.38 (dd, *J* = 5.4, 3.2 Hz, 2H), 7.30 (dd, *J* = 5.4, 3.3 Hz, 2H), 7.18 (ddd, *J* = 15.9, 5.5, 3.2 Hz, 4H), 4.81 (t, *J* = 1.7 Hz, 2H), 3.19 (t, *J* = 1.6 Hz, 2H).

^{13}C NMR (126 MHz, $cdcl_3$ + dmso-d_6) δ 170.7, 140.2, 137.6, 125.6, 125.3, 123.6, 122.9, 43.6, 43.5, 42.3.

General procedure B.

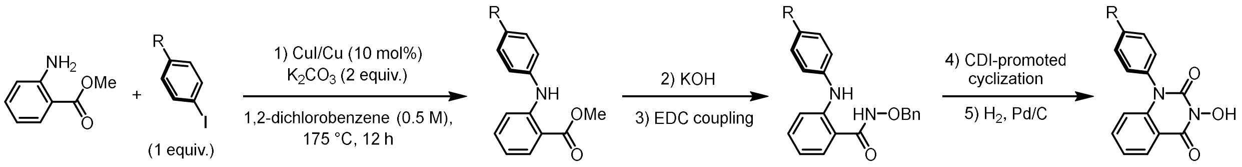

1) To a solution of methyl anthranilate (20 mmol, 1 equiv.) and aryl iodide (1 equiv.) in 1,2-dichlorobenzene (0.5 M), catalysts CuI/Cu (10 mmol%) and base K_2CO_3 (2 equiv.) were added. The resulting suspension was stirred at 175 °C in a pressure tube for 12 h. After cooling to room temperature, water (10 times volume) was added. The aqueous solution was extracted by EtOAc (x 3). The combined organic layers were dried over Na_2SO_4. The solvent was removed under reduced pressure. The resulting crude was purified over flash column chromatography (EtOAc/Hexanes) to afford the desired product.

2) To a solution of ester (10 mmol, 1 equiv.) in MeOH/H_2O (0.5 M, v/v = 3:2), KOH (2 equiv.) was added. The reaction was refluxed for 12 h. After cooling to room temperature, MeOH was removed under reduced pressure before 1 M HCl aqueous solution was added to form precipitation. The solid was collected by vacuum filtration and washed with water. The crude product was dried over vacuum and subjected to the next synthetic step without purification.

3) To a solution of benzoic acid (10 mmol, 1 equiv.) in DMF (0.5 M), EDC HCl (1.5 equiv.), HOBt H_2O (1.5 equiv.), NEt_3 (2 equiv.), and NH_2OBn (1.3 equiv.) were added. The resulting mixture was stirred at room temperature for 12 h before water (10 times volume) was added. The aqueous solution was extracted by EtOAc (x 3). The combined organic layers were washed by 5% LiCl solution and dried over Na_2SO_4. The solvent was removed under reduced pressure. The resulting crude was purified over flash column chromatography (EtOAc/Hexanes) to afford the desired product.

4) To a solution of the above-mentioned EDC coupling product (1 equiv.) in MeCN (0.3 M), CDI (1 equiv.) and *i*Pr_2NEt (2 equiv.) were added. The reaction mixture was then stirred at reflux for 12 h. After cooling to room temperature, the solvent was removed under reduced pressure. The resulting crude was purified over flash column chromatography (EtOAc/Hexanes) to afford the desired product.

5) To a flask, benzyl-protected benzouracil (1.0 equiv.) was dissolved in MeOH (0.2 M). The solution was sparged with argon for 15 min followed by the addition of Pd/C (10 mol%). The resulting mixture was stirred at ambient

temperature under hydrogen for 6 h. After the consumption of the starting material, Pd/C was removed by vacuum filtration. Evaporation of organic solvent gave crude which was purified over flash column chromatography (EtOAc/Hexanes) to afford the desired product.

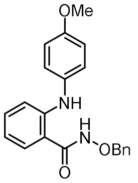

N-(benzyloxy)-2-((4-methoxyphenyl)amino)benzamide (94% yield)

^{1}H NMR (500 MHz, cdcl$_3$) δ 8.69 (s, 1H), 7.45 (d, *J* = 7.1 Hz, 2H), 7.37 (p, *J* = 6.2 Hz, 4H), 7.28 (d, *J* = 8.1 Hz, 1H), 7.21 (t, *J* = 7.9 Hz, 1H), 7.13 (d, *J* = 8.3 Hz, 2H), 7.05 (d, *J* = 8.5 Hz, 1H), 6.88 (d, *J* = 9.1 Hz, 2H), 6.62 (t, *J* = 7.5 Hz, 1H), 5.02 (s, 2H), 3.81 (s, 3H).

^{13}C NMR (126 MHz, cdcl$_3$) δ 156.3, 147.3, 135.4, 133.8, 132.9, 129.3, 128.8, 128.7, 127.4, 124.8, 117.0, 114.7, 114.5, 113.6, 78.4, 55.5.

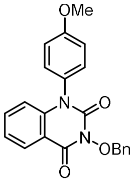

3-(benzyloxy)-1-(4-methoxyphenyl)quinazoline-2,4(1H,3H)-dione (86% yield)

^{1}H NMR (500 MHz, cdcl$_3$) δ 8.27 (dd, *J* = 7.8, 1.6 Hz, 1H), 7.66 – 7.60 (m, 2H), 7.48 (t, *J* = 7.8 Hz, 1H), 7.37 (d, *J* = 5.0 Hz, 3H), 7.27 – 7.22 (m, 3H), 7.10 (d, *J* = 8.8 Hz, 2H), 6.63 (d, *J* = 8.4 Hz, 1H), 5.26 (s, 2H), 3.89 (s, 3H).

^{13}C NMR (126 MHz, cdcl$_3$) δ 160.2, 158.9, 149.1, 141.2, 134.8, 133.8, 130.1, 130.0, 129.1, 128.6, 128.4, 128.2, 123.5, 115.8, 115.6, 115.3, 78.5, 55.6.

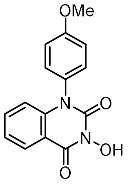

3-hydroxy-1-(4-methoxyphenyl)quinazoline-2,4(1H,3H)-dione (95% yield)

^{1}H NMR (500 MHz, acetone) δ 9.64 (s, 1H), 8.17 – 8.12 (m, 1H), 7.58 (t, *J* = 7.9 Hz, 1H), 7.42 – 7.37 (m, 2H), 7.30 (t, *J* = 7.5 Hz, 1H), 7.16 (d, *J* = 8.7 Hz, 2H), 6.62 (d, *J* = 8.5 Hz, 1H), 3.91 (s, 3H).

^{13}C NMR (126 MHz, acetone) δ 160.2, 158.1, 148.6, 141.3, 134.3, 130.5, 129.1, 127.5, 122.8, 115.6, 115.2, 115.1, 55.1, 55.0.

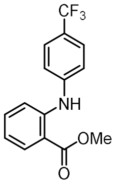

methyl 2-((4-(trifluoromethyl)phenyl)amino)benzoate (80% yield)

^{1}H NMR (700 MHz, cdcl$_3$) δ 9.64 (br s, 1H), 8.00 (d, *J* = 8.0 Hz, 1H), 7.56 (d, *J* = 8.1 Hz, 2H), 7.40 (s, 2H), 7.30 (d, *J* = 8.3 Hz, 2H), 6.86 (t, *J* = 7.6 Hz, 1H), 3.92 (s, 3H).

^{13}C NMR (126 MHz, CDCl$_3$) δ 168.9, 146.1, 144.6, 134.3, 131.9, 127.8, 126.8, 126.8, 126.78, 126.7, 125.6, 124.70, 124.4, 124.2, 123.9, 123.5, 121.3, 120.1, 118.9, 115.3, 113.8, 52.2.

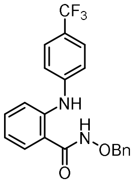

N-(benzyloxy)-2-((4-(trifluoromethyl)phenyl)amino)benzamide (90% yield)

^{1}H NMR (700 MHz, cdcl$_3$) δ 9.10 (br s, 1H) 8.42 (br s, 1H), 7.52 (d, *J* = 8.4 Hz, 2H), 7.44 (d, *J* = 7.2 Hz, 3H), 7.42 – 7.33 (m, 5H), 7.21 (d, *J* = 8.8 Hz, 2H), 6.84 (s, 1H), 5.04 (s, 2H).

^{13}C NMR (126 MHz, CDCl$_3$) δ 168.2, 144.9, 143.8, 135.2, 133.0, 129.5, 129.1, 128.9, 127.8, 126.8, 126.8, 126.8, 126.7, 125.7, 123.9, 123.7, 123.5, 123.4, 123.1, 121.4, 119.9, 118.6, 117.2, 78.7.

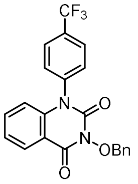

3-(benzyloxy)-1-(4-(trifluoromethyl)phenyl)quinazoline-2,4(1H,3H)-dione (86% yield)

^{1}H NMR (700 MHz, cdcl$_3$) δ 8.31 (d, *J* = 7.9 Hz, 1H), 7.89 (d, *J* = 8.0 Hz, 2H), 7.64 – 7.60 (m, 2H), 7.50 (t, *J* = 8.9 Hz, 3H), 7.38 (d, *J* = 5.8 Hz, 3H), 7.31 (t, *J* = 8.0 Hz, 1H), 6.54 (d, *J* = 8.4 Hz, 1H), 5.26 (s, 2H).

^{13}C NMR (126 MHz, CDCl$_3$) δ 158.8, 148.8, 140.3, 139.1, 135.3, 133.8, 132.3, 132.0, 130.3, 130.1, 129.43, 129.1, 128.7, 127.9, 127.9, 127.8, 127.8, 124.8, 124.2, 122.6, 115.7, 115.5, 78.9.

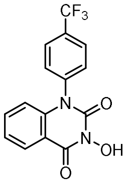

3-hydroxy-1-(4-(trifluoromethyl)phenyl)quinazoline-2,4(1H,3H)-dione (96% yield)

^{1}H NMR (500 MHz, acetone) δ 9.81 (s, 1H), 8.17 (d, *J* = 7.9 Hz, 1H), 8.02 (d, *J* = 8.1 Hz, 2H), 7.79 (d, *J* = 8.2 Hz, 2H), 7.60 (t, *J* = 7.9 Hz, 1H), 7.34 (t, *J* = 7.6 Hz, 1H), 6.60 (d, *J* = 8.5 Hz, 1H).

^{13}C NMR (126 MHz, acetone) δ 158.2, 148.3, 140.4, 136.9, 134.5, 130.7, 127.7, 127.3, 127.3, 125.2, 123.2, 115.3.

^{19}F NMR (471 MHz, acetone) δ -63.1.

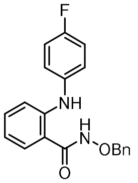

N-(benzyloxy)-2-((4-fluorophenyl)amino)benzamide (84% yield)

^{1}H NMR (700 MHz, cdcl$_3$) δ 8.89 (br s, 1H), 8.42 (br s, 1H), 7.46 (d, *J* = 7.2 Hz, 2H), 7.40 (q, *J* = 9.1, 8.1 Hz, 3H), 7.24 (d, *J* = 8.0 Hz, 1H), 7.14 (q, *J* = 9.1, 7.9 Hz, 3H), 7.02 (t, *J* = 8.3 Hz, 2H), 6.69 (t, *J* = 7.6 Hz, 1H), 5.04 (s, 2H).

^{13}C NMR (126 MHz, CDCl$_3$) δ 160.2, 158.3, 146.7, 137.2, 137.2, 135.5, 133.1, 129.5, 129.0, 128.9, 127.6, 124.1, 124.0, 117.7, 116.3, 116.1, 114.8, 78.6.

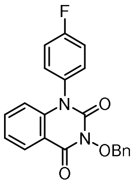

3-(benzyloxy)-1-(4-fluorophenyl)quinazoline-2,4(1H,3H)-dione (89% yield)

^{1}H NMR (500 MHz, cdcl$_3$) δ 8.29 (dd, *J* = 7.9, 1.6 Hz, 1H), 7.65 – 7.59 (m, 2H), 7.50 (ddd, *J* = 8.7, 7.2, 1.6 Hz, 1H), 7.41 – 7.26 (m, 16H), 6.57 (d, *J* = 8.4 Hz, 1H), 5.26 (s, 2H).

^{13}C NMR (126 MHz, CDCl$_3$) δ 164.1, 162.1, 158.9, 149.1, 140.9, 135.1, 133.9, 131.8, 131.8, 131.2, 131.1, 130.3, 129.4, 128.9, 128.7, 123.9, 117.8, 117.7, 115.7, 115.6, 78.8.

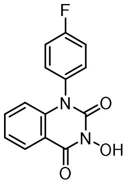

1-(4-fluorophenyl)-3-hydroxyquinazoline-2,4(1H,3H)-dione (94% yield)

^{1}H NMR (500 MHz, acetone) δ 9.69 (s, 1H), 8.16 (d, *J* = 7.9 Hz, 1H), 7.58 (q, *J* = 7.9 Hz, 3H), 7.42 (t, *J* = 8.7 Hz, 2H), 7.32 (t, *J* = 7.6 Hz, 1H), 6.61 (d, *J* = 8.4 Hz, 1H).

^{13}C NMR (126 MHz, acetone) δ 163.7, 161.8, 158.1, 148.5, 140.9, 134.5, 132.9, 132.9, 131.8, 131.7, 127.6, 123.0, 117.0, 116.9, 115.4, 115.2.

^{19}F NMR (471 MHz, acetone) δ -113.6.

General procedure C.

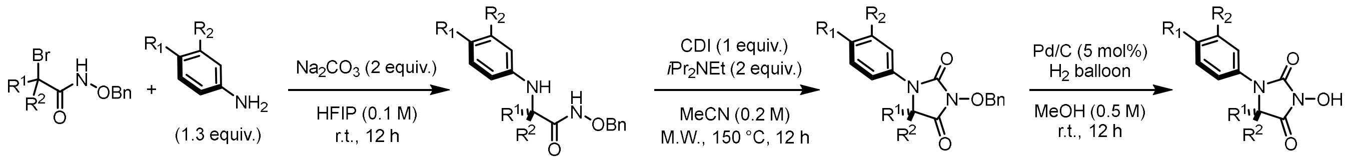

1) To a flame-dried flask, haloamide (5 mmol, 1.0 equiv.), aniline (1.3 equiv.), and Na_2CO_3 (2.0 equiv.) were dissolved in HFIP (0.3 M). Reaction mixture was then stirred at ambient temperature for 2 h. After the consumption of starting material, reaction mixture was quenched with water followed by workup with DCM and then ethyl acetate. Combined organic layers were dried over anhydrous Na_2SO_4. Evaporation of organic solvent gave crude which was purified over flash column chromatography (EtOAc/Hexanes) to afford the desired product.

2) To a solution of aminopropanamide (3 mmol, 1.0 equiv.) in MeCN (0.3 M), CDI (1.0 equiv.) and *i*Pr_2NEt (2 equiv.) were added. Reaction mixture was then stirred at reflux for 12 h. After cooling to room temperature, the solvent was removed under reduced pressure. The resulting crude was purified over flash column chromatography (EtOAc/Hexanes) to afford the desired product.

3) To a flask, benzyl protected hydantoin (1.0 equiv.) was dissolved in MeOH (0.2 M). The solution was sparged with argon for 15 min followed by addition of Pd/C (5 mol%). The resulting mixture was stirred at ambient temperature under hydrogen for 6 h. After the consumption of starting material, Pd/C was removed by vacuum filtration. Evaporation of organic solvent gave crude which was purified over flash column chromatography (EtOAc/Hexanes) to afford the desired product.

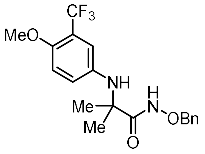

N-(benzyloxy)-2-((4-methoxy-3-(trifluoromethyl)phenyl)amino)-2-methylpropanamide (97% yield)

^{1}H NMR (600 MHz, cdcl$_3$) δ 9.37 (s, 1H), 7.36 – 7.29 (m, 5H), 6.86 (d, J = 2.9 Hz, 1H), 6.81 (d, J = 8.9 Hz, 1H), 6.72 – 6.68 (m, 1H), 4.91 (s, 2H), 3.82 (s, 3H), 1.46 (s, 6H).

^{13}C NMR (151 MHz, cdcl$_3$) δ 172.3, 151.3, 135.0, 129.2, 128.8, 128.5, 124.2, 122.4, 120.6, 119.5, 119.3, 116.2, 113.4, 112.0, 78.1, 58.3, 56.5, 25.8.

^{19}F NMR (564 MHz, cdcl$_3$) δ -62.4

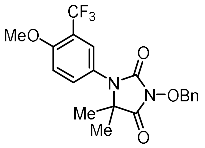

3-(benzyloxy)-1-(4-methoxy-3-(trifluoromethyl)phenyl)-5,5-dimethylimidazolidine-2,4-dione (83% yield)

^{1}H NMR (600 MHz, cdcl$_3$) δ 7.56 – 7.50 (m, 2H), 7.43 – 7.37 (m, 3H), 7.33 – 7.27 (m, 2H), 7.04 (d, J = 8.7 Hz, 1H), 5.24 (s, 2H), 3.93 (s, 3H), 1.30 (s, 6H).

^{13}C NMR (151 MHz, cdcl$_3$) δ 170.2, 157.6, 151.7, 134.1, 133.2, 130.5, 129.6, 128.5, 127.9, 125.2, 113.0, 112.0, 79.1, 62.3, 56.3, 23.4.

^{19}F NMR (564 MHz, cdcl$_3$) δ -63.0.

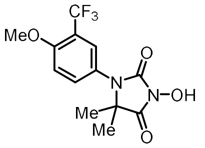

3-hydroxy-1-(4-methoxy-3-(trifluoromethyl)phenyl)-5,5-dimethylimidazolidine-2,4-dione (97% yield)

^{1}H NMR (600 MHz, cdcl$_3$) δ 9.12 (s, 1H), 7.43 – 7.36 (m, 2H), 7.05 (d, J = 8.7 Hz, 1H), 3.94 (s, 3H), 1.44 (s, 6H).

^{13}C NMR (151 MHz, cdcl$_3$) δ 171.1, 157.8, 153.3, 134.4, 128.0, 127.9, 124.7, 123.7, 121.8, 119.9, 113.1, 112.0, 62.8, 56.3, 23.3.

^{19}F NMR (564 MHz, cdcl$_3$) δ -62.9.

VIII. References

(1) Yang, C.; Farmer, L. A.; Pratt, D. A.; Maldonado, S.; Stephenson, C. R. J. Revisiting the Reactivity of the Dismissed Hydrogen Atom Transfer Catalyst Succinimide-*N*-oxyl. *J. Am. Chem. Soc.* **2024**, 146, 12511–12518.

(2) Sawyer, D. T.; Sobkowiak, A.; Roberts, J. L. *Electrochemistry for Chemists*, 2nd Edition, **1995**, Chapter 7.

(3) Yang, C.; Farmer, L. A.; McFee, E. C.; Jha, R. K.; Maldonado, S.; Pratt, D. A.; Stephenson, C. R. J. Attenuating *N*-oxyl Decomposition for Improved Hydrogen Atom Transfer Catalysts. *Angew. Chem. Int. Ed.* **2024**, 63, e202315917.

(4) Schrödinger Release 2023-1: MacroModel, Schrödinger, LLC, New York, NY, 2021.

(5) Lu, C.; Wu, C.; Ghoreishi, D.; Chen, W.; Wang, L.; Damm, W.; Ross, G.A.; Dahlgren, M.K.; Russell, E.; Von Bargen, C.D.; Abel, R.; Friesner, R.A.; Harder, E.D. OPLS4: Improving Force Field Accuracy on Challenging Regimes of Chemical Space. *J. Chem. Theory and Comput.* **2021** *17* (7), 4291.

(6) Kelley, L. A.; Gardner, S. P.; Sutcliffe, M. J. An automated approach for clustering an ensemble of NMR-derived protein structures into conformationally related subfamilies. Protein Eng., Des. Sel. **1996**, 9, 1063−1065.

(7) Gaussian 16, Revision C.01, Frisch, M. J.; Trucks, G. W.; Schlegel, H. B.; Scuseria, G. E.; Robb, M. A.; Cheeseman, J. R.; Scalmani, G.; Barone, V.; Petersson, G. A.; Nakatsuji, H.; Li, X.; Caricato, M.; Marenich, A. V.; Bloino, J.; Janesko, B. G.; Gomperts, R.; Mennucci, B.; Hratchian, H. P.; Ortiz, J. V.; Izmaylov, A. F.; Sonnenberg, J. L.; Williams-Young, D.; Ding, F.; Lipparini, F.; Egidi, F.; Goings, J.; Peng, B.; Petrone, A.; Henderson, T.; Ranasinghe, D.; Zakrzewski, V. G.; Gao, J.; Rega, N.; Zheng, G.; Liang, W.; Hada, M.; Ehara, M.; Toyota, K.; Fukuda, R.; Hasegawa, J.; Ishida, M.; Nakajima, T.; Honda, Y.; Kitao, O.; Nakai, H.; Vreven, T.; Throssell, K.; Montgomery, J. A., Jr.; Peralta, J. E.; Ogliaro, F.; Bearpark, M. J.; Heyd, J. J.; Brothers, E. N.; Kudin, K. N.; Staroverov, V. N.; Keith, T. A.; Kobayashi, R.; Normand, J.; Raghavachari, K.; Rendell, A. P.; Burant, J. C.; Iyengar, S. S.; Tomasi, J.; Cossi, M.; Millam, J. M.; Klene, M.; Adamo, C.; Cammi, R.; Ochterski, J. W.; Martin, R. L.; Morokuma, K.; Farkas, O.; Foresman, J. B.; Fox, D. J. Gaussian, Inc., Wallingford CT, 2016.

(8) Zhao, Y.; Truhlar, D.G. The M06 suite of density functionals for main group thermochemistry, thermochemical kinetics, noncovalent interactions, excited states, and transition elements: two new functionals and systematic testing of four M06-class functionals and 12 other functionals. *Theor. Chem. Account.* **2008** *120*, 215.

(9) Weigend, F.; Ahlrichs, R. Balanced basis sets of split valence, triple zeta valence and quadruple zeta valence quality for H to Rn: Design and assessment of accuracy. *Phys. Chem. Chem. Phys.* **2005** *7* (18), 3297.

(10) Glendening, E. D. ; Badenhoop, J, K..; Reed, A. E. ; Carpenter, J. E. ; Bohmann, J. A.; Morales, C. M.; Karafiloglou, P.; Landis, C. R.; Weinhold, F. NBO 7.0**.** Theoretical Chemistry Institute, University of Wisconsin, Madison. **2018**.

(11) github.com/SigmanGroup/Get_Properties

(12) github.com/digital-chemistry-laboratory/morfeus

(13) github.com/patonlab/GoodVibes

(14) Kennard, R. W.; Stone, L. A. Computer Aided Design of Experiments. Technometrics 1969, 11 (1), 137-148.

(15) Zell, D.; Kingston, C.; Jermaks, J.; Smith, S. R.; Seeger, N.; Wassmer, J.; Sirois, L. E.; Han, C.; Zhang, H. Sigman, M. S.; Gosselin, F. Stereoconvergent and -divergent Synthesis of Tetrasubstituted Alkenes by NickelCatalyzed Cross-Couplings. *J. Am. Chem. Soc*. **2021**, 143, 45, 19078.

(16) Nechab, M.; Einhorn, C.; Einhorn, J. New aerobic oxidation of benzylic compounds: efficient catalysis by N-hydroxy-3,4,5,6-tetraphenylphthalimide (NHTPPI)/CuCl under mild conditions and low catalyst loading. *Chem. Commun.* **2004**, 1500-1501.

(17) Kadoh, Y.; Oisaki, K.; Kanai, M. Enhanced Structural Variety of Nonplanar N-Oxyl Radical Catalysts and Their Application to the Aerobic Oxidation of Benzylic C–H Bonds. *Chem. Pharm. Bull.* **2016**, 64, 737–753.

(18) Yang, C.; Farmer, L. A.; Ghosh, S.; Arora, S.; Swiecki, S. Pushing the Limit of Bond Dissociation Enthalpy in the Design of *N*-oxyl Hydrogen Atom Transfer Catalysts. (back-to-back submission)

(19) Zhu, C.; Liang, Y.; Hong, X.; Sun, H.; Sun, W.; Houk, K. N.; Shi, Z. Iodoarene-Catalyzed Stereospecific Intramolecular sp3 C–H Amination: Reaction Development and Mechanistic Insights. *J. Am. Chem. Soc.* **2015**, 137, 7564–7567.

(20) Goldstein, E. L.; Takada, H.; Sumii, Y.; Baba, K.; Stoltz, B. M. Synthesis of enantioenriched 2,2-disubstituted pyrrolidines via sequential asymmetric allylic alkylation and ring contraction. *Tetrahedron* **2022**, 123, 132940.

(21) Takaya, T.; Yoshimoto, H.; Imoto, E. Convenient Syntheses of 1,2-Ureylenecyclopentane Derivatives. *Bulletin of the Chemical Society of Japan*, **1967**, 40, 2844–2849.

(22) Czyzyk, D. J.; Valhondo, M.; Deiana, L.; Tirado-Rives, J.; Jorgensen, W. L.; Anderson, K. S. Structure activity relationship towards design of cryptosporidium specific thymidylate synthase inhibitors. *European Journal of Medicinal Chemistry* **2019**, 183, 111673.

(23) Sati, G. C.; Crich, D. Facile Synthesis of 3-N-Alkyl Pyrimidin-2,4-diones from N-Sulfonyloxy Maleimides and Amines. *Org. Lett.* **2015**, 17, 4122–4124.

(24) Kato, T.; Maruoka, K. Design of Bowl-Shaped N-Hydroxyimide Derivatives as New Organoradical Catalysts for Site-Selective C(sp3)−H Bond Functionalization Reactions. *Angew. Chem. Int. Ed.* **2020**, 59, 14261–14264.

(25) Wu, J.; Kuo, C.; Chu, C.; Chen, M.; Lin, J.; Chen, Y.; Liao, H. Synthesis of Novel Lipophilic N-Substituted Norcantharimide Derivatives and Evaluation of Their Anticancer Activities. *Molecules* **2014**, 19, 6911-6928.

(26) Bayer, T.; Chakrabarti, A.; Lancelot, J.; Dr. Shaik, T. B.; Hausmann, K.; Melesina, J.; Schmidtkunz, K.; Marek, M.; Erdmann, F.; Schmidt, M.; Robaa, D.; Romier, C.; Pierce, R. J.; Jung, M.; Sippl, W. Synthesis, Crystallization Studies, and in vitro Characterization of Cinnamic Acid Derivatives as SmHDAC8 Inhibitors for the Treatment of Schistosomiasis. *ChemMedChem* **2018**, 13, 1517–1529.

IX. NMR spectra

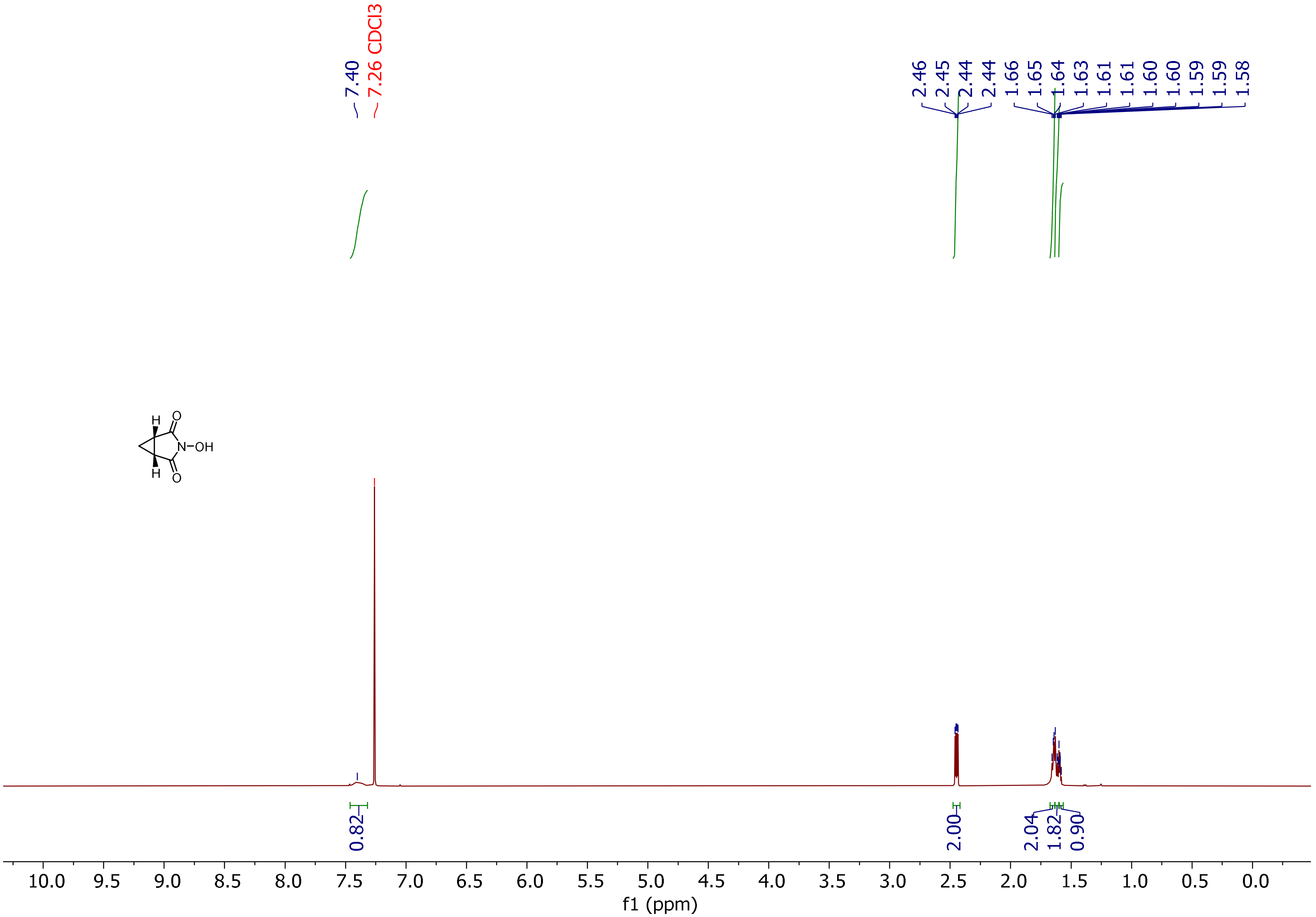

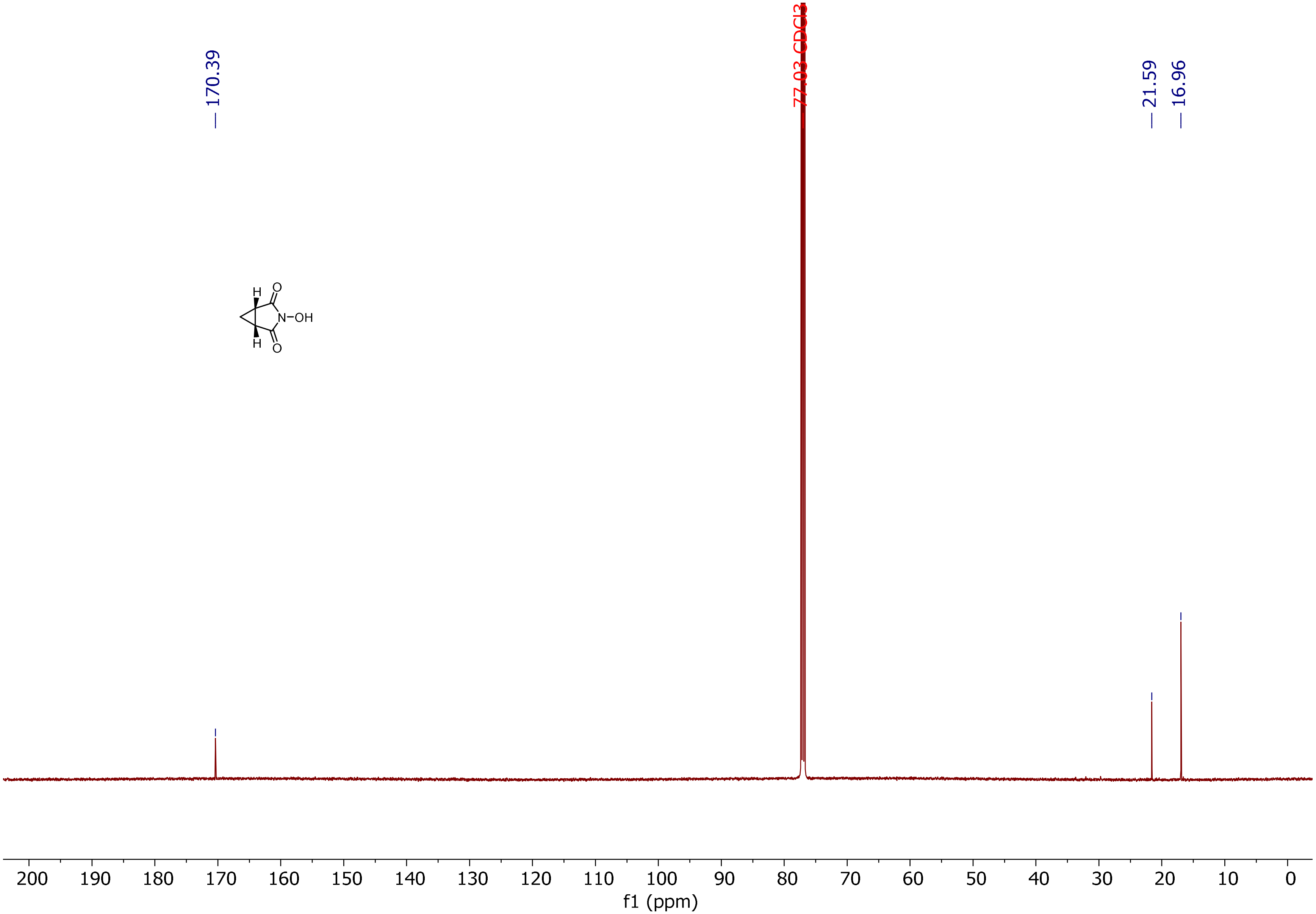

170.39
77.03 CDCl3
21.59
16.96
H
O
N-OH
H
O
200
190
180
170
160
150
140
130
120
110
100
90
80
70
60
50
40
30
20
10
0
f1 (ppm)

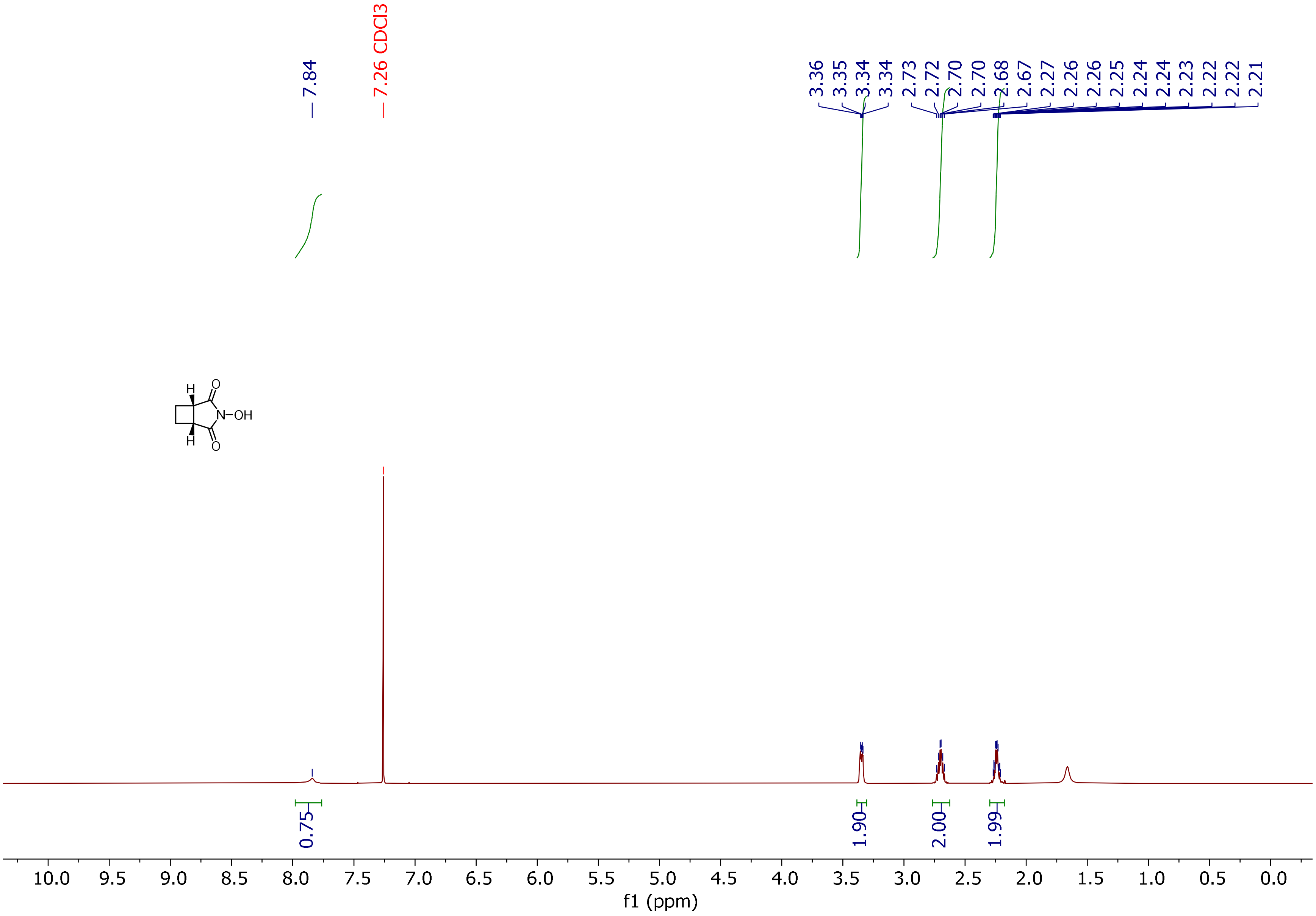
7.84
7.26 CDCl3
3.36
3.35
3.34
3.34
2.73
2.72
2.70
2.70
2.68
2.67
2.27
2.26
2.26
2.25
2.24
2.24
2.23
2.22
2.22
2.21
H
O
N-OH
H
O
0.75
1.90
2.00
1.99
10.0
9.5
9.0
8.5
8.0
7.5
7.0
6.5
6.0
5.5
5.0
4.5
4.0
3.5
3.0
2.5
2.0
1.5
1.0
0.5
0.0
f1 (ppm)

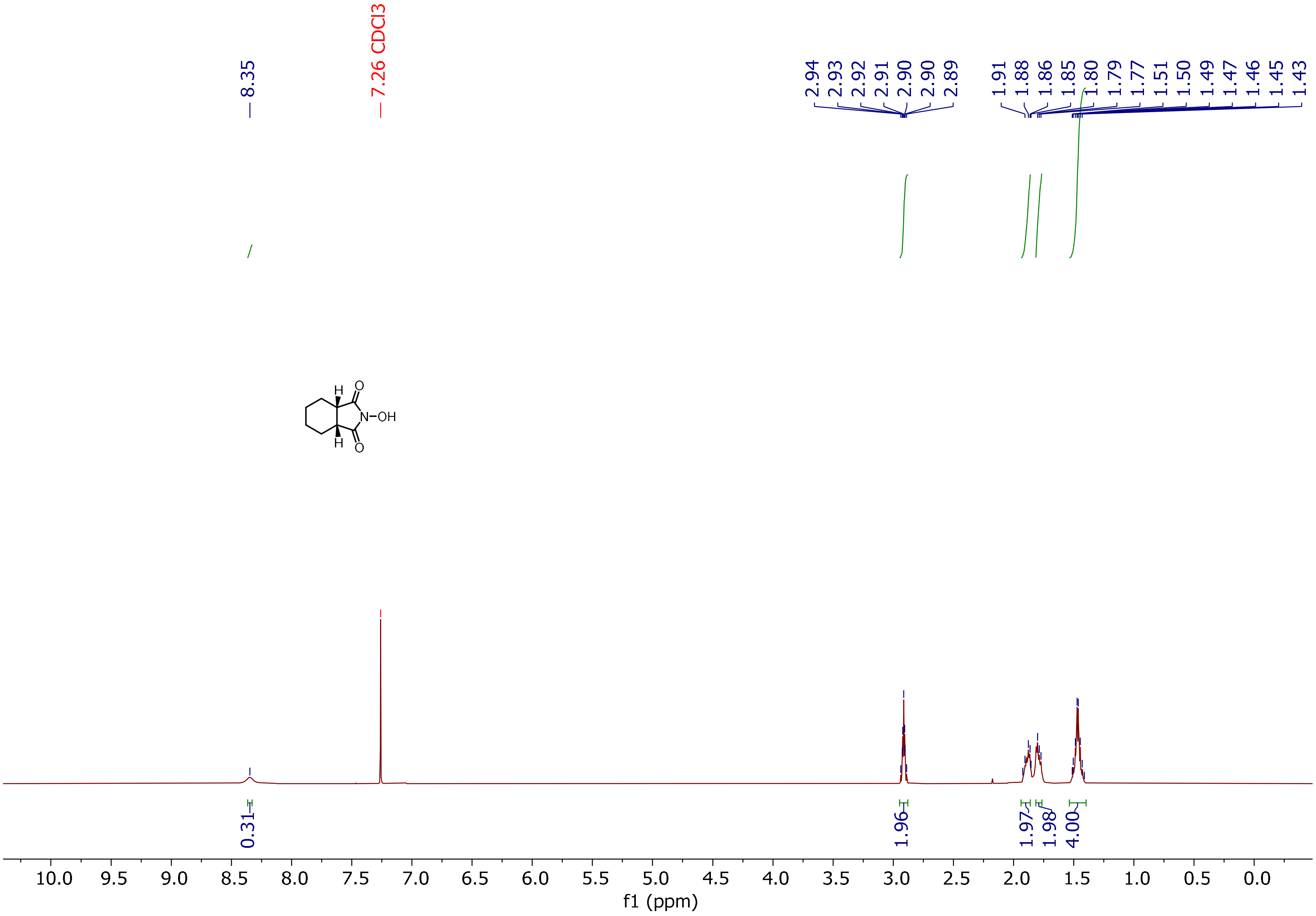

8.35
7.26 CDCl3
2.94 2.93 2.92 2.91 2.90 2.90 2.89
1.91 1.88 1.86 1.85 1.80 1.79 1.77 1.51 1.50 1.49 1.47 1.46 1.45 1.43
N–OH
H
O
0.31
1.96
1.97
1.98
4.00
10.0 9.5 9.0 8.5 8.0 7.5 7.0 6.5 6.0 5.5 5.0 4.5 4.0 3.5 3.0 2.5 2.0 1.5 1.0 0.5 0.0
f1 (ppm)

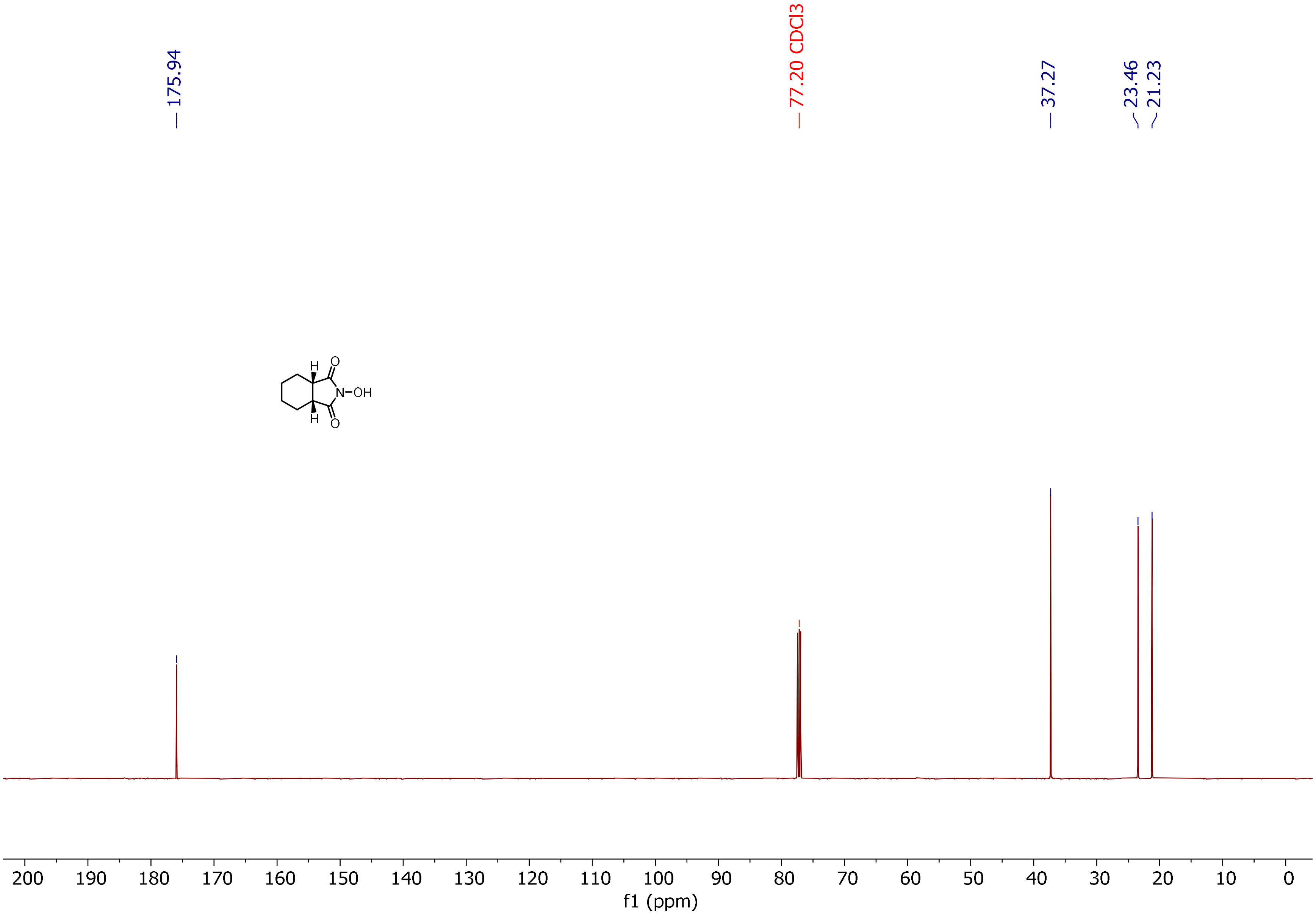

175.94
77.20 CDCl3
37.27
23.46
21.23
H
O
N–OH
H
O
200 190 180 170 160 150 140 130 120 110 100 90 80 70 60 50 40 30 20 10 0
f1 (ppm)

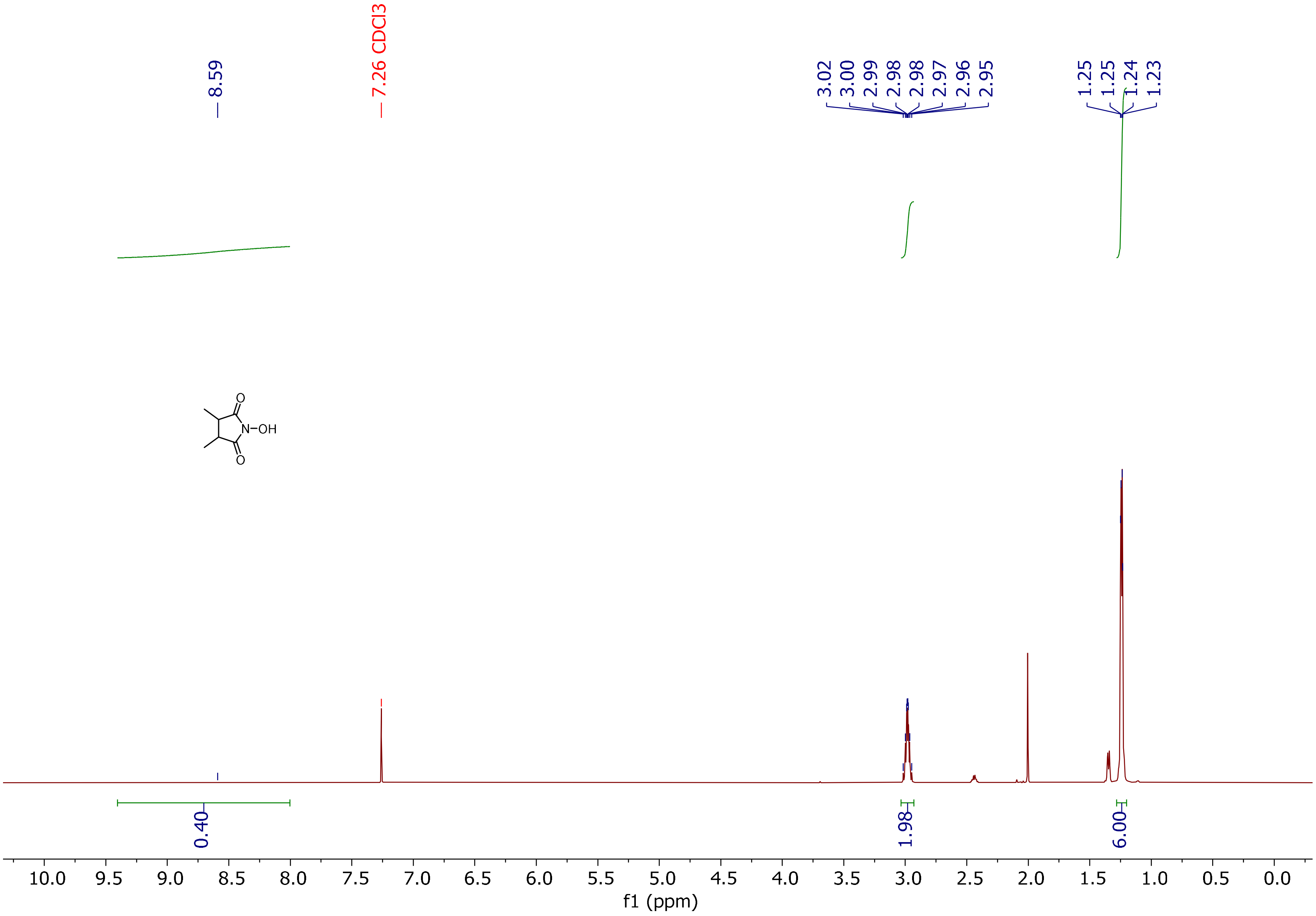

8.59
7.26 CDCl3
3.02
3.00
2.99
2.98
2.98
2.97
2.96
2.95
1.25
1.25
1.24
1.23
O
N–OH
O
0.40
1.98
6.00
10.0
9.5
9.0
8.5
8.0
7.5
7.0
6.5
6.0
5.5
5.0
4.5
4.0
3.5
3.0
2.5
2.0
1.5
1.0
0.5
0.0
f1 (ppm)

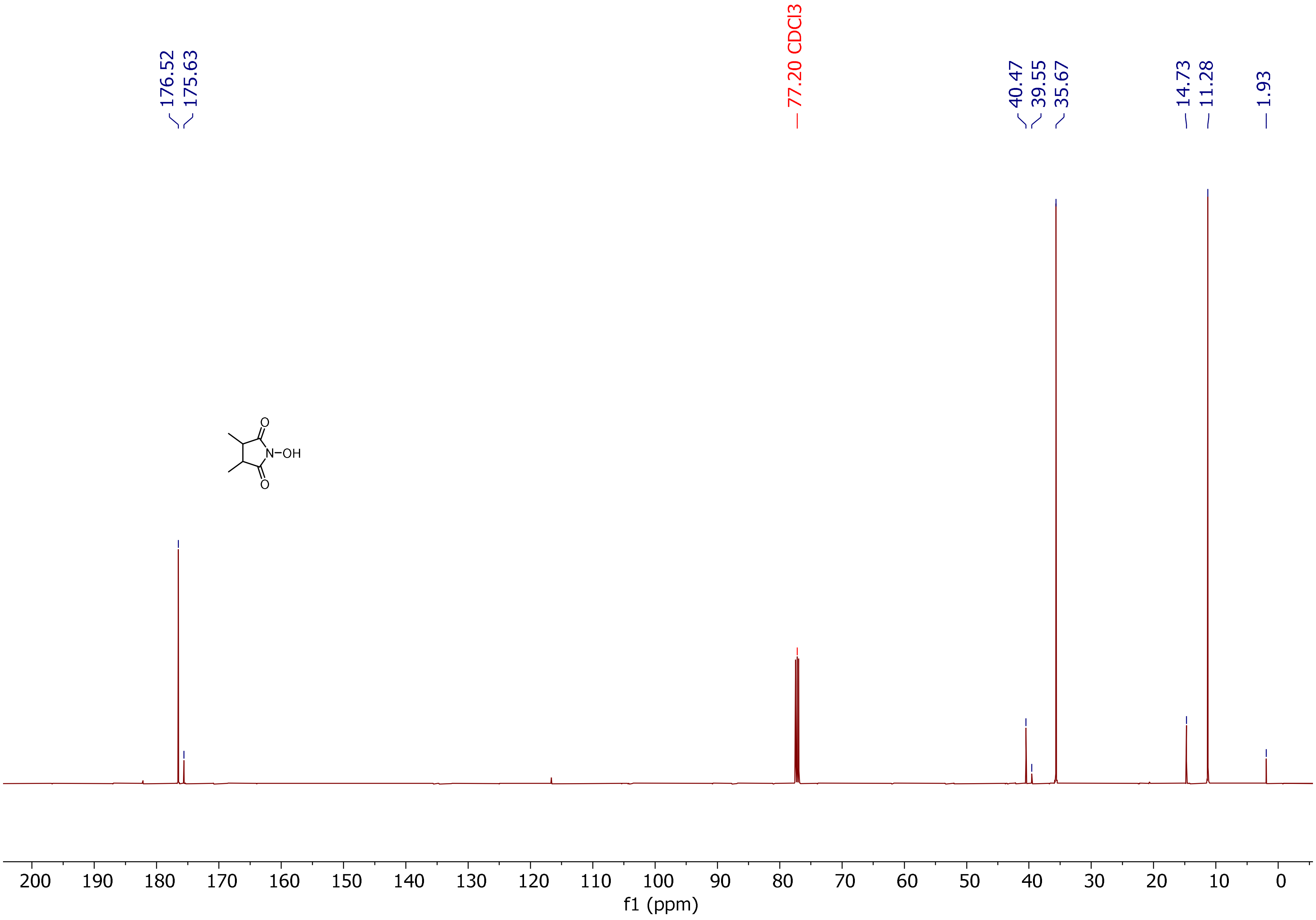

176.52
175.63
77.20 CDCl3
40.47
39.55
35.67
14.73
11.28
1.93
N–OH
O
O
200 190 180 170 160 150 140 130 120 110 100 90 80 70 60 50 40 30 20 10 0
f1 (ppm)

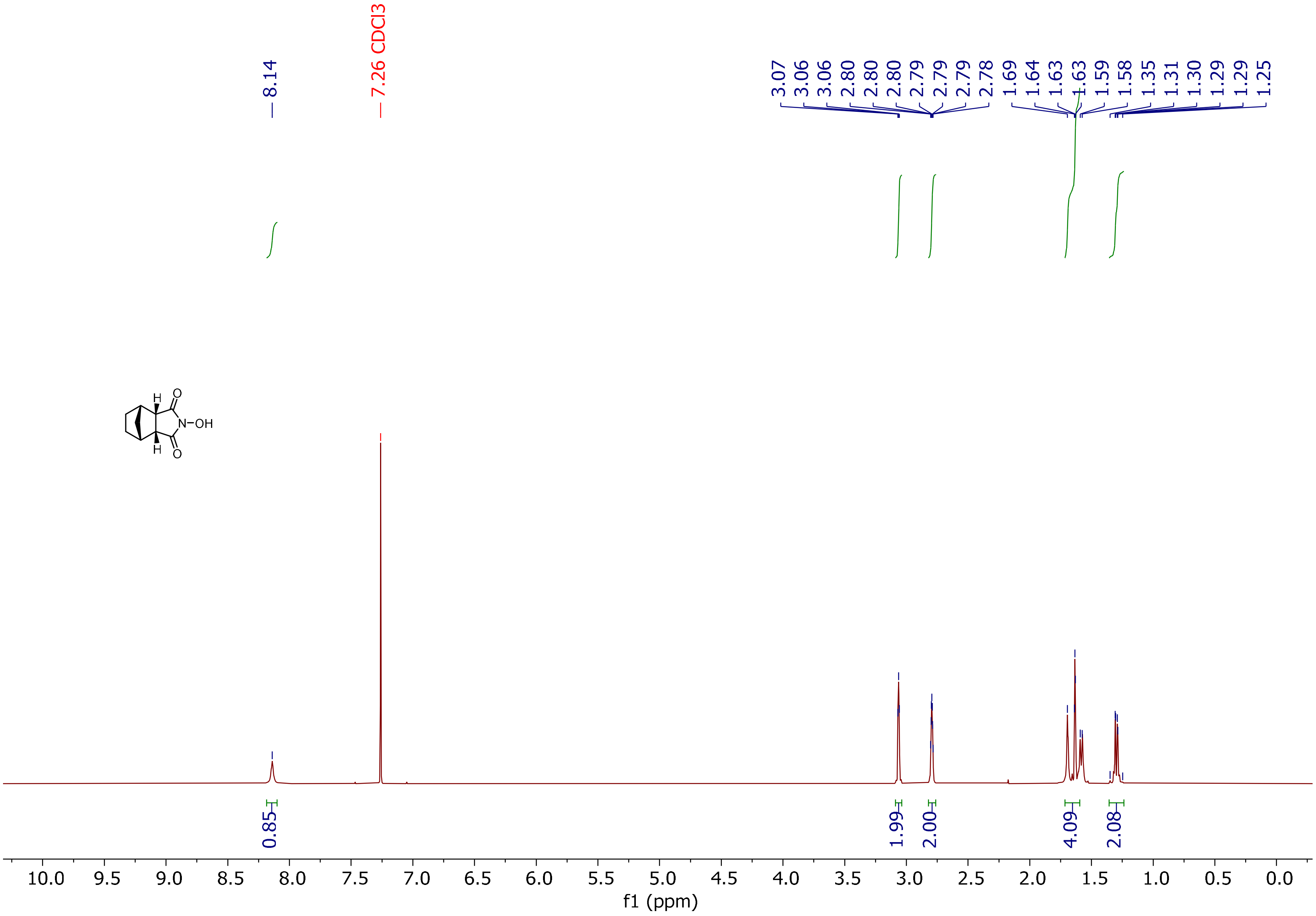

8.14
7.26 CDCl3
3.07
3.06
3.06
2.80
2.80
2.80
2.79
2.79
2.79
2.78
1.69
1.64
1.63
1.63
1.59
1.58
1.35
1.31
1.30
1.29
1.29
1.25
N-OH
H
H
O
O
0.85
1.99
2.00
4.09
2.08
10.0 9.5 9.0 8.5 8.0 7.5 7.0 6.5 6.0 5.5 5.0 4.5 4.0 3.5 3.0 2.5 2.0 1.5 1.0 0.5 0.0
f1 (ppm)

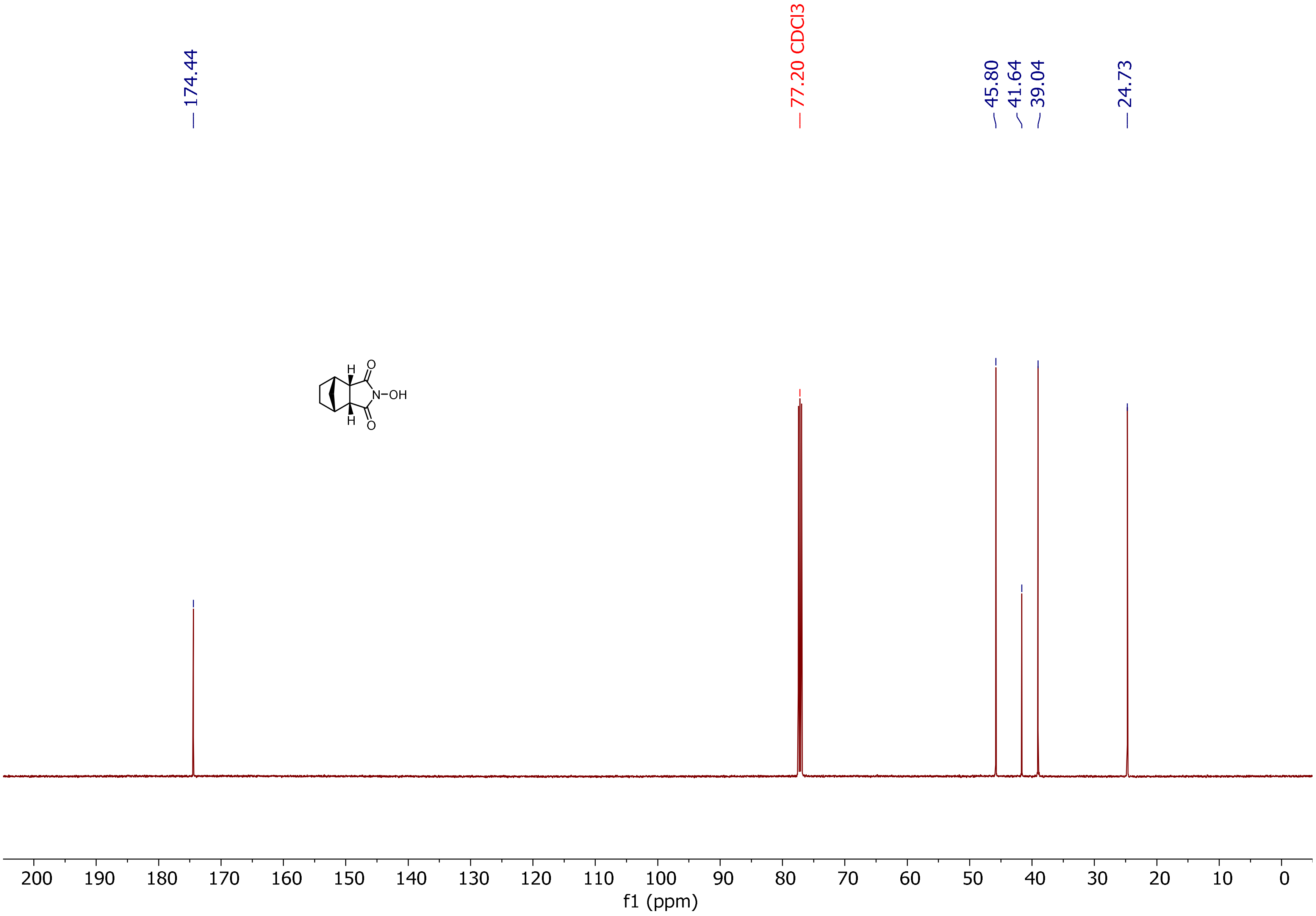

174.44
77.20 CDCl3
45.80
41.64
39.04
24.73
H
O
N-OH
H
O
200
190
180
170
160
150
140
130
120
110
100
90
80
70
60
50
40
30
20
10
0
f1 (ppm)

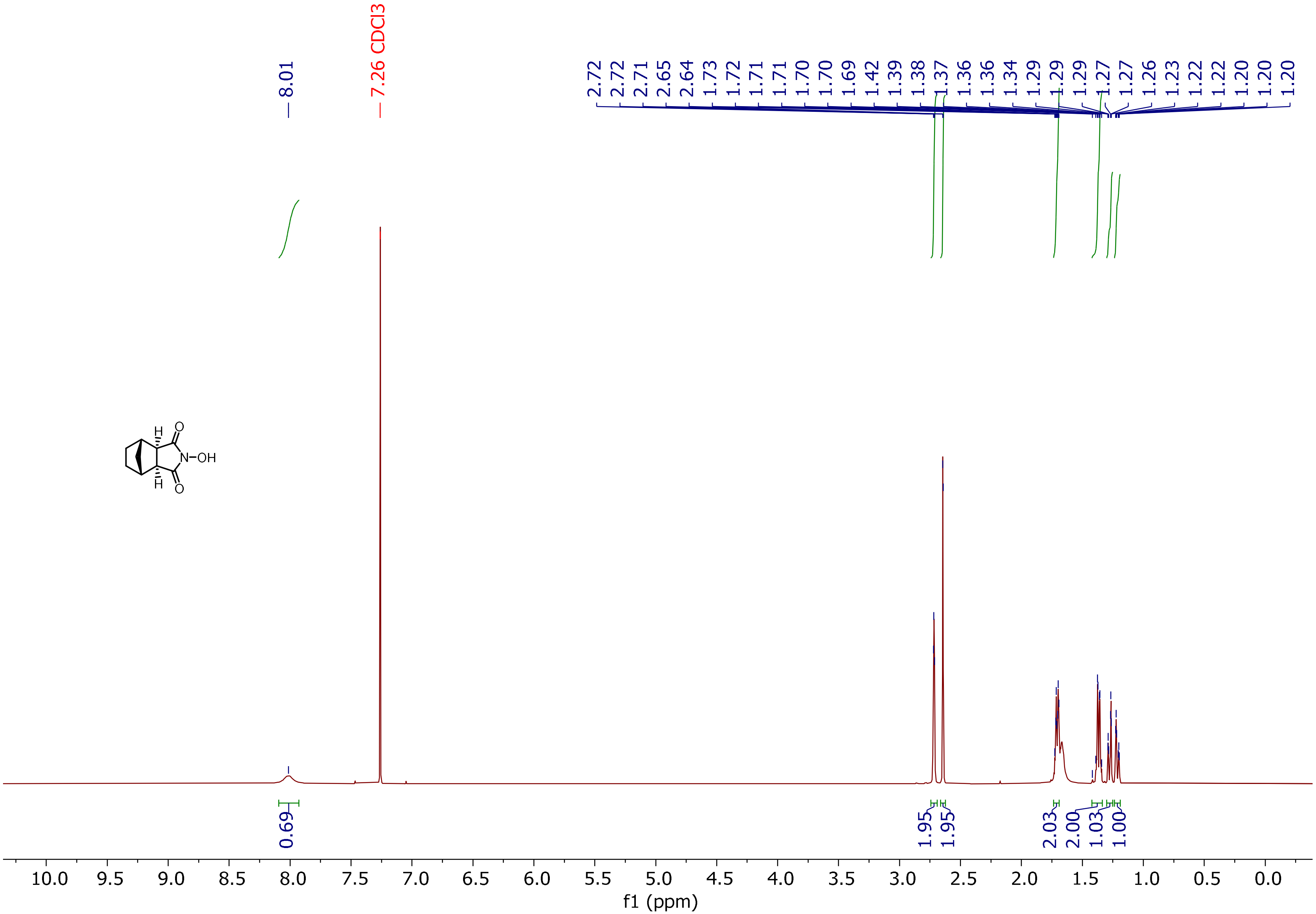

8.01
7.26 CDCl3
2.72
2.72
2.71
2.65
2.64
1.73
1.72
1.71
1.71
1.70
1.70
1.69
1.42
1.39
1.38
1.37
1.36
1.36
1.34
1.29
1.29
1.29
1.27
1.27
1.26
1.23
1.22
1.22
1.20
1.20
1.20
0.69
1.95
1.95
2.03
2.00
1.03
1.00
10.0
9.5
9.0
8.5
8.0
7.5
7.0
6.5
6.0
5.5
5.0
4.5
4.0
3.5
3.0
2.5
2.0
1.5
1.0
0.5
0.0
f1 (ppm)

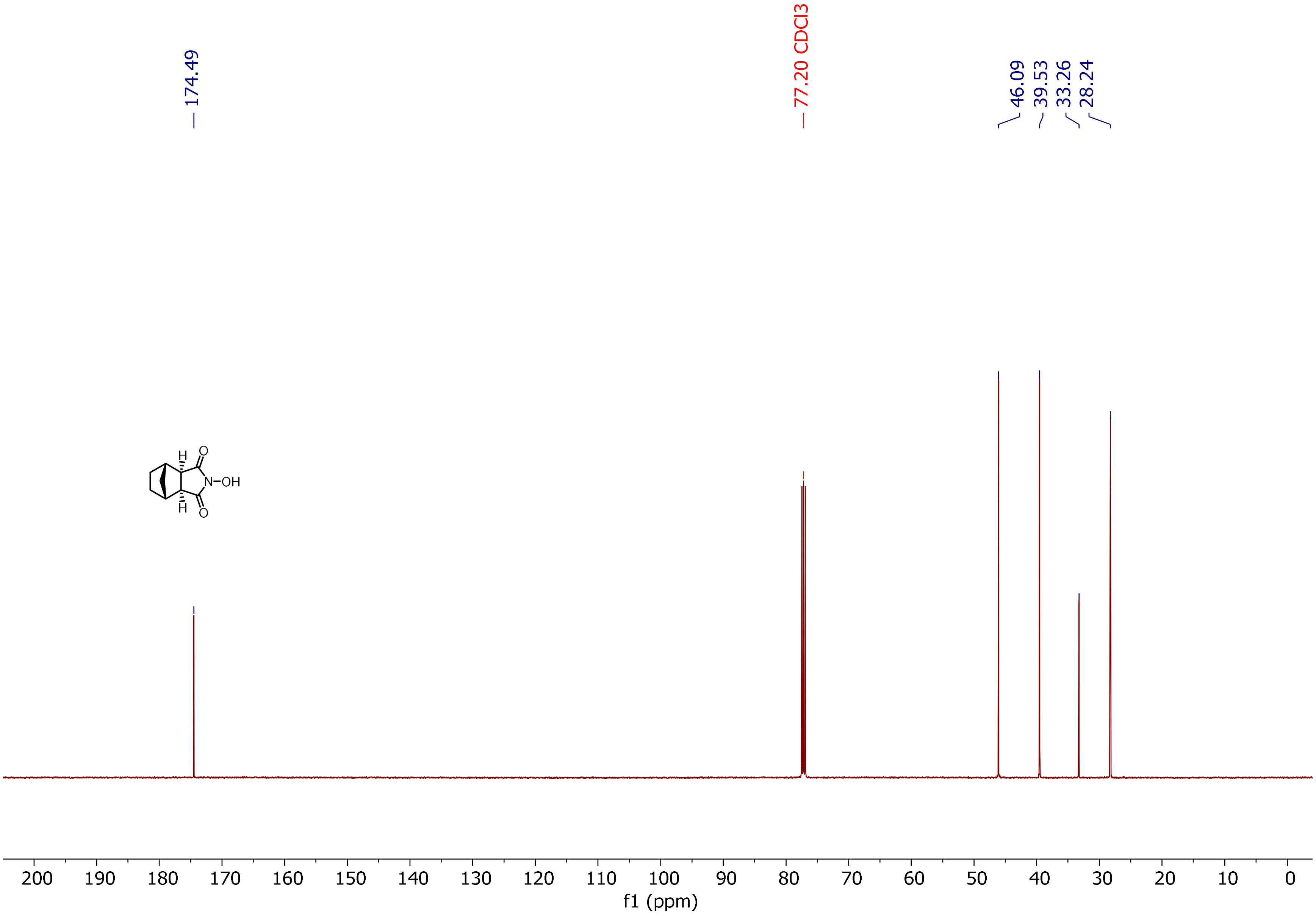
174.49
77.20 CDCl3
46.09
39.53
33.26
28.24
N–OH
H
H
O
O
200
190
180
170
160
150
140
130
120
110
100
90
80
70
60
50
40
30
20
10
0
f1 (ppm)

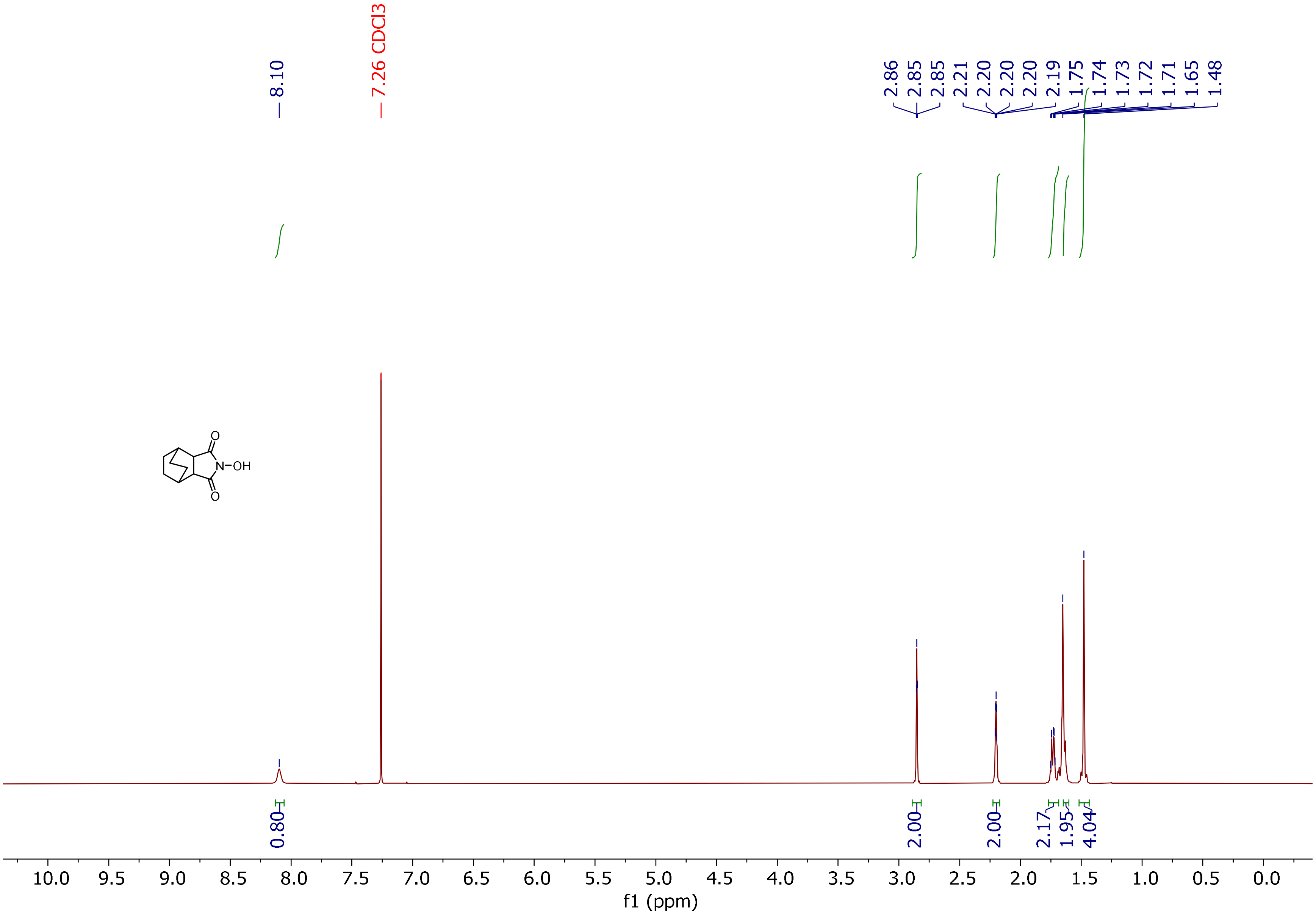

8.10
7.26 CDCl3
2.86
2.85
2.85
2.21
2.20
2.20
2.20
2.19
1.75
1.74
1.73
1.72
1.71
1.65
1.48
N-OH
O
O
0.80
2.00
2.00
2.17
1.95
4.04
10.0
9.5
9.0
8.5
8.0
7.5
7.0
6.5
6.0
5.5
5.0
4.5
4.0
3.5
3.0
2.5
2.0
1.5
1.0
0.5
0.0
f1 (ppm)

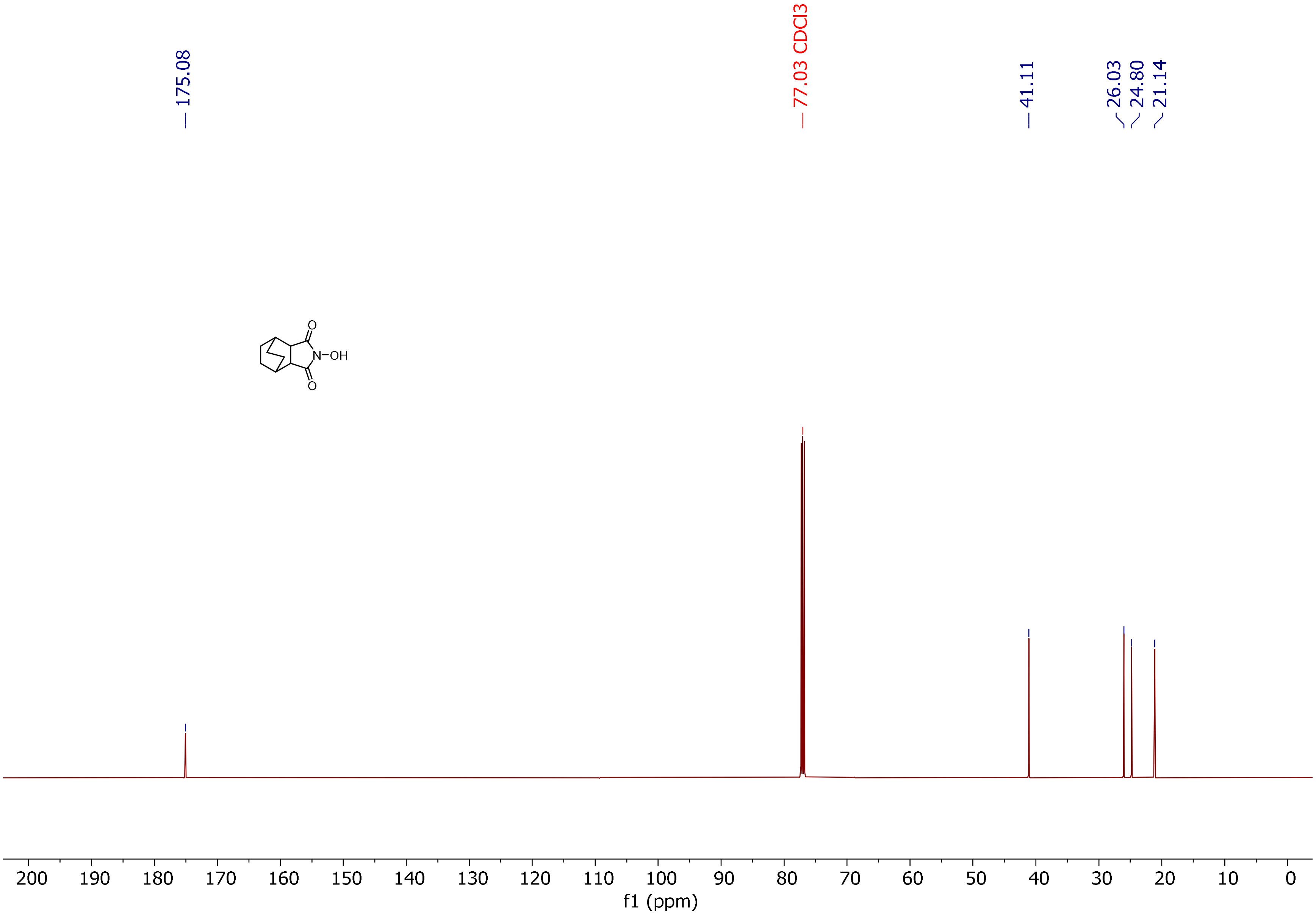

175.08
77.03 CDCl3
41.11
26.03
24.80
21.14
N-OH
O
O
200
190
180
170
160
150
140
130
120
110
100
90
80
70
60
50
40
30
20
10
0
f1 (ppm)

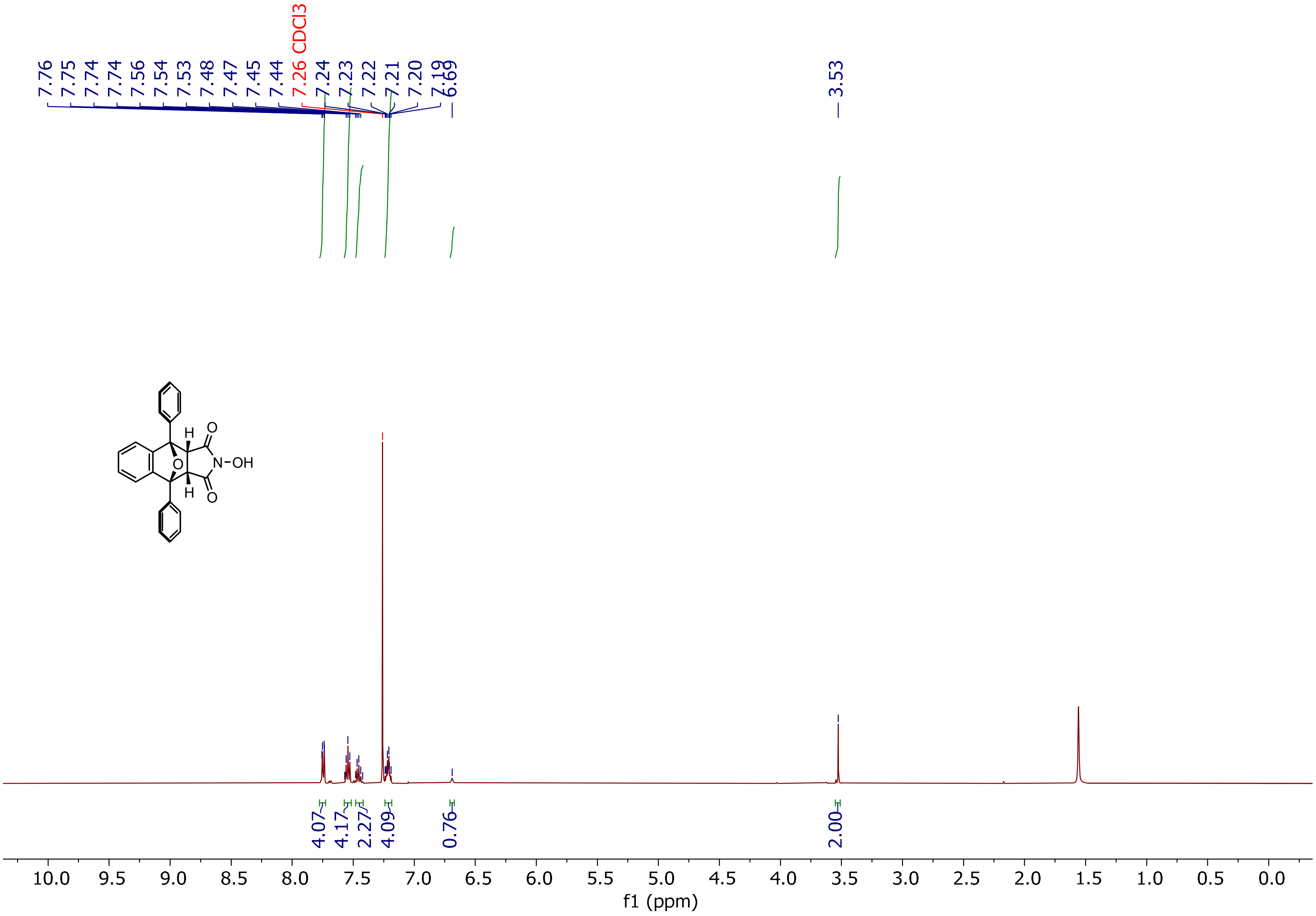

7.76
7.75
7.74
7.74
7.56
7.54
7.53
7.48
7.47
7.45
7.44
7.26 CDCl3
7.24
7.23
7.22
7.21
7.20
7.19
6.69
3.53
H
O
N-OH
O
H
O
4.07
4.17
2.27
4.09
0.76
2.00
10.0
9.5
9.0
8.5
8.0
7.5
7.0
6.5
6.0
5.5
5.0
4.5
4.0
3.5
3.0
2.5
2.0
1.5
1.0
0.5
0.0
f1 (ppm)

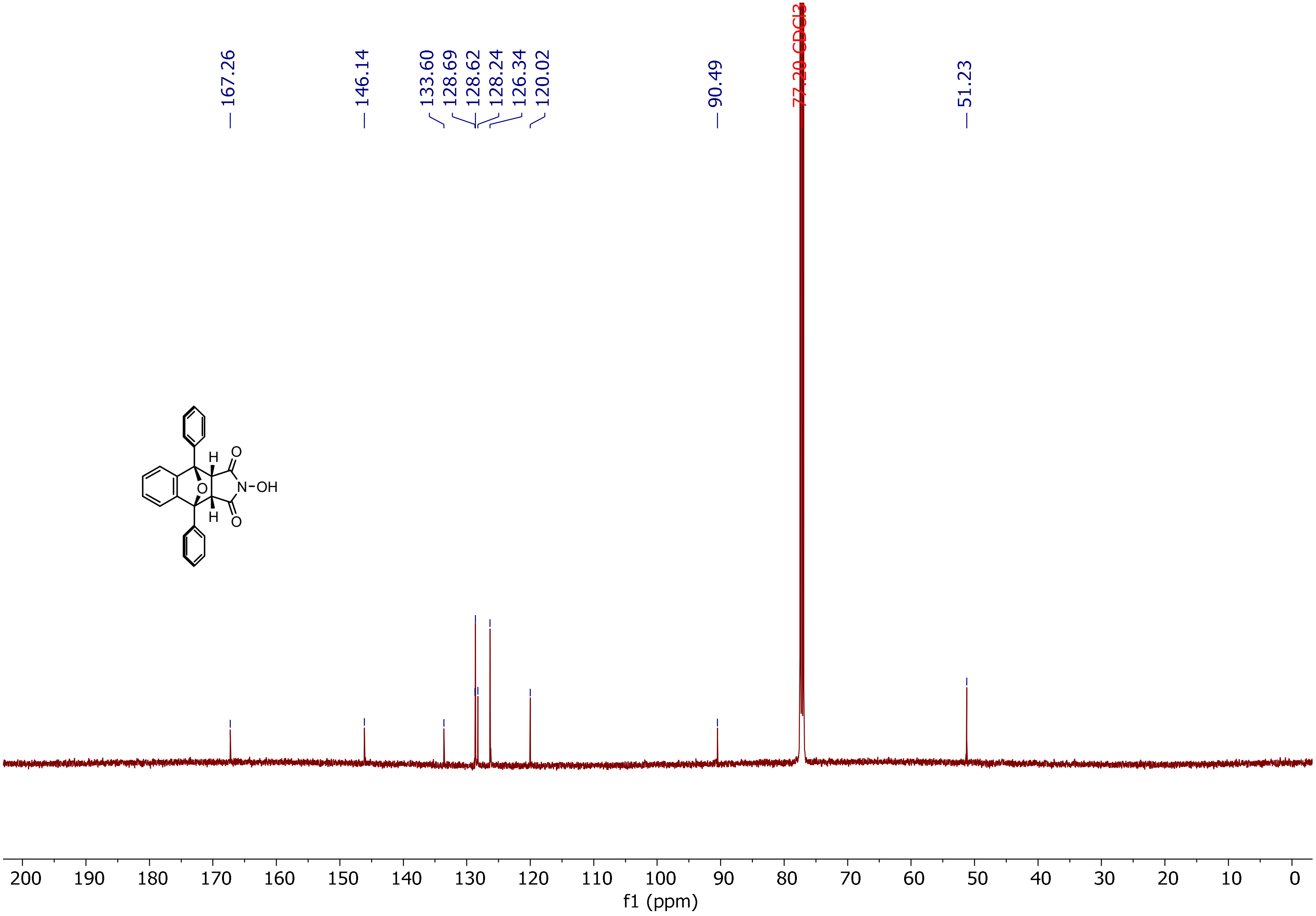

167.26
146.14
133.60
128.69
128.62
128.24
126.34
120.02
90.49
77.20 CDCl3
51.23
200 190 180 170 160 150 140 130 120 110 100 90 80 70 60 50 40 30 20 10 0
f1 (ppm)
N-OH

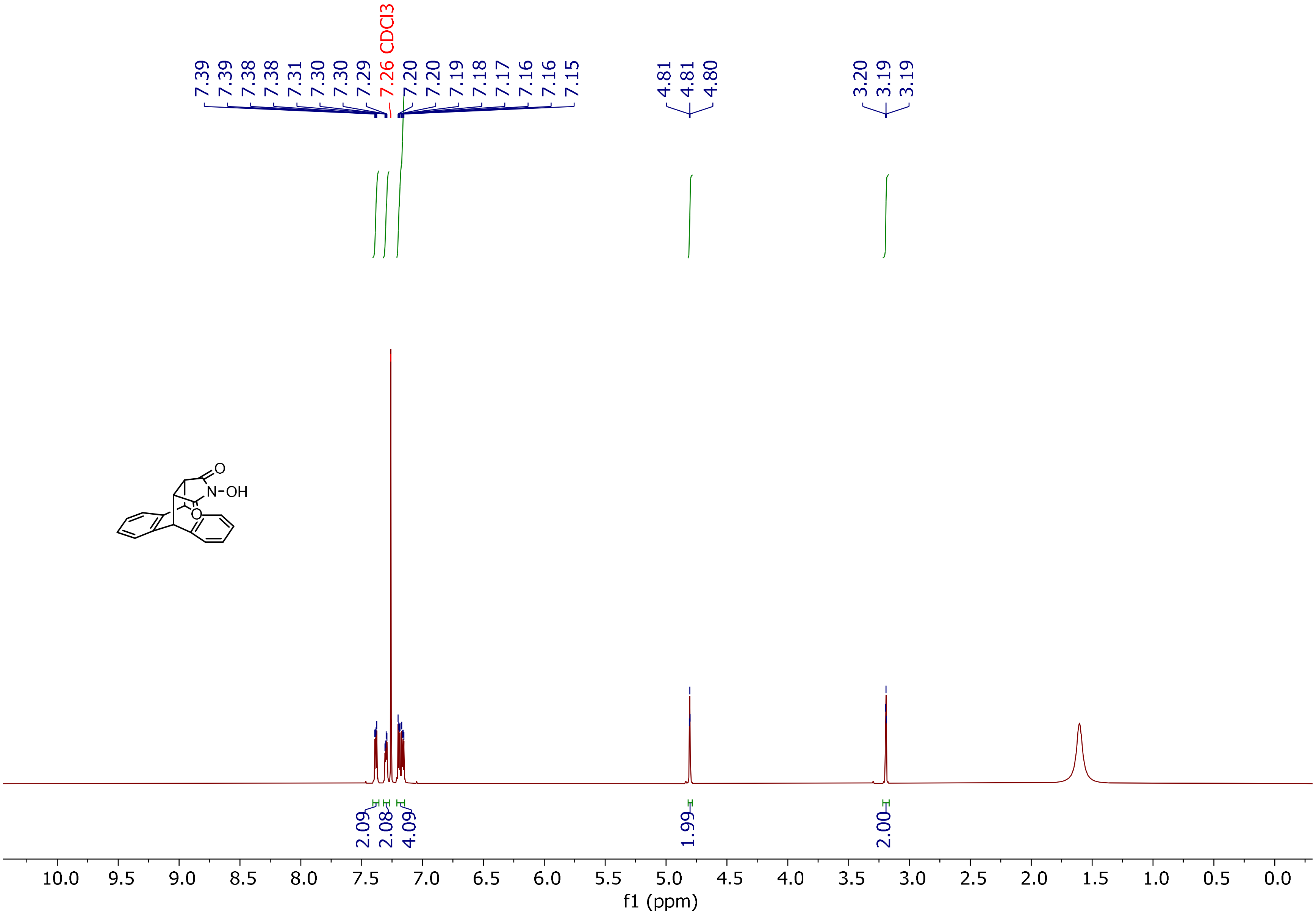

7.39
7.39
7.38
7.38
7.31
7.30
7.30
7.29
7.26 CDCl3
7.20
7.20
7.19
7.18
7.17
7.16
7.16
7.15
4.81
4.81
4.80
3.20
3.19
3.19
N-OH
O
O
2.09
2.08
4.09
1.99
2.00
10.0
9.5
9.0
8.5
8.0
7.5
7.0
6.5
6.0
5.5
5.0
4.5
4.0
3.5
3.0
2.5
2.0
1.5
1.0
0.5
0.0
f1 (ppm)

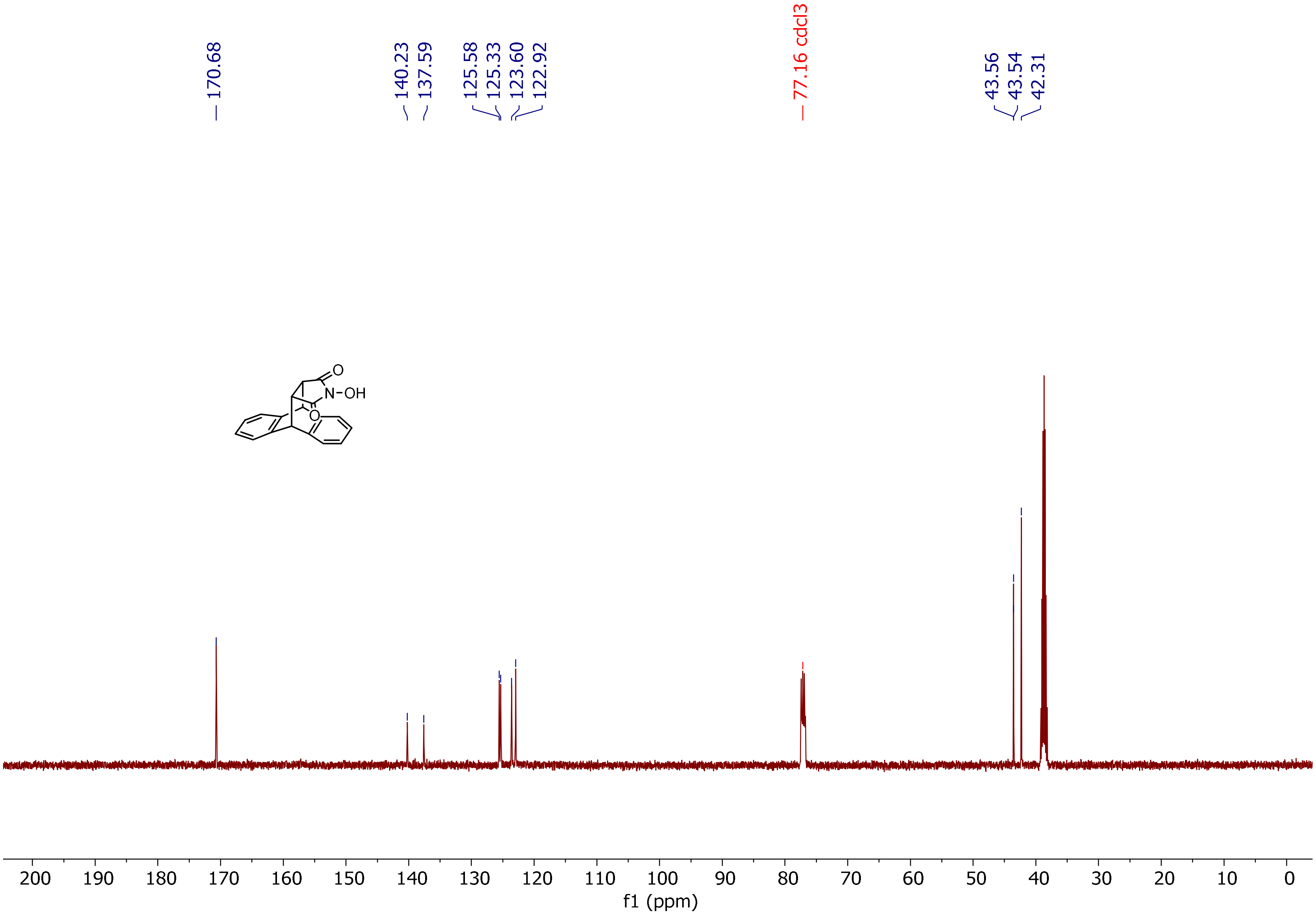

170.68
140.23
137.59
125.58
125.33
123.60
122.92
77.16 cdcl3
43.56
43.54
42.31
O
N-OH
O
200
190
180
170
160
150
140
130
120
110
100
90
80
70
60
50
40
30
20
10
0
f1 (ppm)

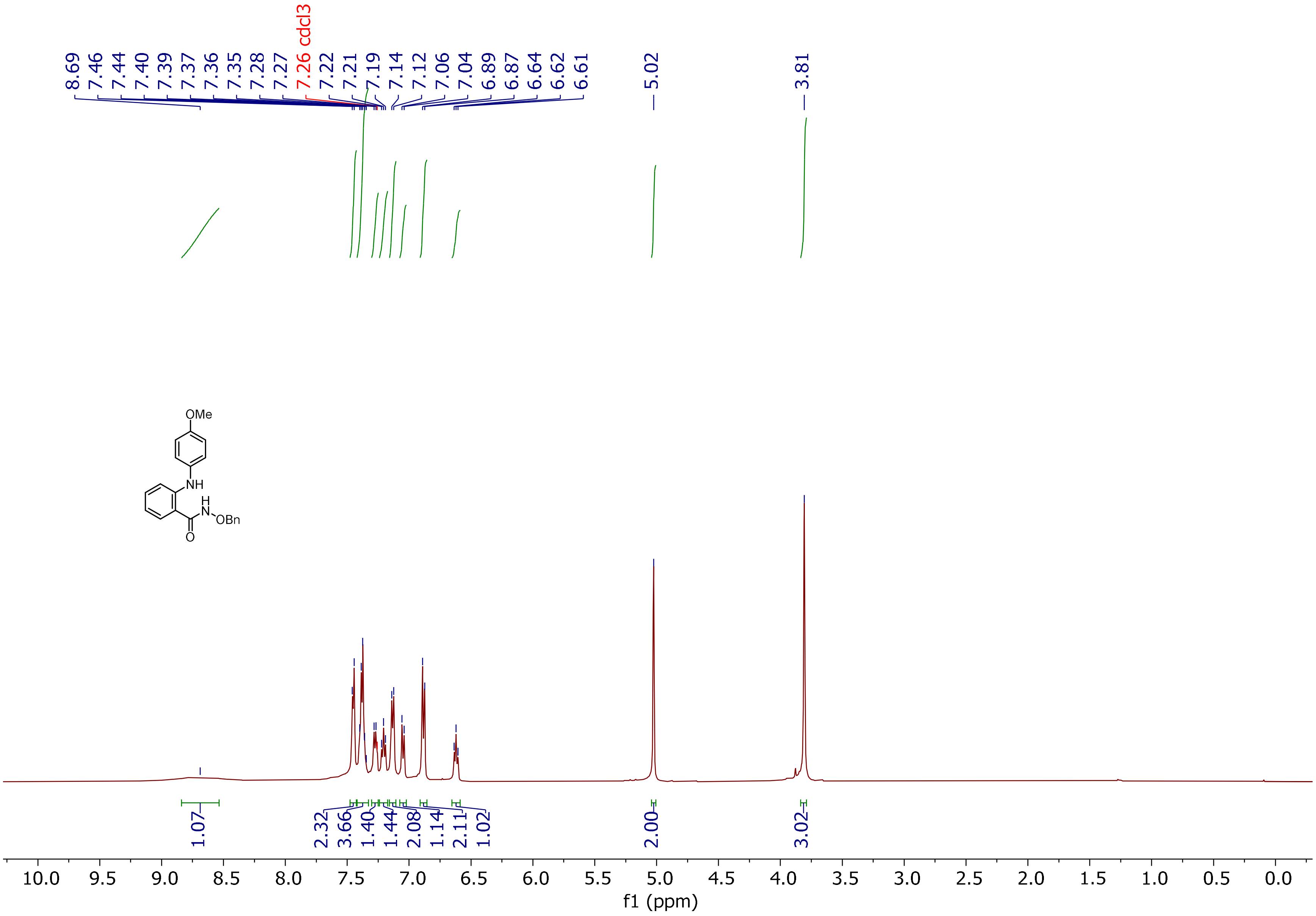

8.69
7.46
7.44
7.40
7.39
7.37
7.36
7.35
7.28
7.27
7.26 cdcl3
7.22
7.21
7.19
7.14
7.12
7.06
7.04
6.89
6.87
6.64
6.62
6.61
5.02
3.81
OMe
NH
H
N
OBn
O
1.07
2.32
3.66
1.40
1.44
2.08
1.14
2.11
1.02
2.00
3.02
10.0
9.5
9.0
8.5
8.0
7.5
7.0
6.5
6.0
5.5
5.0
4.5
4.0
3.5
3.0
2.5
2.0
1.5
1.0
0.5
0.0
f1 (ppm)

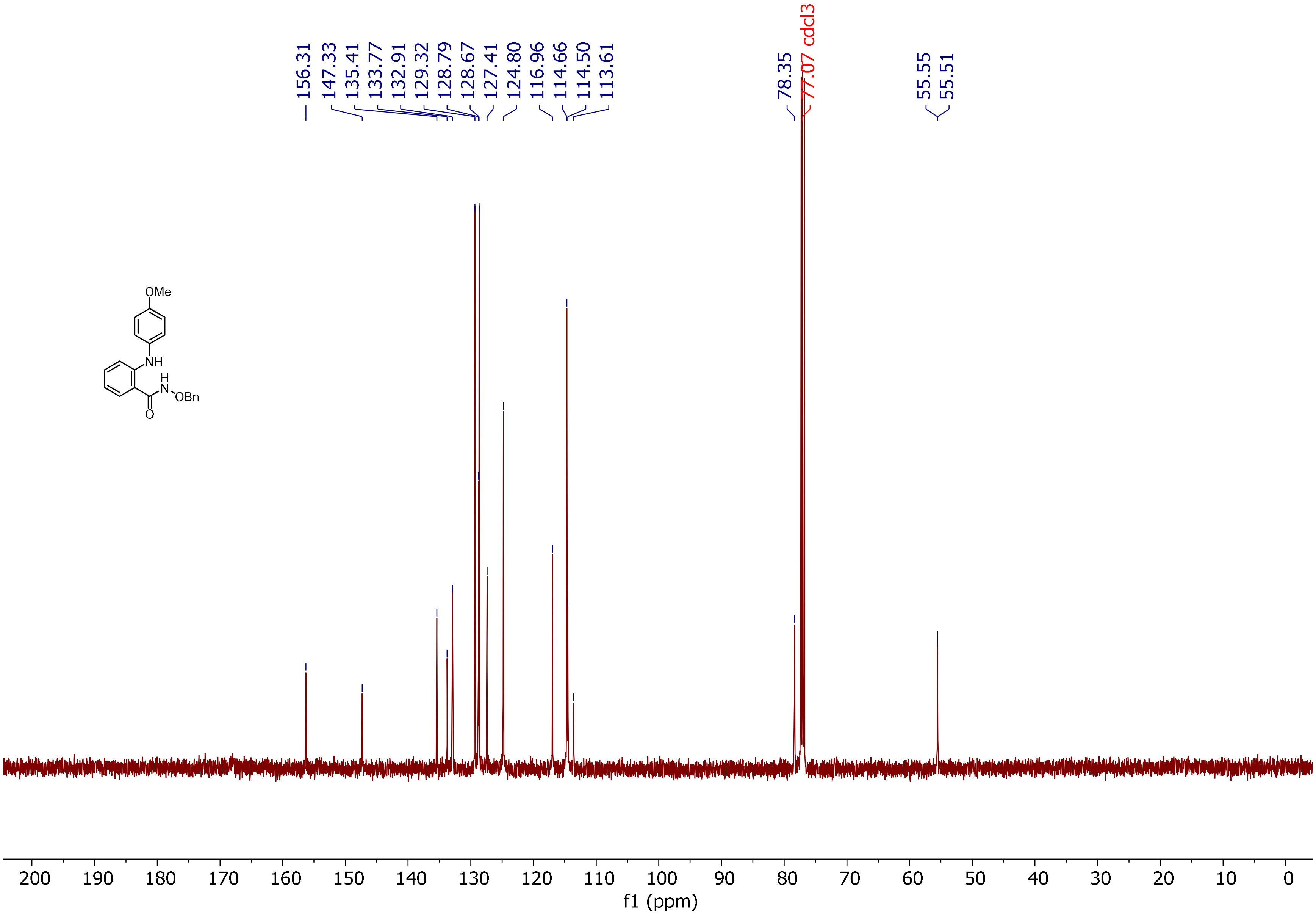

156.31
147.33
135.41
133.77
132.91
129.32
128.79
128.67
127.41
124.80
116.96
114.66
114.50
113.61
78.35
77.07 cdcl3
55.55
55.51
OMe
NH
H
N
OBn
O
200 190 180 170 160 150 140 130 120 110 100 90 80 70 60 50 40 30 20 10 0
f1 (ppm)

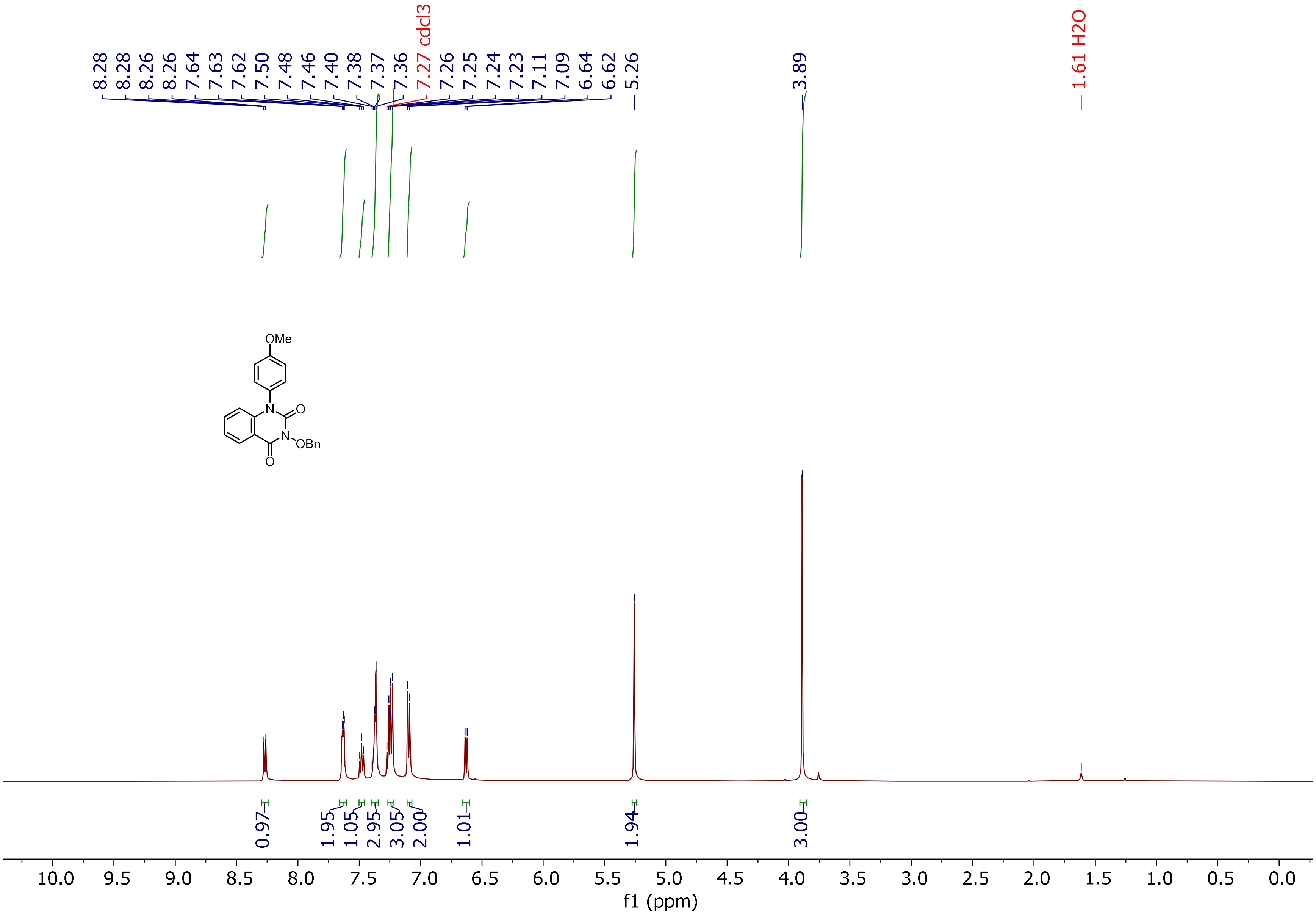
8.28
8.28
8.26
8.26
7.64
7.63
7.62
7.50
7.48
7.46
7.40
7.38
7.37
7.36
7.27 cdcl3
7.26
7.25
7.24
7.23
7.11
7.09
6.64
6.62
5.26
3.89
1.61 H2O
OMe
N
O
N
OBn
O
0.97
1.95
1.05
2.95
3.05
2.00
1.01
1.94
3.00
10.0
9.5
9.0
8.5
8.0
7.5
7.0
6.5
6.0
5.5
5.0
4.5
4.0
3.5
3.0
2.5
2.0
1.5
1.0
0.5
0.0
f1 (ppm)

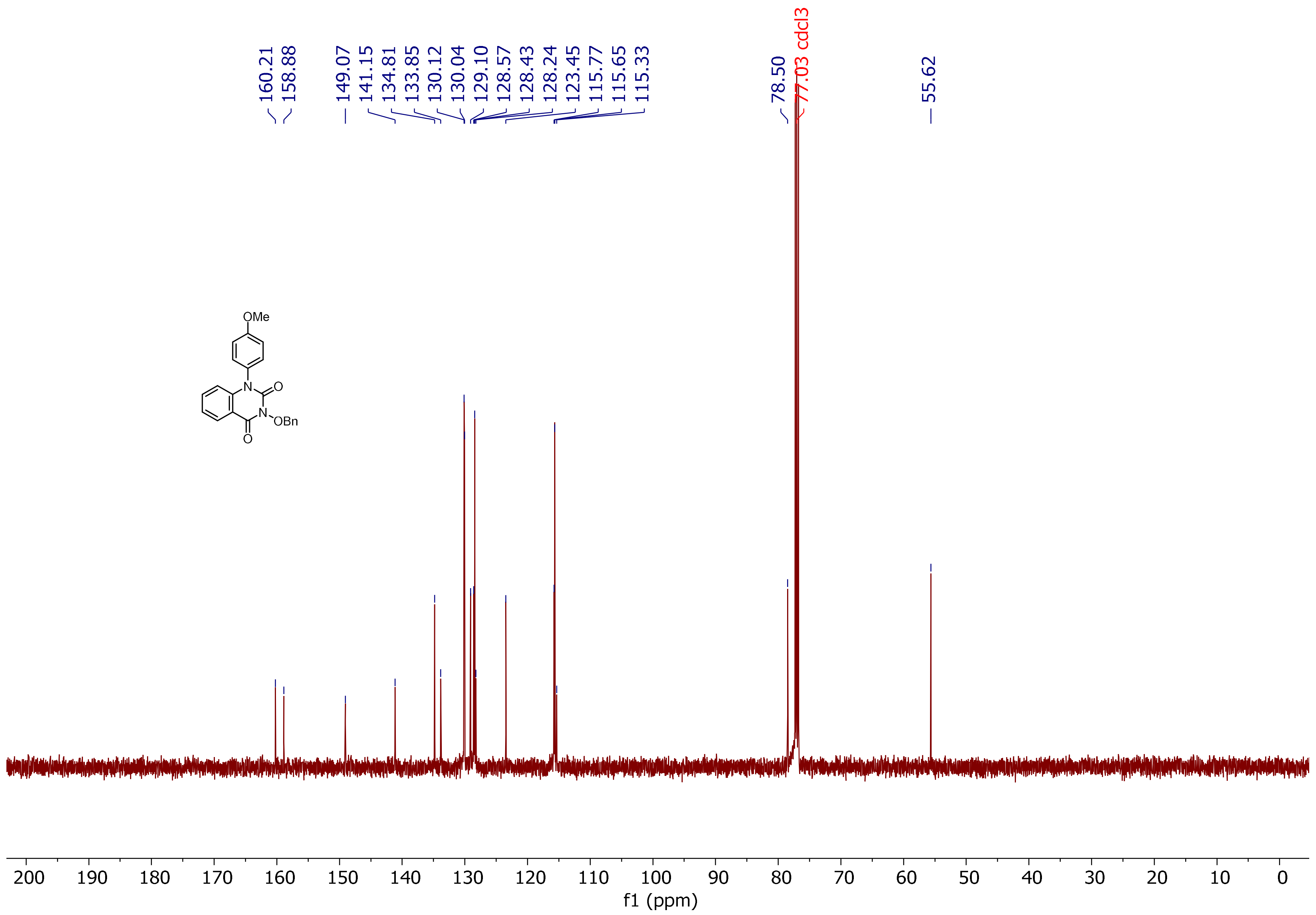

160.21
158.88
149.07
141.15
134.81
133.85
130.12
130.04
129.10
128.57
128.43
128.24
123.45
115.77
115.65
115.33
78.50
77.03 cdcl3
55.62
OMe
N
O
N
OBn
O
200 190 180 170 160 150 140 130 120 110 100 90 80 70 60 50 40 30 20 10 0
f1 (ppm)

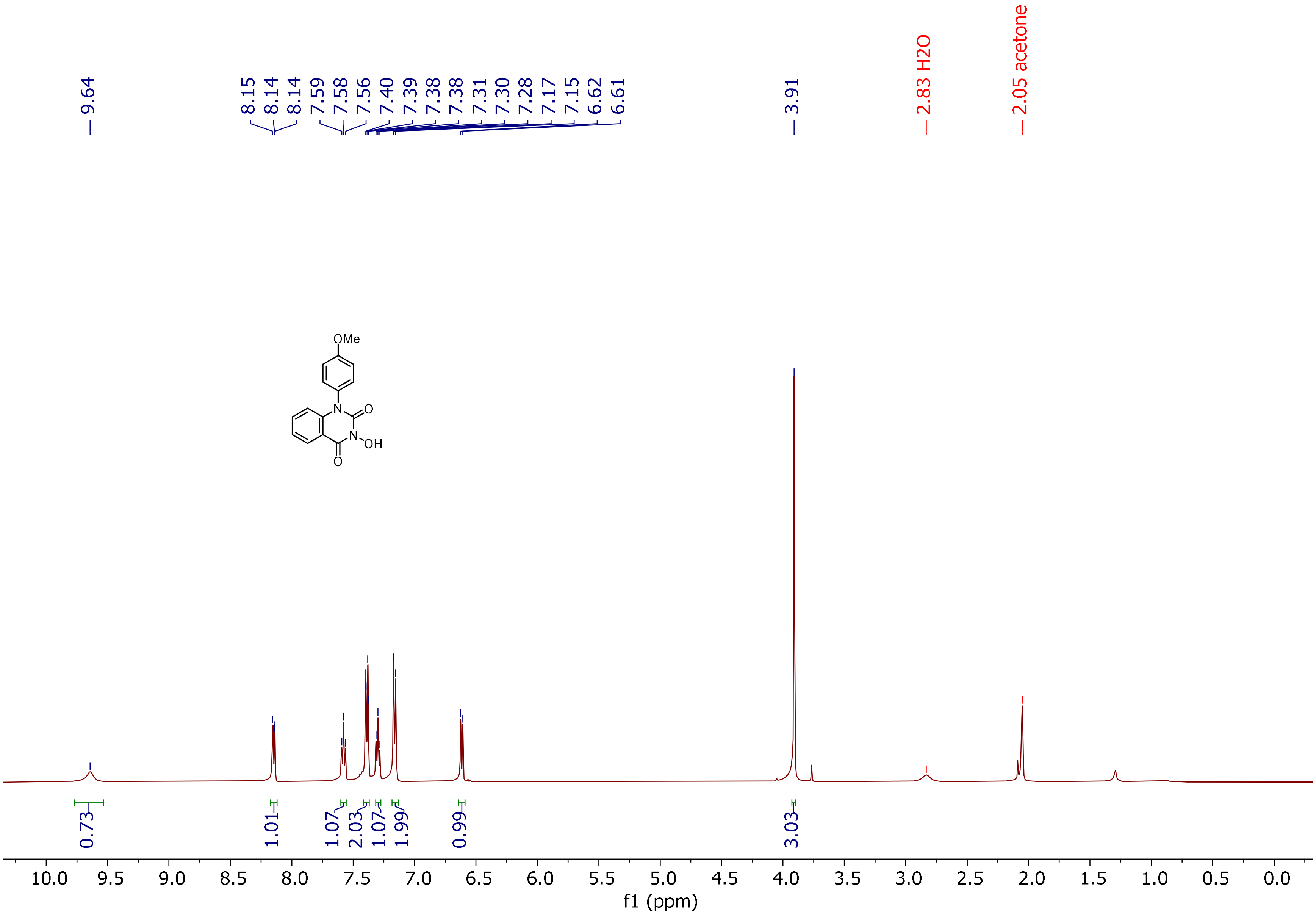

9.64
8.15
8.14
8.14
7.59
7.58
7.56
7.40
7.39
7.38
7.38
7.31
7.30
7.28
7.17
7.15
6.62
6.61
3.91
2.83 H2O
2.05 acetone
OMe
N
O
N
OH
O
0.73
1.01
1.07
2.03
1.07
1.99
0.99
3.03
10.0
9.5
9.0
8.5
8.0
7.5
7.0
6.5
6.0
5.5
5.0
4.5
4.0
3.5
3.0
2.5
2.0
1.5
1.0
0.5
0.0
f1 (ppm)

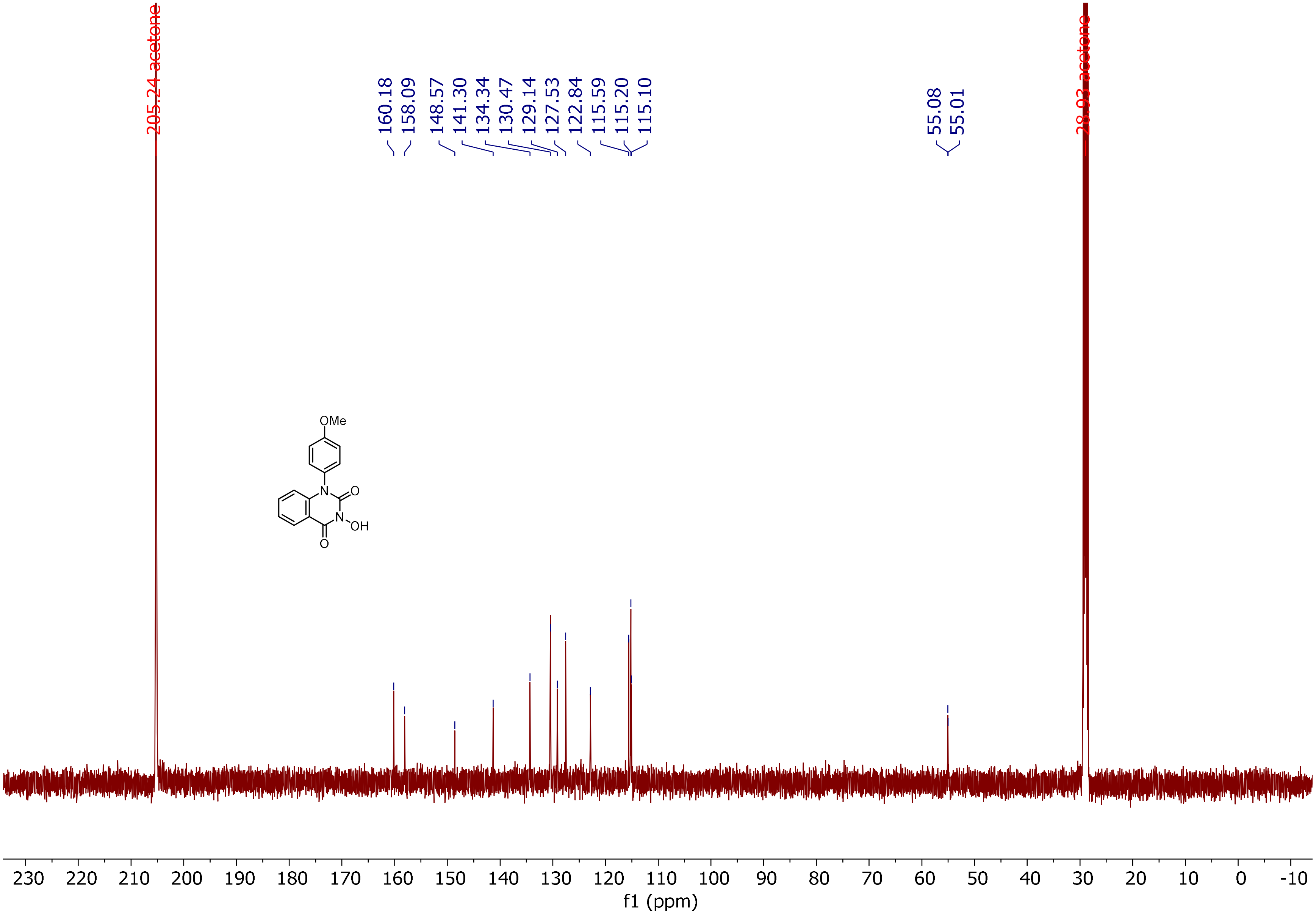
205.24 acetone
160.18
158.09
148.57
141.30
134.34
130.47
129.14
127.53
122.84
115.59
115.20
115.10
55.08
55.01
28.03 acetone
OMe
N
O
N
OH
O
230 220 210 200 190 180 170 160 150 140 130 120 110 100 90 80 70 60 50 40 30 20 10 0 -10
f1 (ppm)

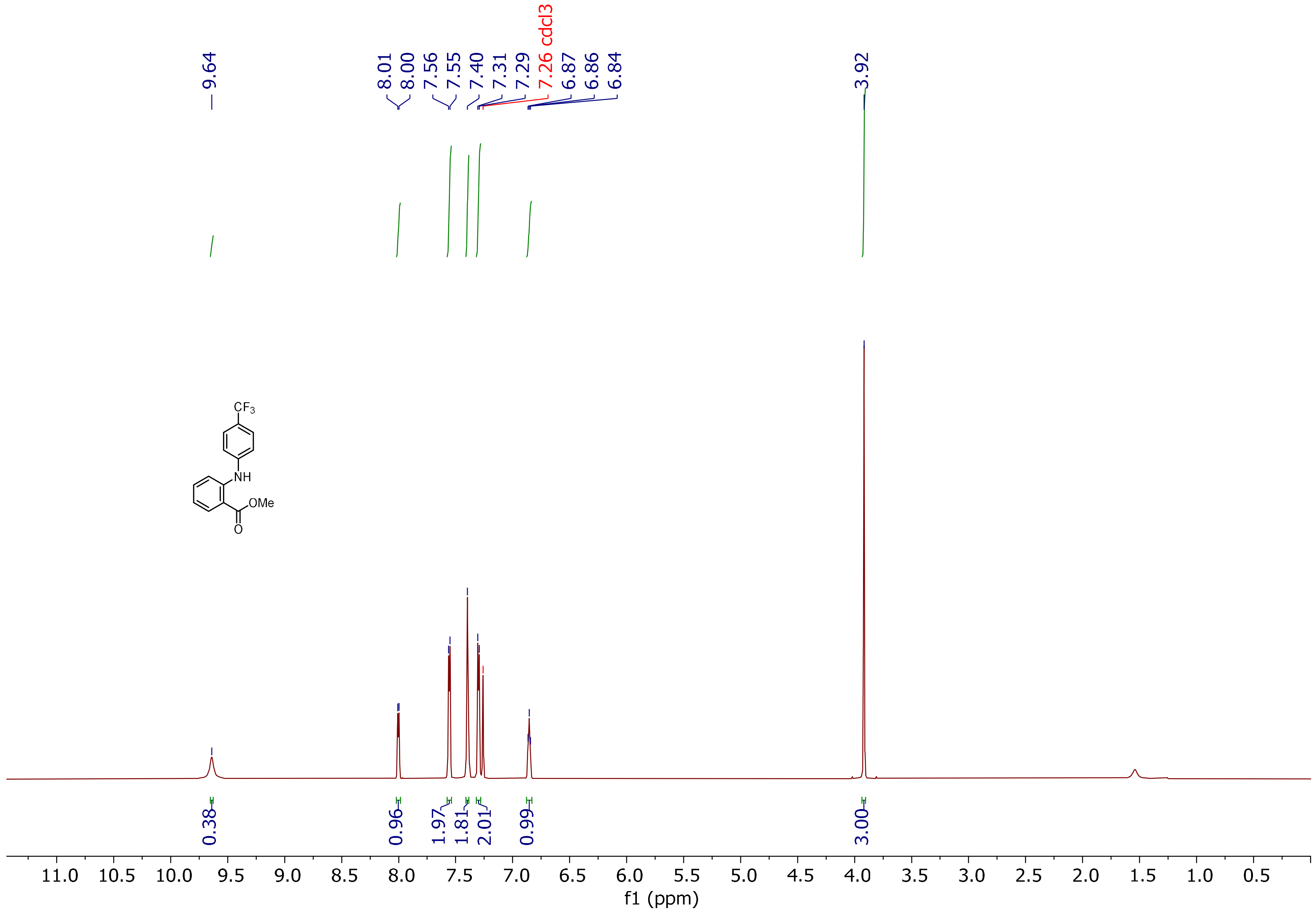
9.64
8.01
8.00
7.56
7.55
7.40
7.31
7.29
7.26 cdcl3
6.87
6.86
6.84
3.92
CF3
NH
OMe
O
0.38
0.96
1.97
1.81
2.01
0.99
3.00
11.0 10.5 10.0 9.5 9.0 8.5 8.0 7.5 7.0 6.5 6.0 5.5 5.0 4.5 4.0 3.5 3.0 2.5 2.0 1.5 1.0 0.5
f1 (ppm)

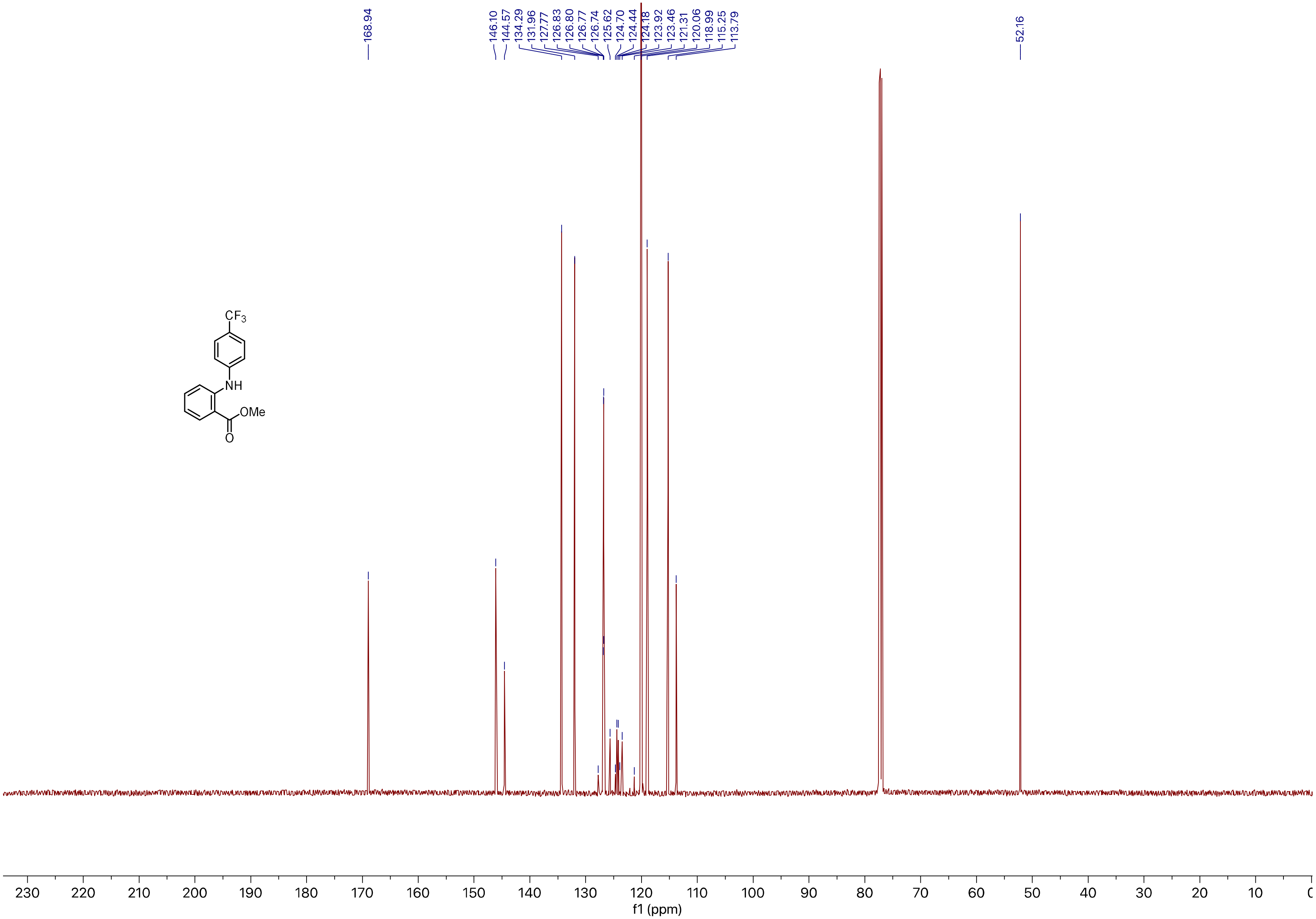
168.94
146.10
144.57
134.29
131.96
127.77
126.83
126.80
126.77
126.74
125.62
124.70
124.44
124.18
123.92
123.46
121.31
120.06
118.99
115.25
113.79
52.16
CF3
NH
OMe
O
230 220 210 200 190 180 170 160 150 140 130 120 110 100 90 80 70 60 50 40 30 20 10
f1 (ppm)

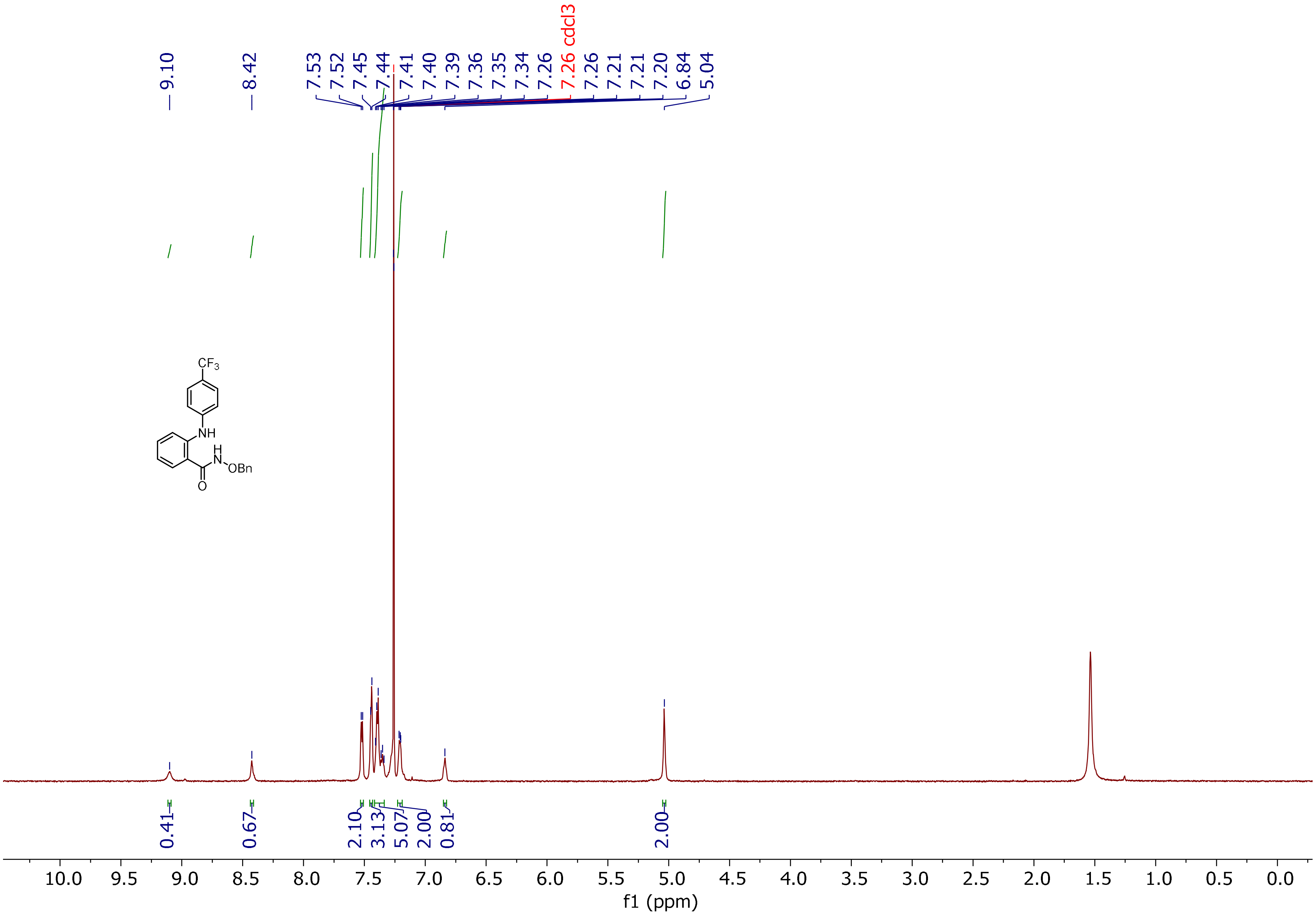

9.10
8.42
7.53
7.52
7.45
7.44
7.41
7.40
7.39
7.36
7.35
7.34
7.26
7.26 cdcl3
7.26
7.21
7.21
7.20
6.84
5.04
CF3
NH
H
N
OBn
O
0.41
0.67
2.10
3.13
5.07
2.00
0.81
2.00
10.0
9.5
9.0
8.5
8.0
7.5
7.0
6.5
6.0
5.5
5.0
4.5
4.0
3.5
3.0
2.5
2.0
1.5
1.0
0.5
0.0
f1 (ppm)

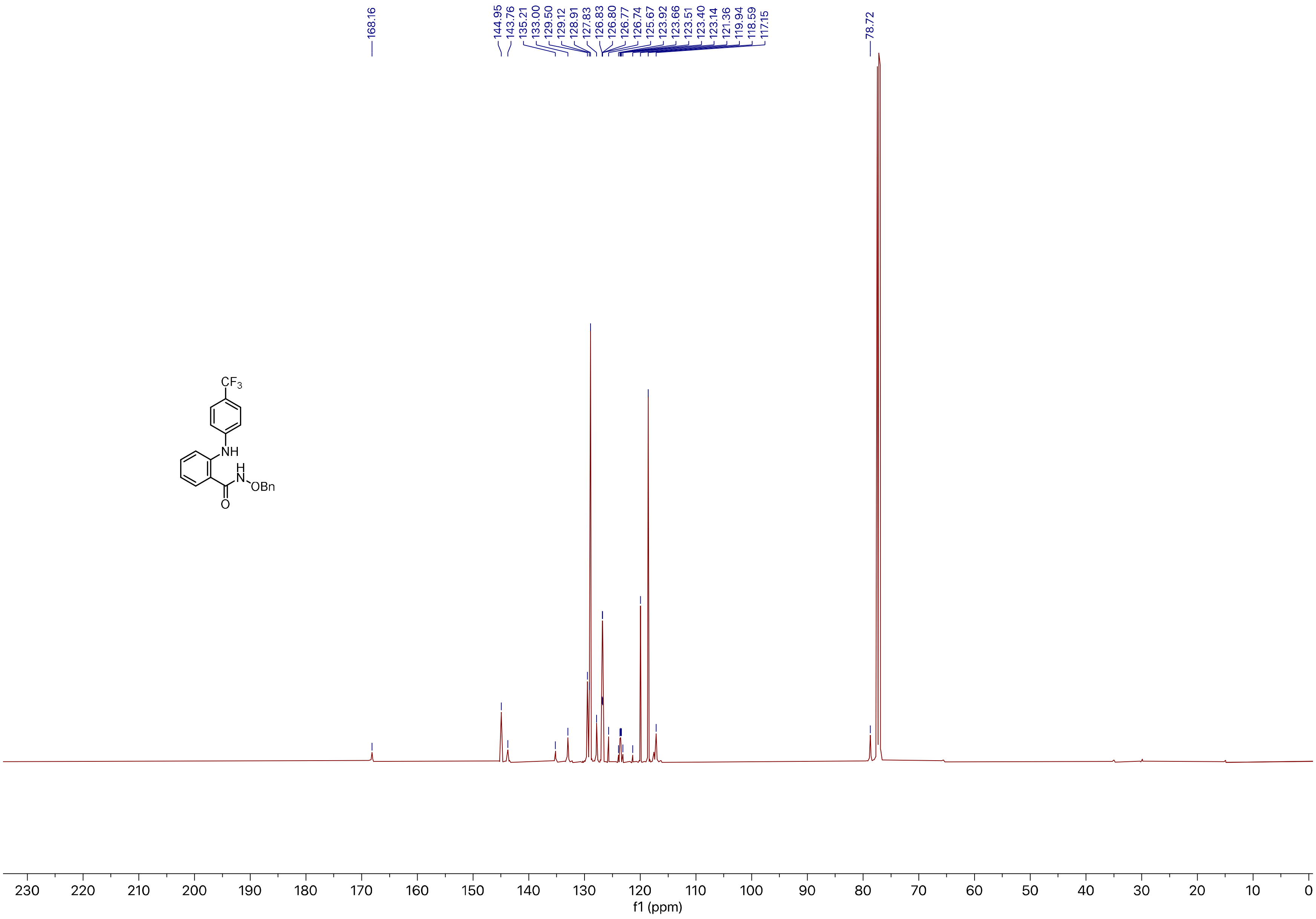
168.16
144.95
143.76
135.21
133.00
129.50
129.12
128.91
127.83
126.83
126.80
126.77
126.74
125.67
123.92
123.66
123.51
123.40
123.14
121.36
119.94
118.59
117.15
78.72
CF3
NH
H
N
OBn
O
230
220
210
200
190
180
170
160
150
140
130
120
110
100
90
80
70
60
50
40
30
20
10
0
f1 (ppm)

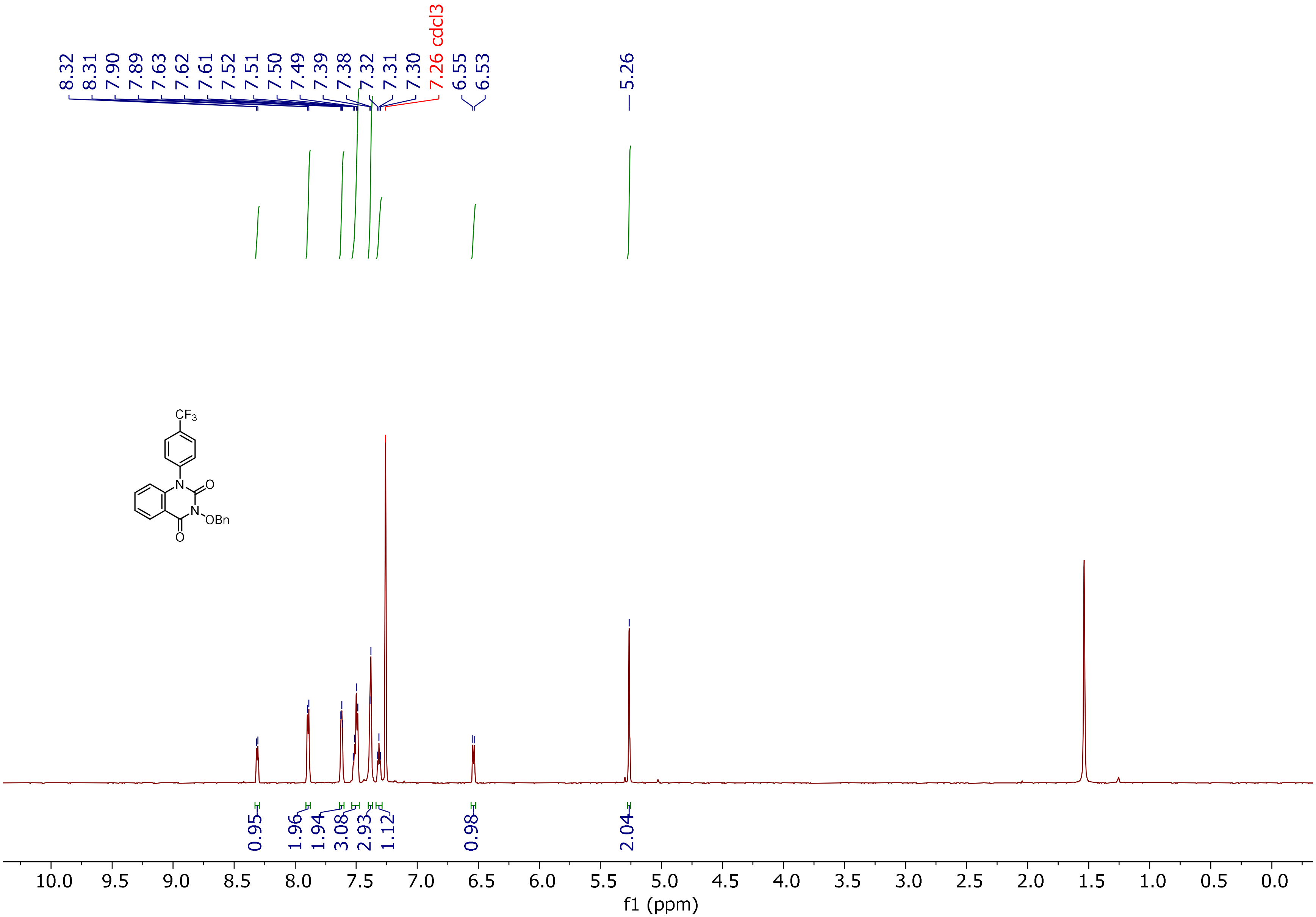

8.32
8.31
7.90
7.89
7.63
7.62
7.61
7.52
7.51
7.50
7.49
7.39
7.38
7.32
7.31
7.30
7.26 cdcl3
6.55
6.53
5.26
CF3
N
O
N
OBn
O
0.95
1.96
1.94
3.08
2.93
1.12
0.98
2.04
10.0
9.5
9.0
8.5
8.0
7.5
7.0
6.5
6.0
5.5
5.0
4.5
4.0
3.5
3.0
2.5
2.0
1.5
1.0
0.5
0.0
f1 (ppm)

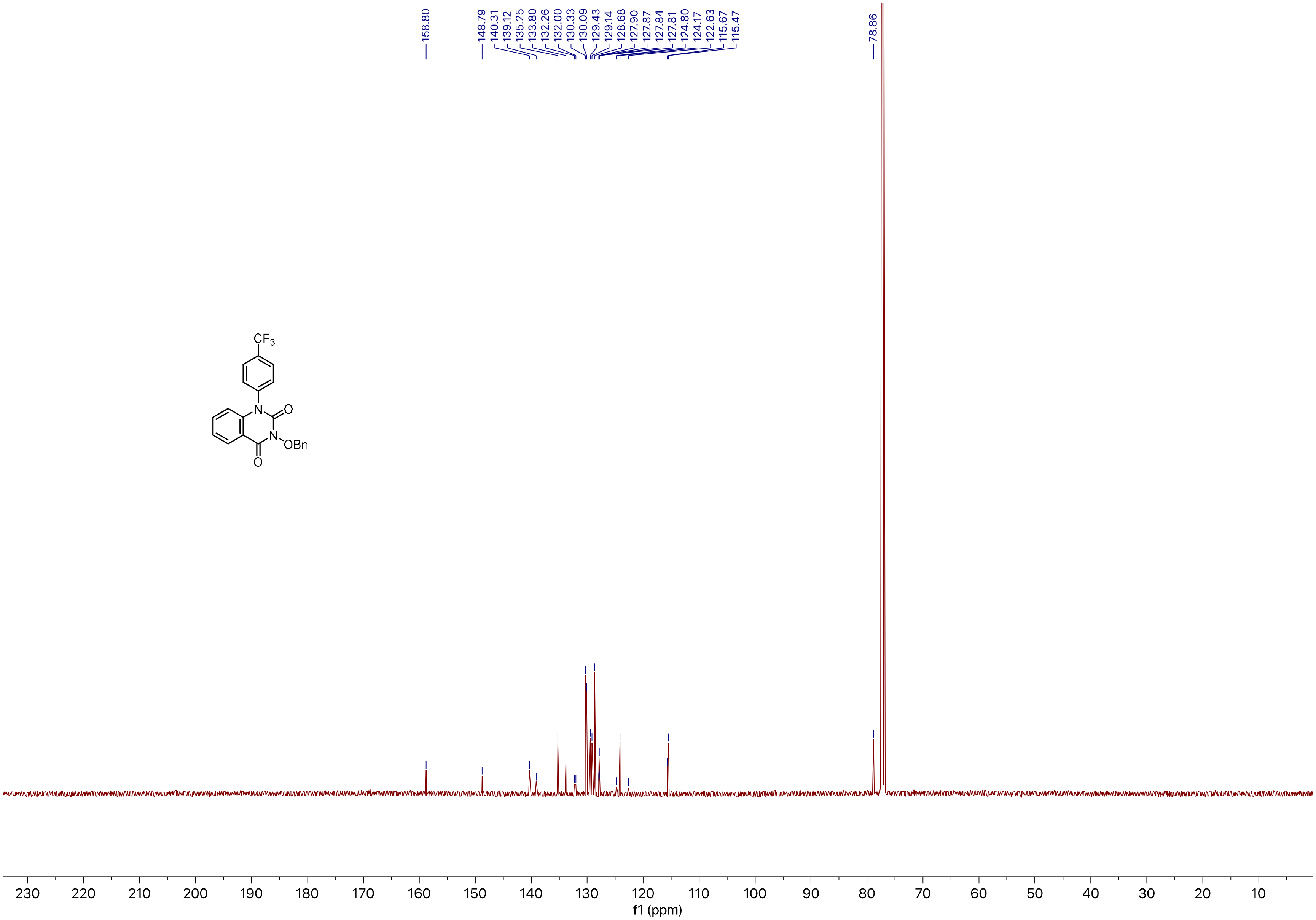
158.80
148.79
140.31
139.12
135.25
133.80
132.26
132.00
130.33
130.09
129.43
129.14
128.68
127.90
127.87
127.84
127.81
124.80
124.17
122.63
115.67
115.47
78.86
CF3
N
O
N
OBn
O
230 220 210 200 190 180 170 160 150 140 130 120 110 100 90 80 70 60 50 40 30 20 10
f1 (ppm)

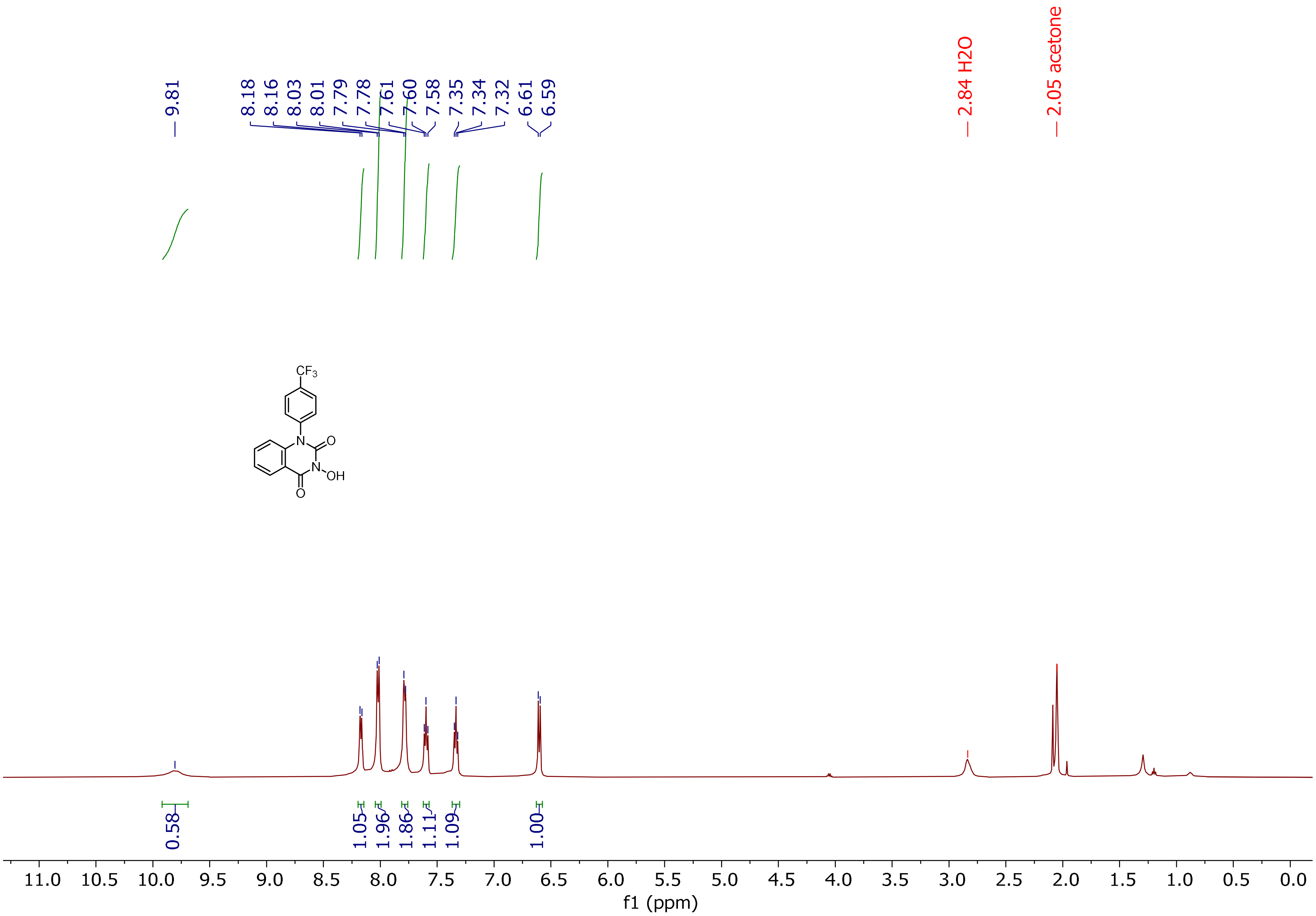
9.81
8.18
8.16
8.03
8.01
7.79
7.78
7.61
7.60
7.58
7.35
7.34
7.32
6.61
6.59
2.84 H2O
2.05 acetone
CF3
N
O
N
OH
O
0.58
1.05
1.96
1.86
1.11
1.09
1.00
f1 (ppm)

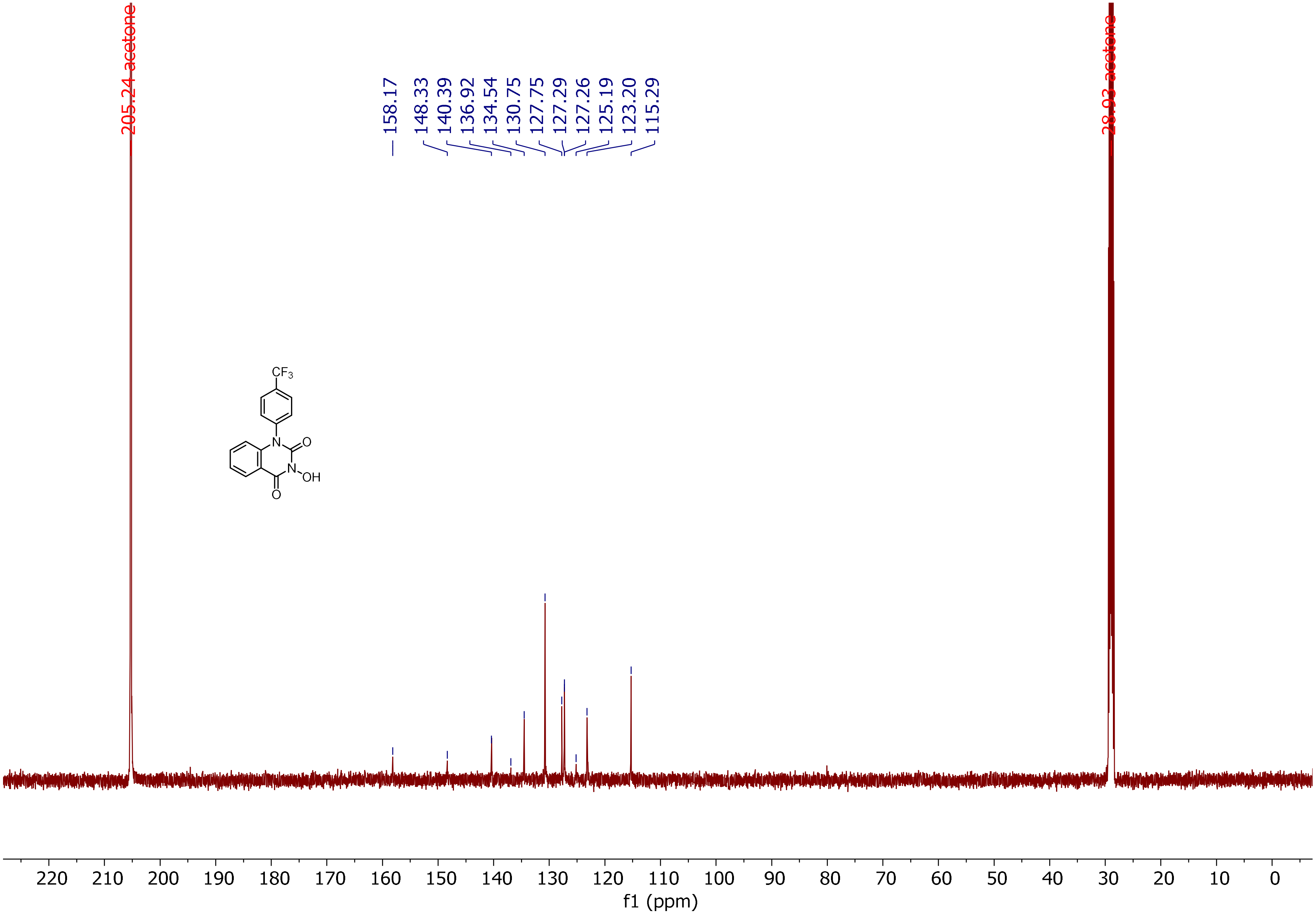
205.24 acetone
158.17
148.33
140.39
136.92
134.54
130.75
127.75
127.29
127.26
125.19
123.20
115.29
28.93 acetone
CF3
N
O
N
OH
O
220 210 200 190 180 170 160 150 140 130 120 110 100 90 80 70 60 50 40 30 20 10 0
f1 (ppm)

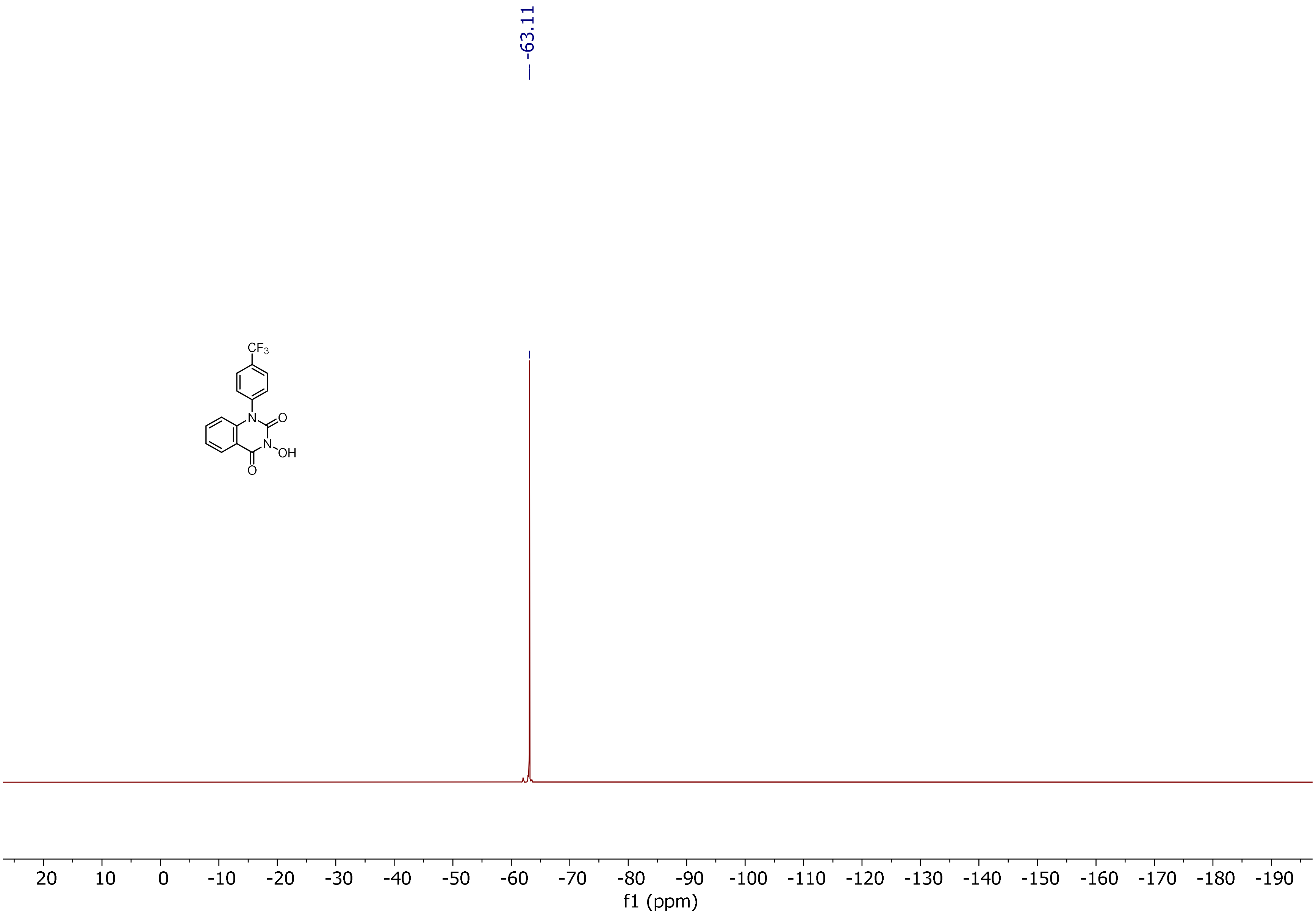
-63.11
CF3
N
O
N
OH
O
20 10 0 -10 -20 -30 -40 -50 -60 -70 -80 -90 -100 -110 -120 -130 -140 -150 -160 -170 -180 -190
f1 (ppm)

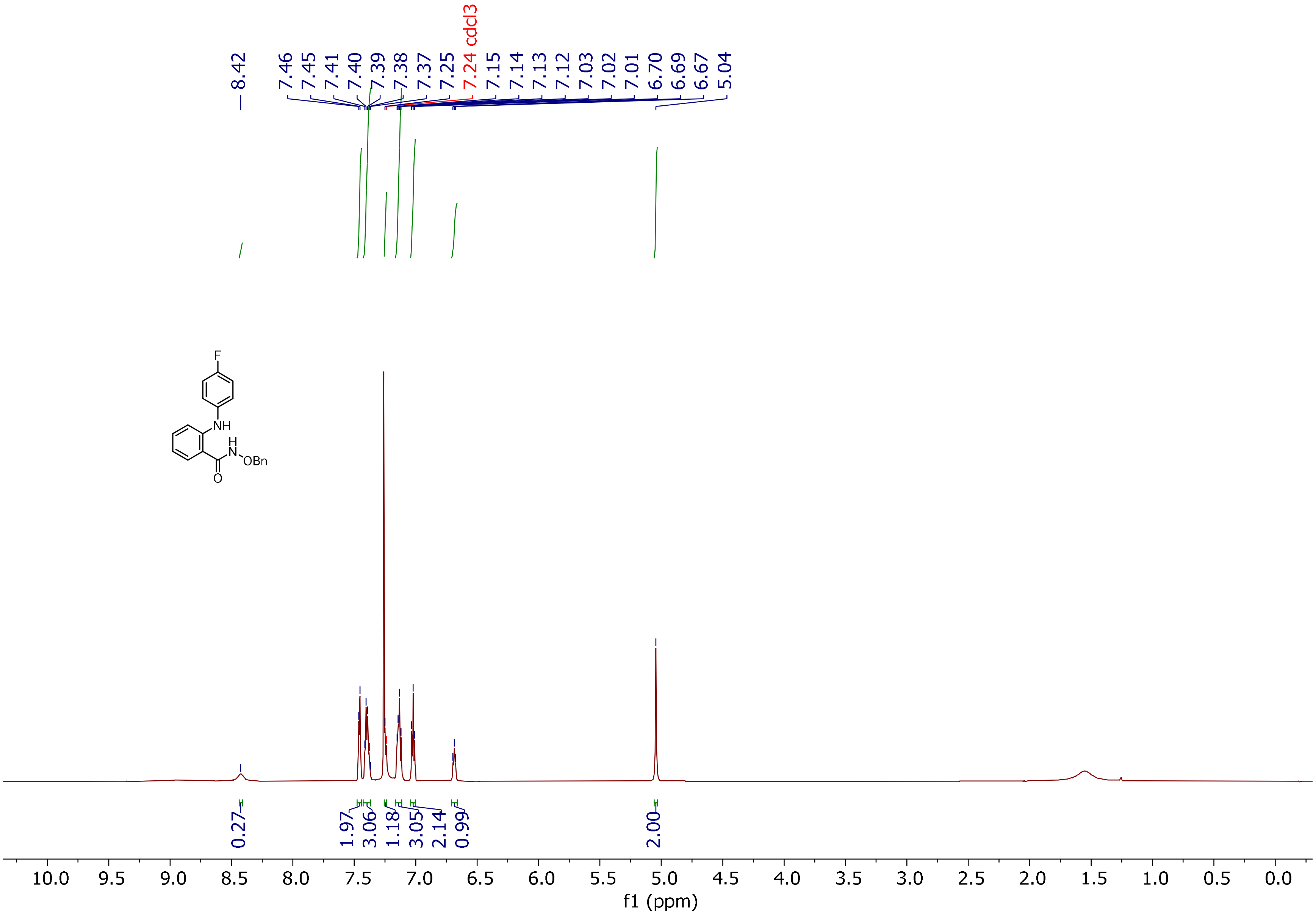
8.42
7.46
7.45
7.41
7.40
7.39
7.38
7.37
7.25
7.24 cdcl3
7.15
7.14
7.13
7.12
7.03
7.02
7.01
6.70
6.69
6.67
5.04
F
NH
H
N
OBn
O
0.27
1.97
3.06
1.18
3.05
2.14
0.99
2.00
10.0
9.5
9.0
8.5
8.0
7.5
7.0
6.5
6.0
5.5
5.0
4.5
4.0
3.5
3.0
2.5
2.0
1.5
1.0
0.5
0.0
f1 (ppm)

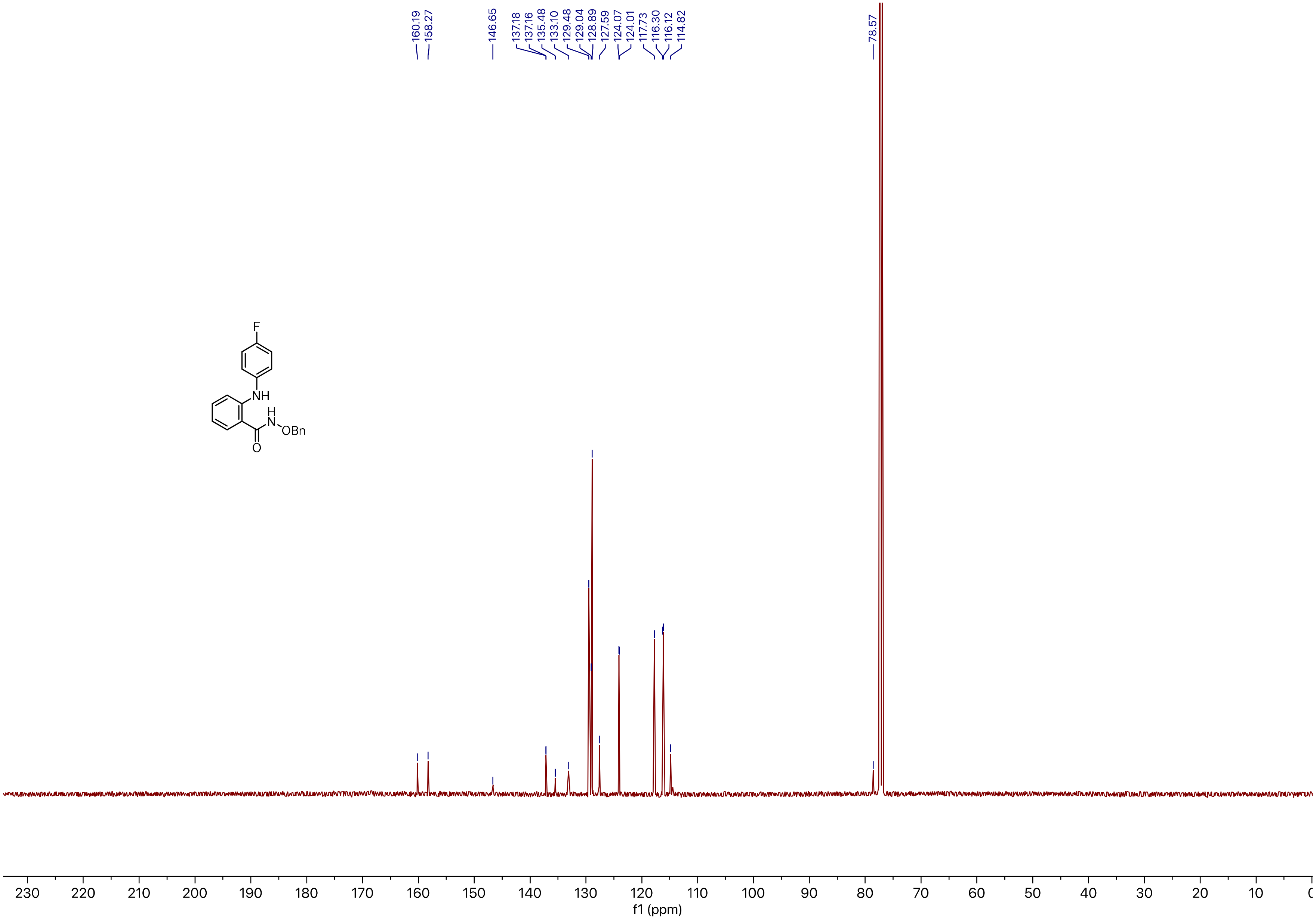
160.19
158.27
146.65
137.18
137.16
135.48
133.10
129.48
129.04
128.89
127.59
124.07
124.01
117.73
116.30
116.12
114.82
78.57
F
NH
H
N
OBn
O
230 220 210 200 190 180 170 160 150 140 130 120 110 100 90 80 70 60 50 40 30 20 10
f1 (ppm)

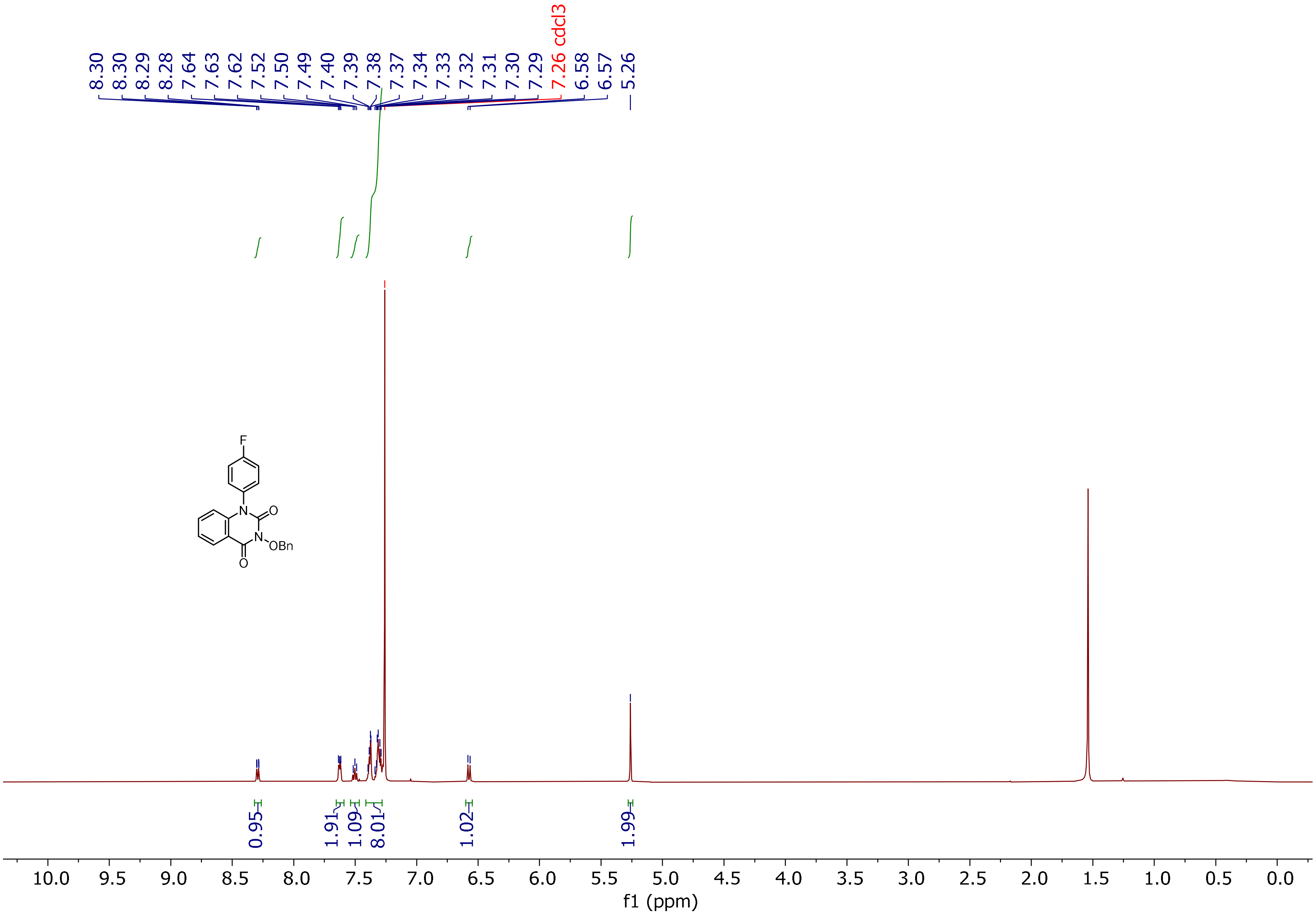

8.30
8.30
8.29
8.28
7.64
7.63
7.62
7.52
7.50
7.49
7.40
7.39
7.38
7.37
7.34
7.33
7.32
7.31
7.30
7.29
7.26 cdcl3
6.58
6.57
5.26
F
N
O
N
OBn
O
0.95
1.91
1.09
8.01
1.02
1.99
10.0
9.5
9.0
8.5
8.0
7.5
7.0
6.5
6.0
5.5
5.0
4.5
4.0
3.5
3.0
2.5
2.0
1.5
1.0
0.5
0.0
f1 (ppm)

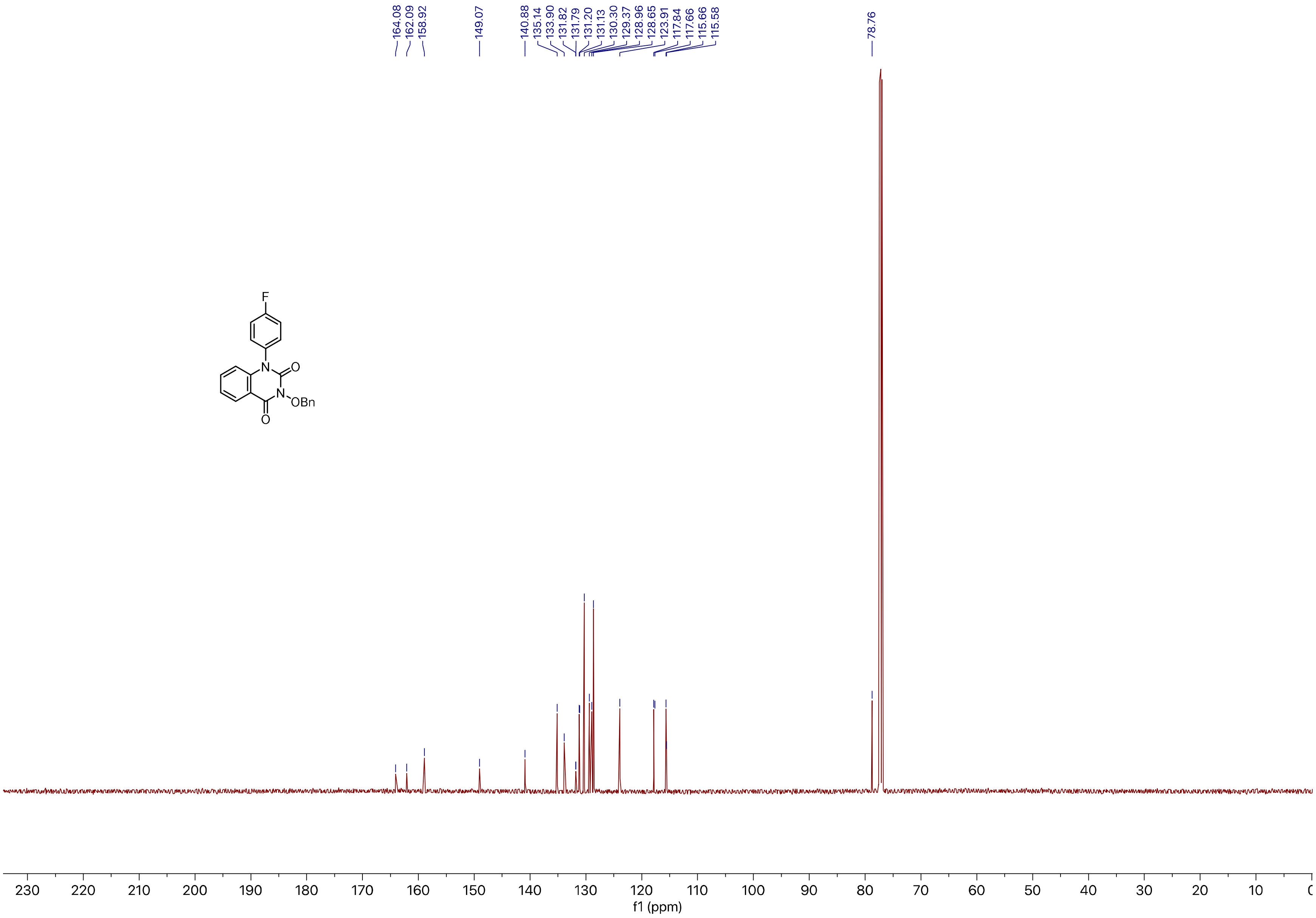
164.08
162.09
158.92
149.07
140.88
135.14
133.90
131.82
131.79
131.20
131.13
130.30
129.37
128.96
128.65
123.91
117.84
117.66
115.66
115.58
78.76
F
N
O
N
OBn
O
230 220 210 200 190 180 170 160 150 140 130 120 110 100 90 80 70 60 50 40 30 20 10
f1 (ppm)

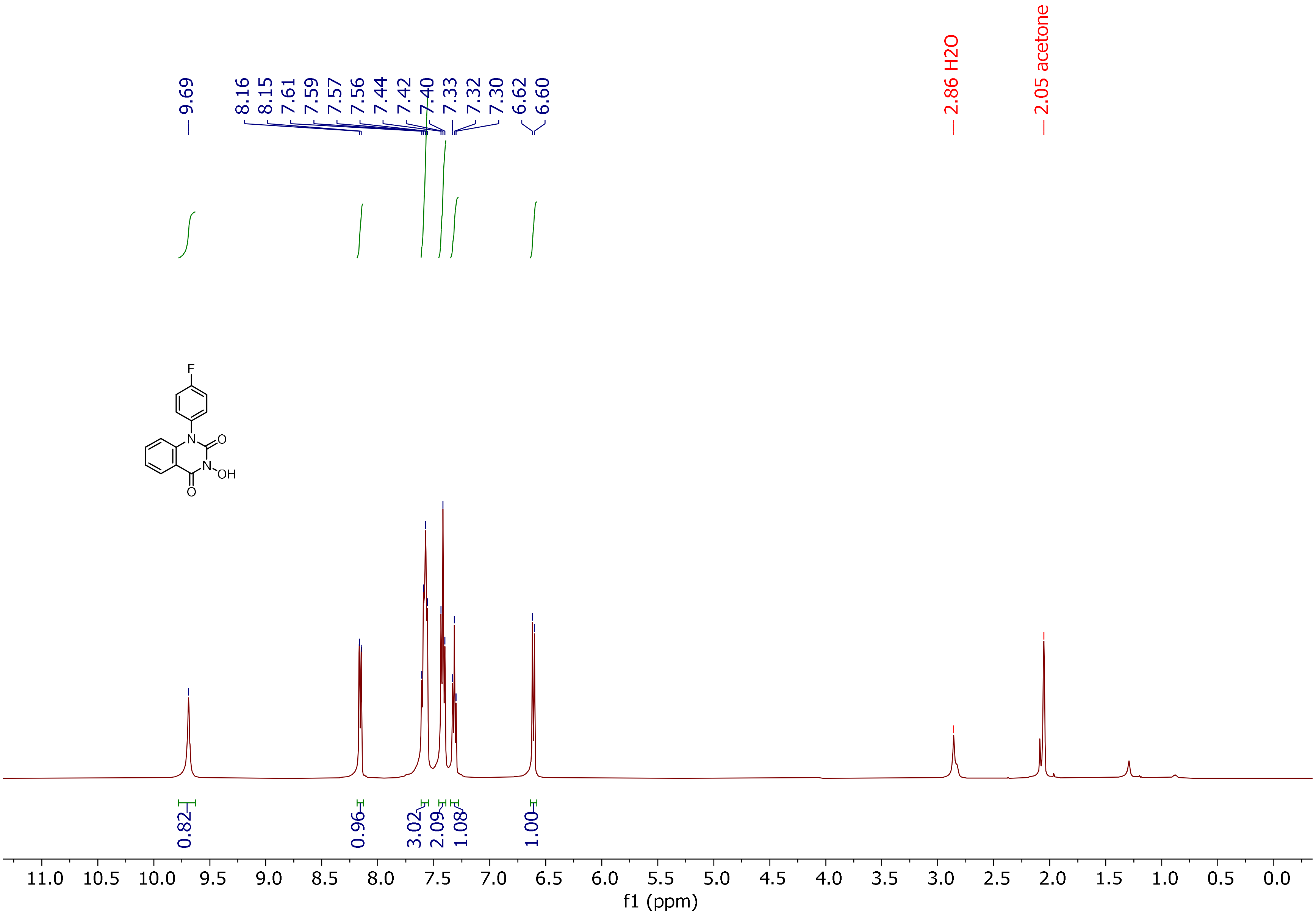

9.69
8.16
8.15
7.61
7.59
7.57
7.56
7.44
7.42
7.40
7.33
7.32
7.30
6.62
6.60
2.86 H2O
2.05 acetone
F
N
O
N
OH
O
0.82
0.96
3.02
2.09
1.08
1.00
11.0 10.5 10.0 9.5 9.0 8.5 8.0 7.5 7.0 6.5 6.0 5.5 5.0 4.5 4.0 3.5 3.0 2.5 2.0 1.5 1.0 0.5 0.0
f1 (ppm)

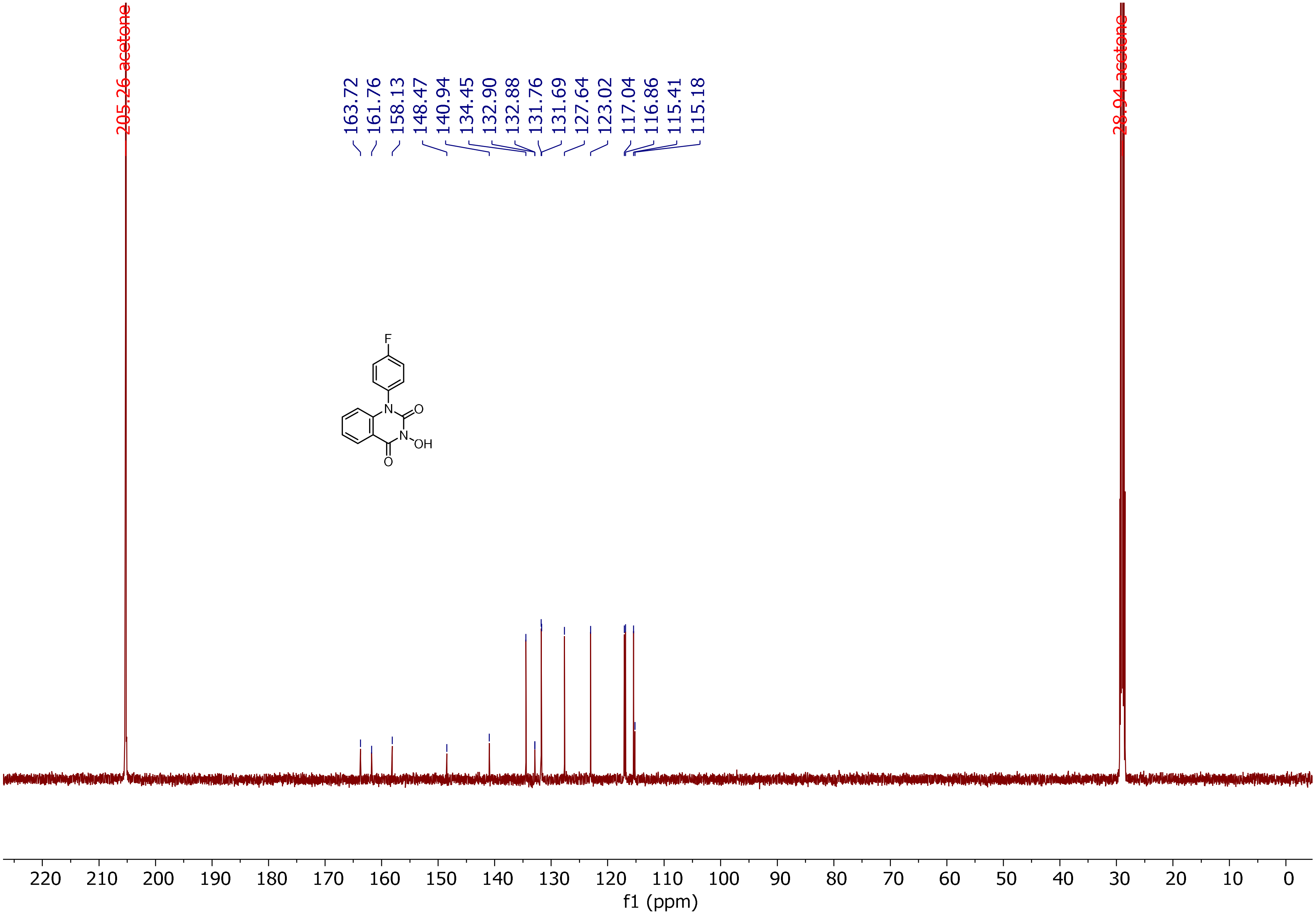
205.26 acetone
163.72
161.76
158.13
148.47
140.94
134.45
132.90
132.88
131.76
131.69
127.64
123.02
117.04
116.86
115.41
115.18
28.94 acetone
F
N
O
N
OH
O
220 210 200 190 180 170 160 150 140 130 120 110 100 90 80 70 60 50 40 30 20 10 0
f1 (ppm)

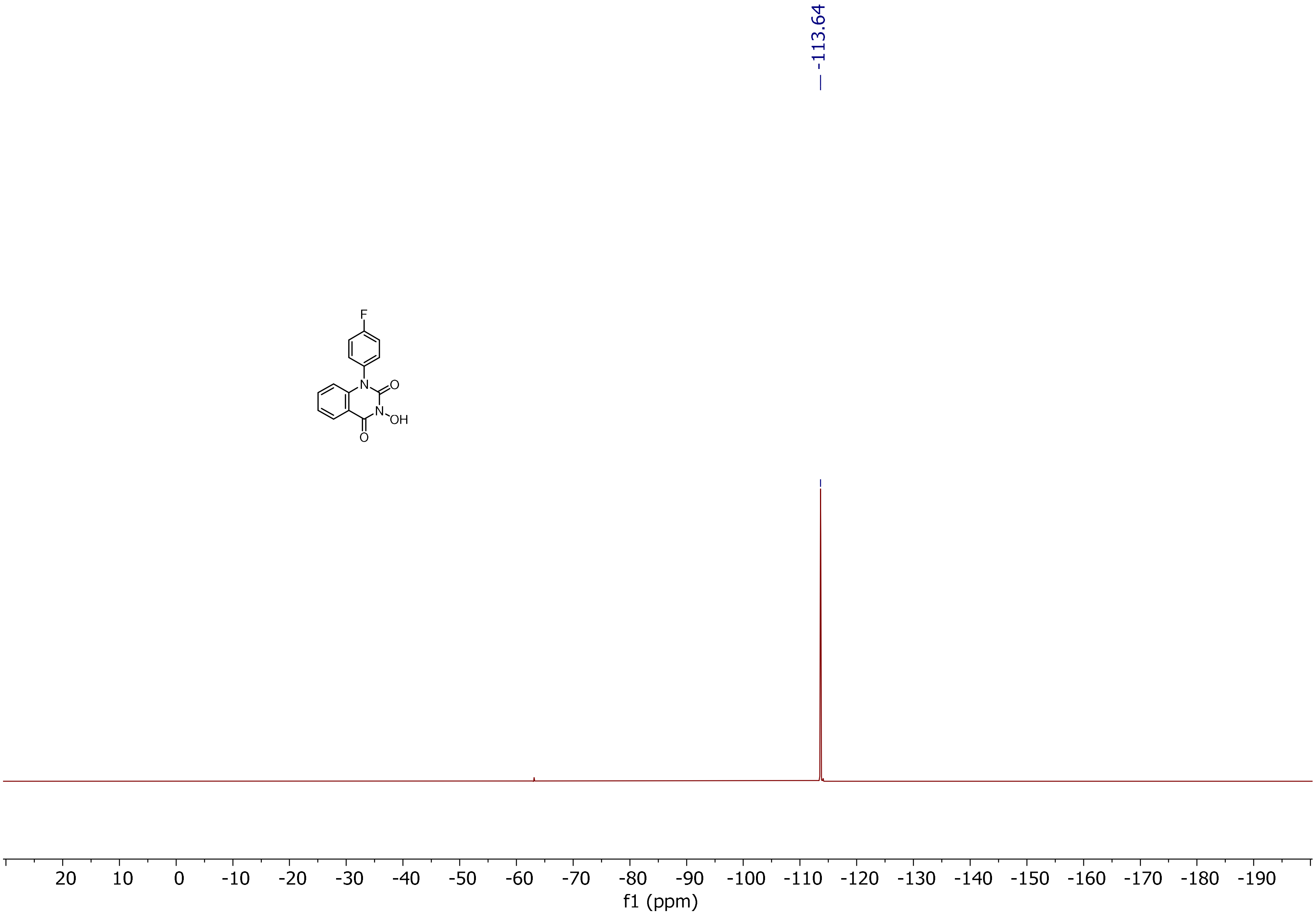
-113.64
F
N
O
N
OH
O
20 10 0 -10 -20 -30 -40 -50 -60 -70 -80 -90 -100 -110 -120 -130 -140 -150 -160 -170 -180 -190
f1 (ppm)

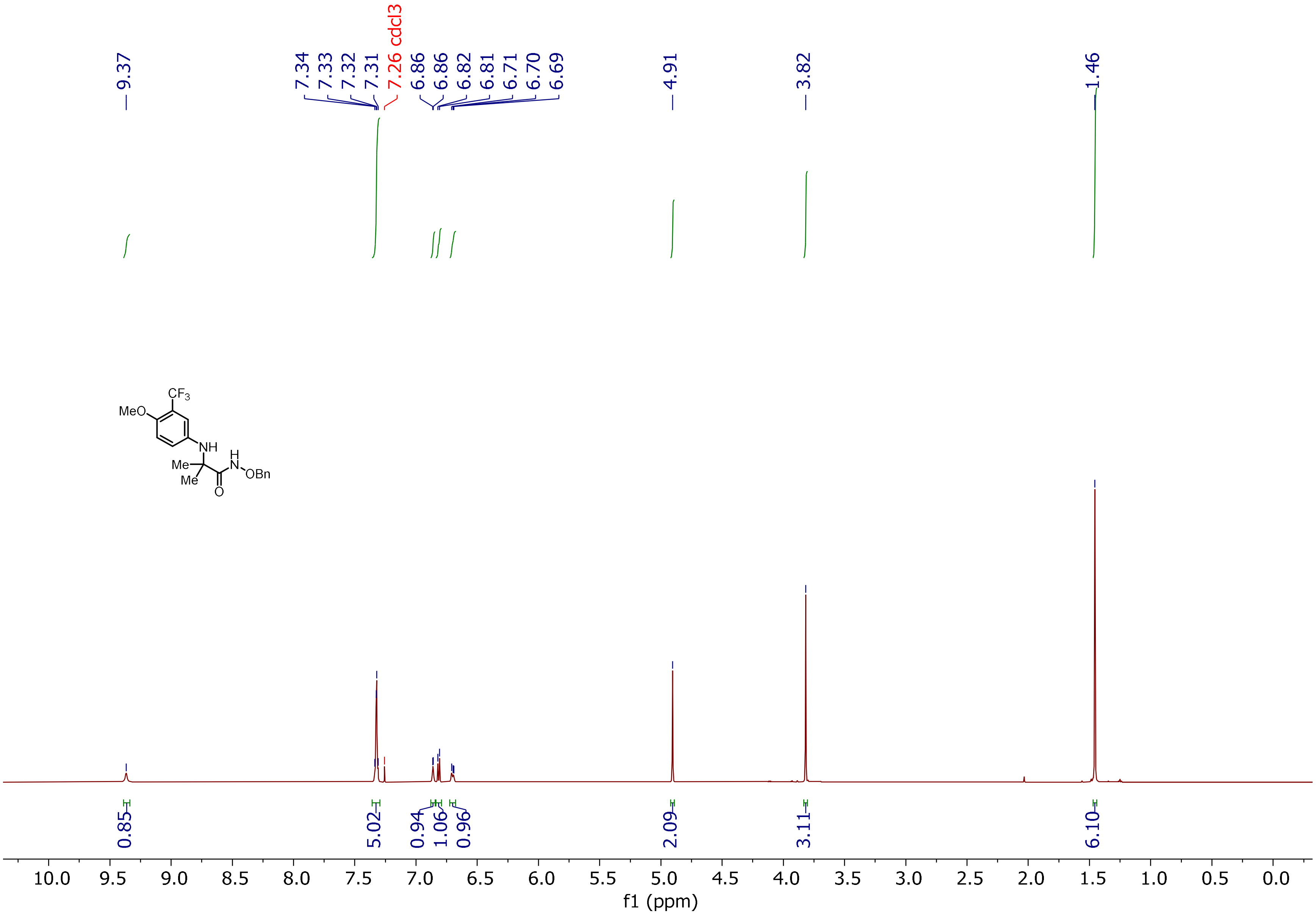

9.37
7.34
7.33
7.32
7.31
7.26 cdcl3
6.86
6.86
6.82
6.81
6.71
6.70
6.69
4.91
3.82
1.46
CF3
MeO
NH
H
N
OBn
Me
Me
O
0.85
5.02
0.94
1.06
0.96
2.09
3.11
6.10
10.0
9.5
9.0
8.5
8.0
7.5
7.0
6.5
6.0
5.5
5.0
4.5
4.0
3.5
3.0
2.5
2.0
1.5
1.0
0.5
0.0
f1 (ppm)

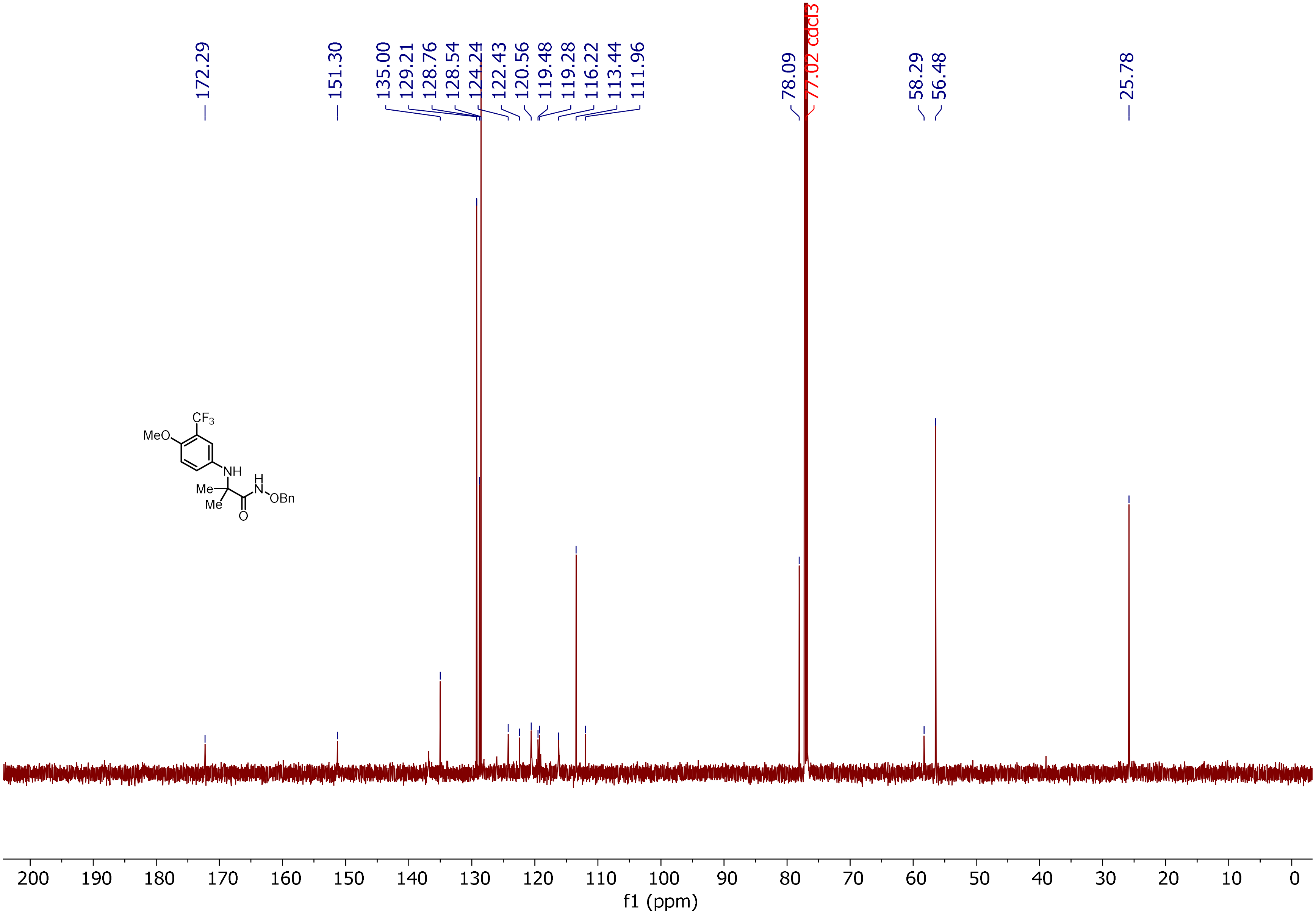

172.29
151.30
135.00
129.21
128.76
128.54
124.24
122.43
120.56
119.48
119.28
116.22
113.44
111.96
78.09
77.02 cdcl3
58.29
56.48
25.78
CF_3
MeO
NH
Me
Me
H
N
OBn
O
200 190 180 170 160 150 140 130 120 110 100 90 80 70 60 50 40 30 20 10 0
f1 (ppm)

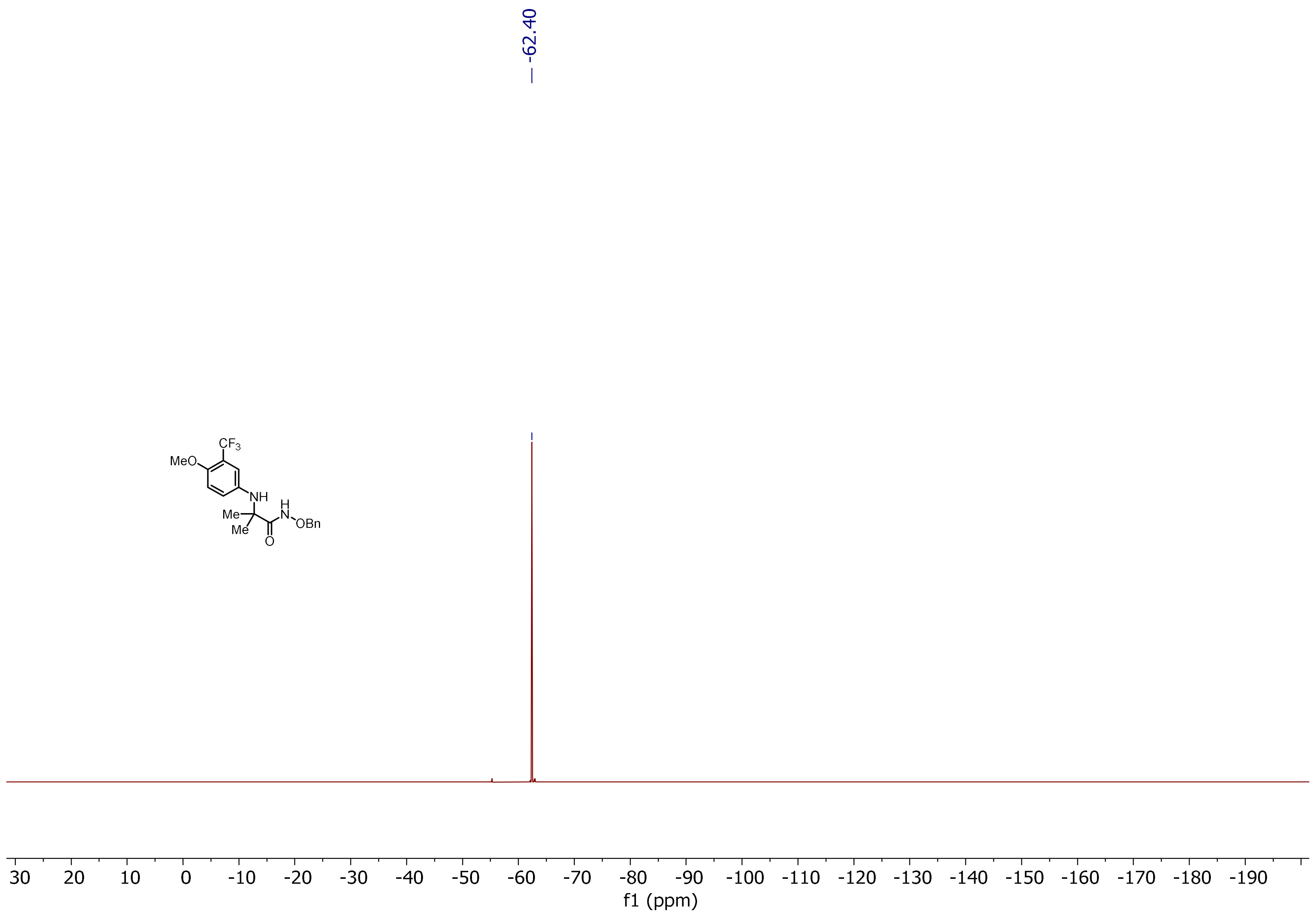

-62.40
MeO
CF3
NH
Me
Me
H
N
OBn
O
30 20 10 0 -10 -20 -30 -40 -50 -60 -70 -80 -90 -100 -110 -120 -130 -140 -150 -160 -170 -180 -190
f1 (ppm)

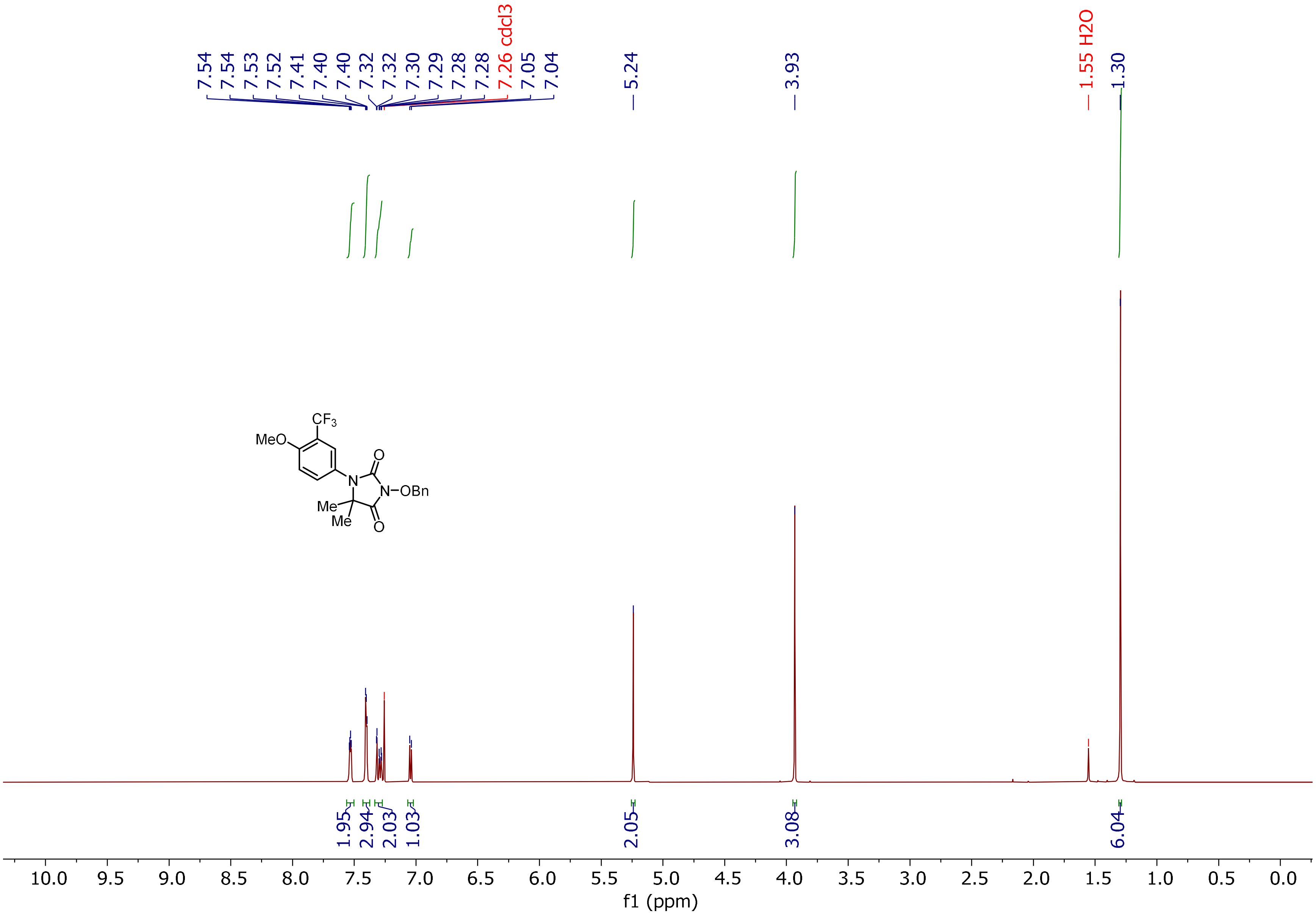
7.54
7.54
7.53
7.52
7.41
7.40
7.40
7.32
7.32
7.30
7.29
7.28
7.28
7.26 cdcl3
7.05
7.04
5.24
3.93
1.55 H2O
1.30
CF3
MeO
N
O
N-OBn
Me
Me
O
1.95
2.94
2.03
1.03
2.05
3.08
6.04
10.0
9.5
9.0
8.5
8.0
7.5
7.0
6.5
6.0
5.5
5.0
4.5
4.0
3.5
3.0
2.5
2.0
1.5
1.0
0.5
0.0
f1 (ppm)

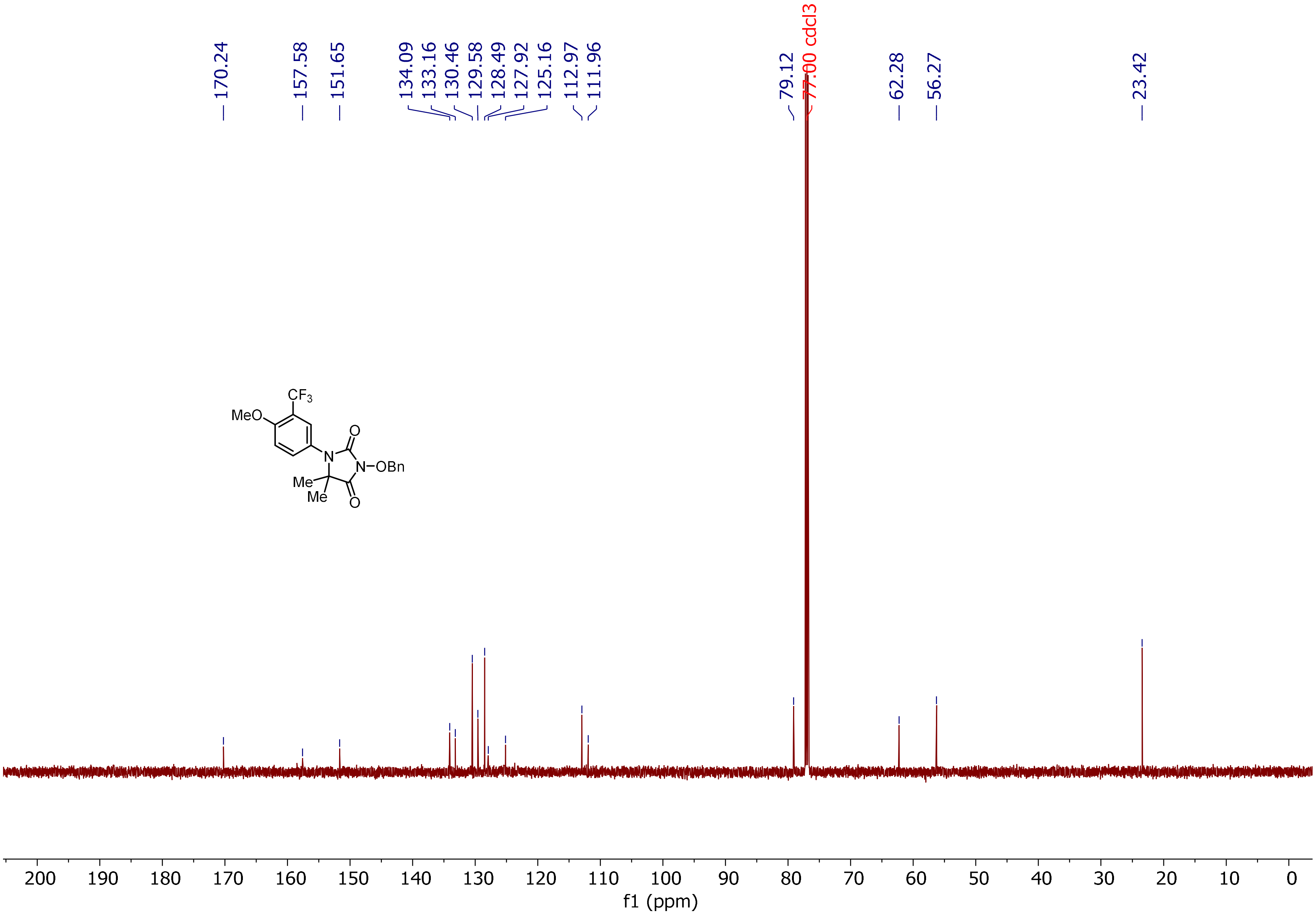
170.24
157.58
151.65
134.09
133.16
130.46
129.58
128.49
127.92
125.16
112.97
111.96
79.12
77.00 cdcl3
62.28
56.27
23.42
CF3
MeO
O
N
N–OBn
Me
Me
O
200 190 180 170 160 150 140 130 120 110 100 90 80 70 60 50 40 30 20 10 0
f1 (ppm)

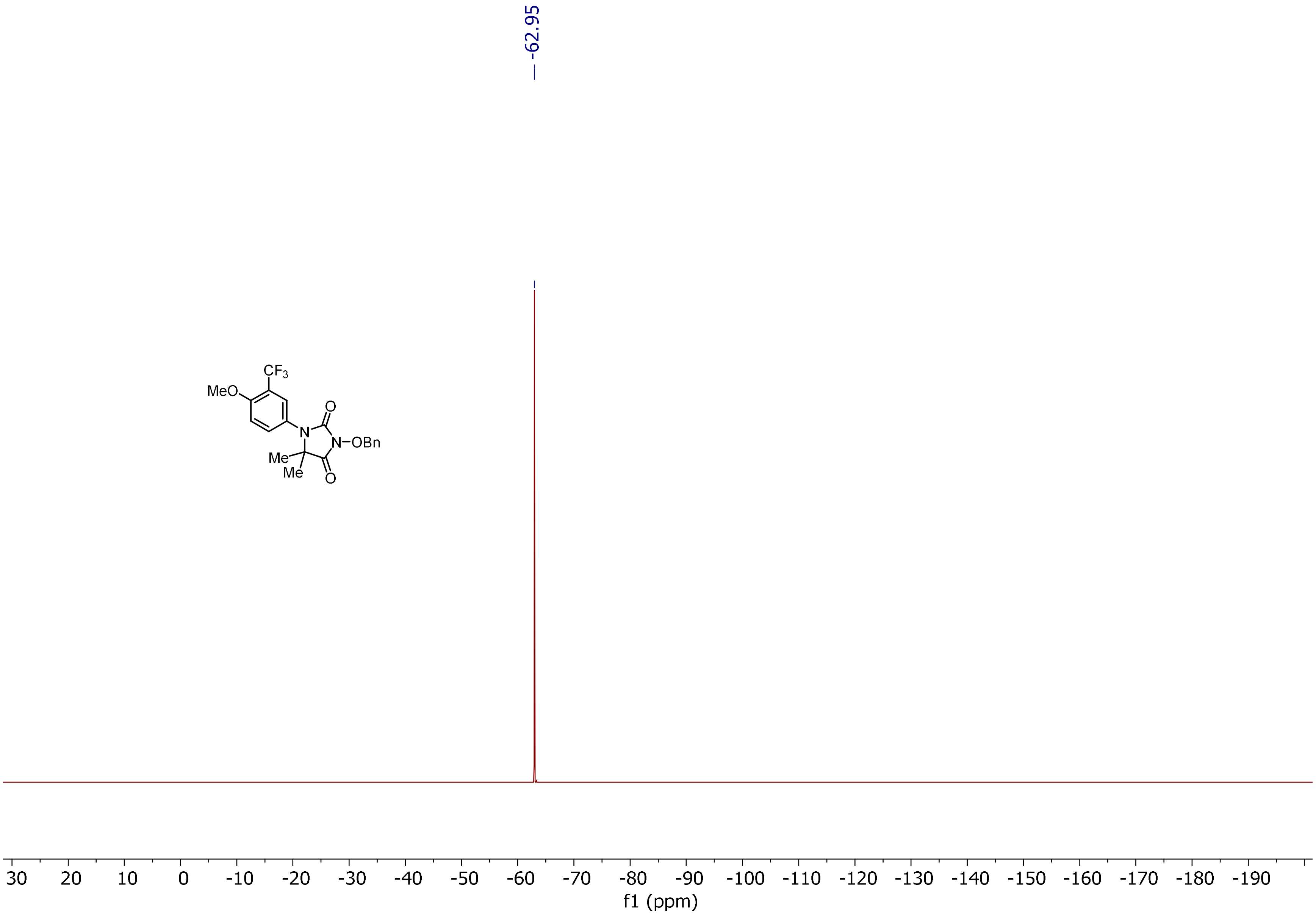
-62.95
CF3
MeO
O
N
N–OBn
Me
Me
O
30 20 10 0 -10 -20 -30 -40 -50 -60 -70 -80 -90 -100 -110 -120 -130 -140 -150 -160 -170 -180 -190
f1 (ppm)

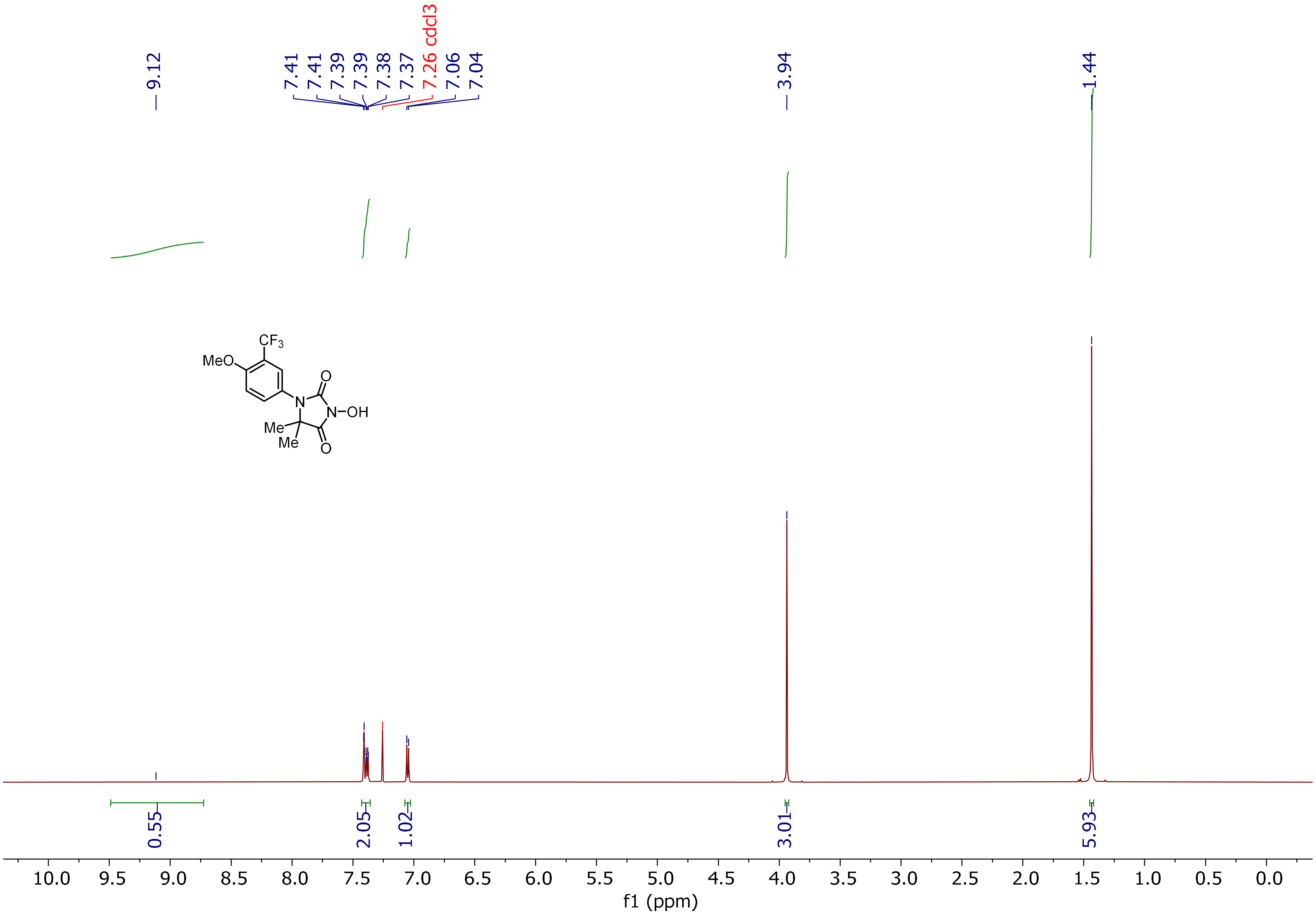

9.12
7.41
7.41
7.39
7.39
7.38
7.37
7.26 cdcl3
7.06
7.04
3.94
1.44
CF3
MeO
N
N-OH
O
O
Me
Me
0.55
2.05
1.02
3.01
5.93
10.0
9.5
9.0
8.5
8.0
7.5
7.0
6.5
6.0
5.5
5.0
4.5
4.0
3.5
3.0
2.5
2.0
1.5
1.0
0.5
0.0
f1 (ppm)

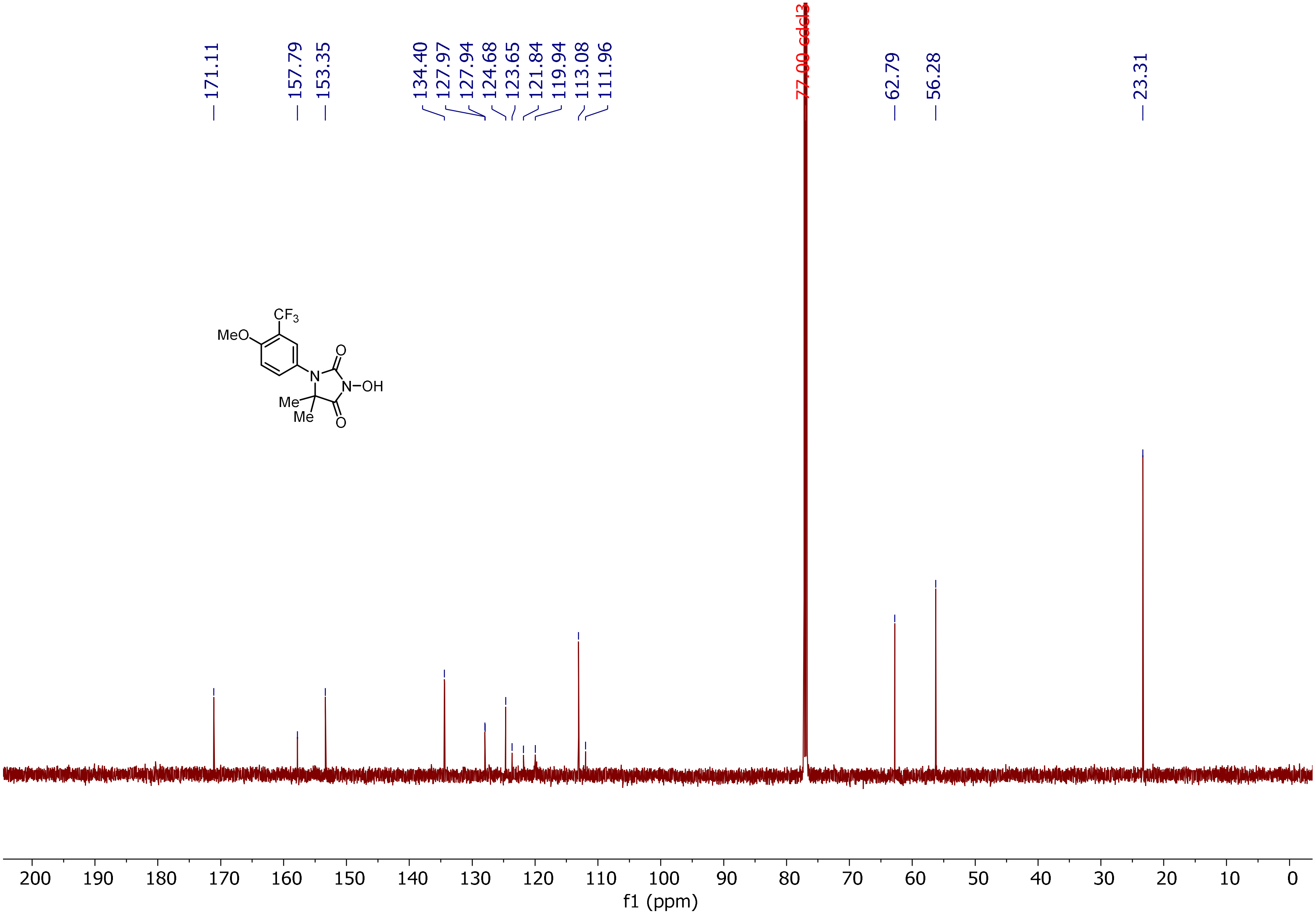
171.11
157.79
153.35
134.40
127.97
127.94
124.68
123.65
121.84
119.94
113.08
111.96
77.00 cdcl3
62.79
56.28
23.31
CF3
MeO
N
O
N-OH
Me
Me
O
200 190 180 170 160 150 140 130 120 110 100 90 80 70 60 50 40 30 20 10 0
f1 (ppm)

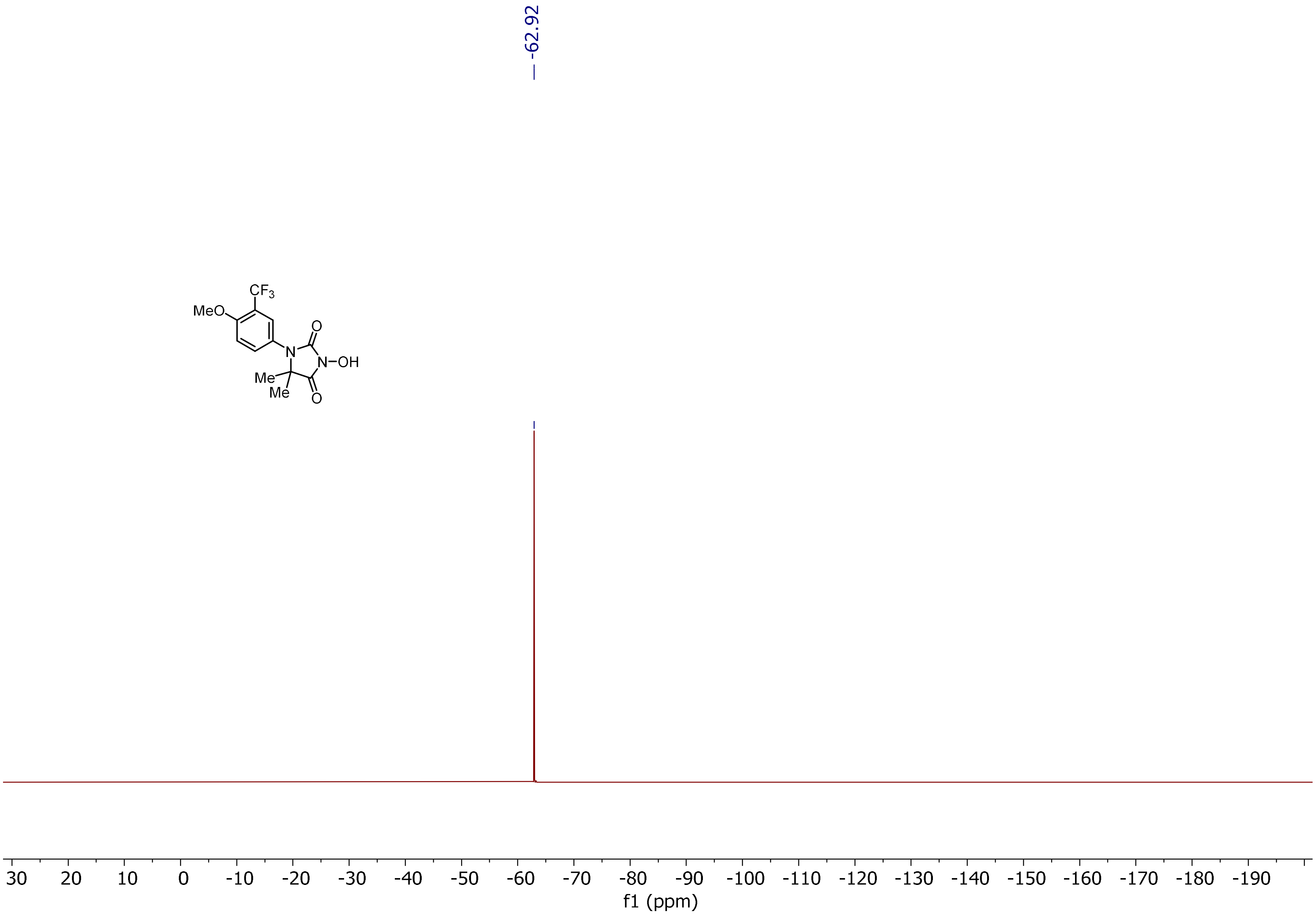

-62.92
CF3
MeO
O
N
N
OH
Me
Me
O
30 20 10 0 -10 -20 -30 -40 -50 -60 -70 -80 -90 -100 -110 -120 -130 -140 -150 -160 -170 -180 -190
f1 (ppm)
